# Novel piperazine–chalcone hybrids and related pyrazoline analogues targeting VEGFR-2 kinase; design, synthesis, molecular docking studies, and anticancer evaluation

**DOI:** 10.1080/14756366.2020.1861606

**Published:** 2020-12-21

**Authors:** Marwa F. Ahmed, Eman Y. Santali, Radwan El-Haggar

**Affiliations:** a Department of Pharmaceutical Chemistry, Faculty of Pharmacy, Taif University, Taif, Kingdom of Saudi Arabia; b Department of Pharmaceutical Chemistry, Faculty of Pharmacy, Helwan University, Cairo, Egypt

**Keywords:** antitumor, vascular endothelial growth factor receptor, molecular docking

## Abstract

New piperazine–chalcone hybrids and related pyrazoline derivatives have been designed and synthesised as potential vascular endothelial growth factor receptor-2 (VEGFR-2) inhibitors. The National Cancer Institute (NCI) has selected six compounds to evaluate their antiproliferative activity *in vitro* against 60 human cancer cells lines. Preliminary screening of the examined compounds indicated promising anticancer activity against number of cell lines. The enzyme inhibitory activity against VEGFR-2 was evaluated and IC_50_ of the tested compounds ranged from 0.57 µM to 1.48 µM. The most potent derivatives **Vd** and **Ve** were subjected to further investigations. A cell cycle analysis showed that both compounds mainly arrest HCT-116 cell cycle in the G2/M phase. Annexin V-FITC apoptosis assay showed that **Vd** and **Ve** induced an approximately 18.7-fold and 21.2-fold total increase in apoptosis compared to the control. Additionally, molecular docking study was performed against VEGFR (PDB ID: 4ASD) using MOE 2015.10 software and **Sorafenib** as a reference ligand.

## Graphical Abstract

**Figure:**
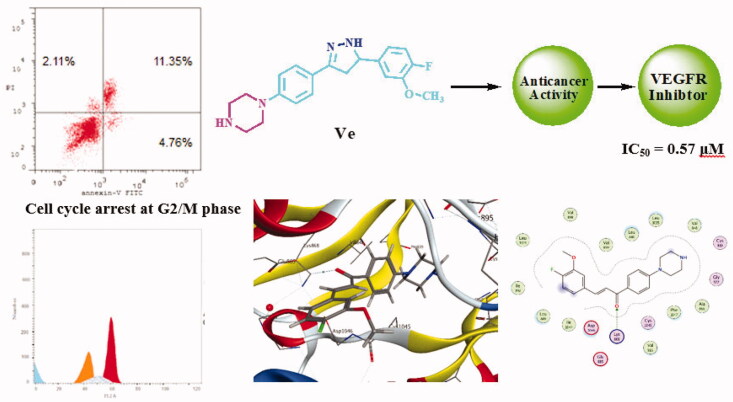

## 1. Introduction

Cancer is a major global health problem characterised by uncontrolled growth of abnormal cells. Although cancer research resulted in a range of innovative and promising approaches, the medications used as therapies have strong drawbacks and cancer is expected to be the leading cause of death in the future^1–3^. Targeted cancer treatments have recently been approved to treat specific cancers such as melanoma, renal, colon, lung, ovary, central nervous system, breast, and leukaemia^4^. Targeted chemotherapy requires several strategies, including angiogenesis inhibition, which has proved to be a successful technique for tumour growth^5^. Angiogenesis is an important physiological process in which the pre-existing vessels form new blood vessels. It is a vital physiological process that happens during inflammation and wound healing^6^^,^^7^. New blood vessels penetrate tumour masses and provide them with oxygen and nutrients which promote tumour progression and metastasis in pathological angiogenesis^8^. Therefore, blocking angiogenesis could be a promising strategy to inhibit growth of tumours with lower adverse effects than other typical chemotherapies^5^.

The vascular endothelial growth factor (VEGF) isoforms were particularly attractive targets to inhibit angiogenesis^9^^,^^10^. The expression of VEGF during embryonic stages is high and is thought to play a crucial role in new (vasculogenesis) or pre-existing blood vessels (angiogenesis)^11^. For certain solid tumours, overexpression of VEGF contributes to increased tumour growth and metastasis, which may be attributed to improved nutrient replenishment availability for the metabolising cell^12^^,^^13^. Vascular endothelial growth factor receptor-2 (VEGFR-2) is a subtype of tyrosine kinase receptor VEGF family (VEGFR-TK)^14^^,^^15^. It is responsible for normal and abnormal changes in vascular endothelial cells^16^^,^^17^. VEGFR-2 inhibition will affect tumour cell blood supply, inhibiting its development, proliferation, and metastasis. VEGFR signalling pathway inhibition is a key therapeutic target for tumour inhibition^18^^,^^19^.

N-aryl piperazine derivatives are important organic compounds that have recently attracted considerable interest for their anti-cancer activity^20–23^. On the other hand, pyrazole a simple aromatic five-membered ring contains two adjacent nitrogen atoms is included in many derivatives that display a variety of pharmacological activities such as anti-Alzheimer disease^24^, anticonvulsant^25^^,^^26^, anti-tubercular^27^^,^^28^, anti-microbial^29^^,^^30^, anti-inflammatory, and analgesic^31^^,^^32^. Numerous pyrazole derivatives have proved their anti-cancer efficacy against different types of cancer^33–38^. Chalcones, (1,3-diaryl-2-propene-1-ones) derivatives that can conventionally be synthesised by Claisen–Schmidt condensation^39^ remained a curiosity among researchers due to their diversified biological activities^40^, such as antimalarial^41^, anti-histaminic^42^, anti-diabetic^43^, anti-inflammatory^44^, and anti-neoplastic activity^45^^,^^46^.

**Figure 1.**
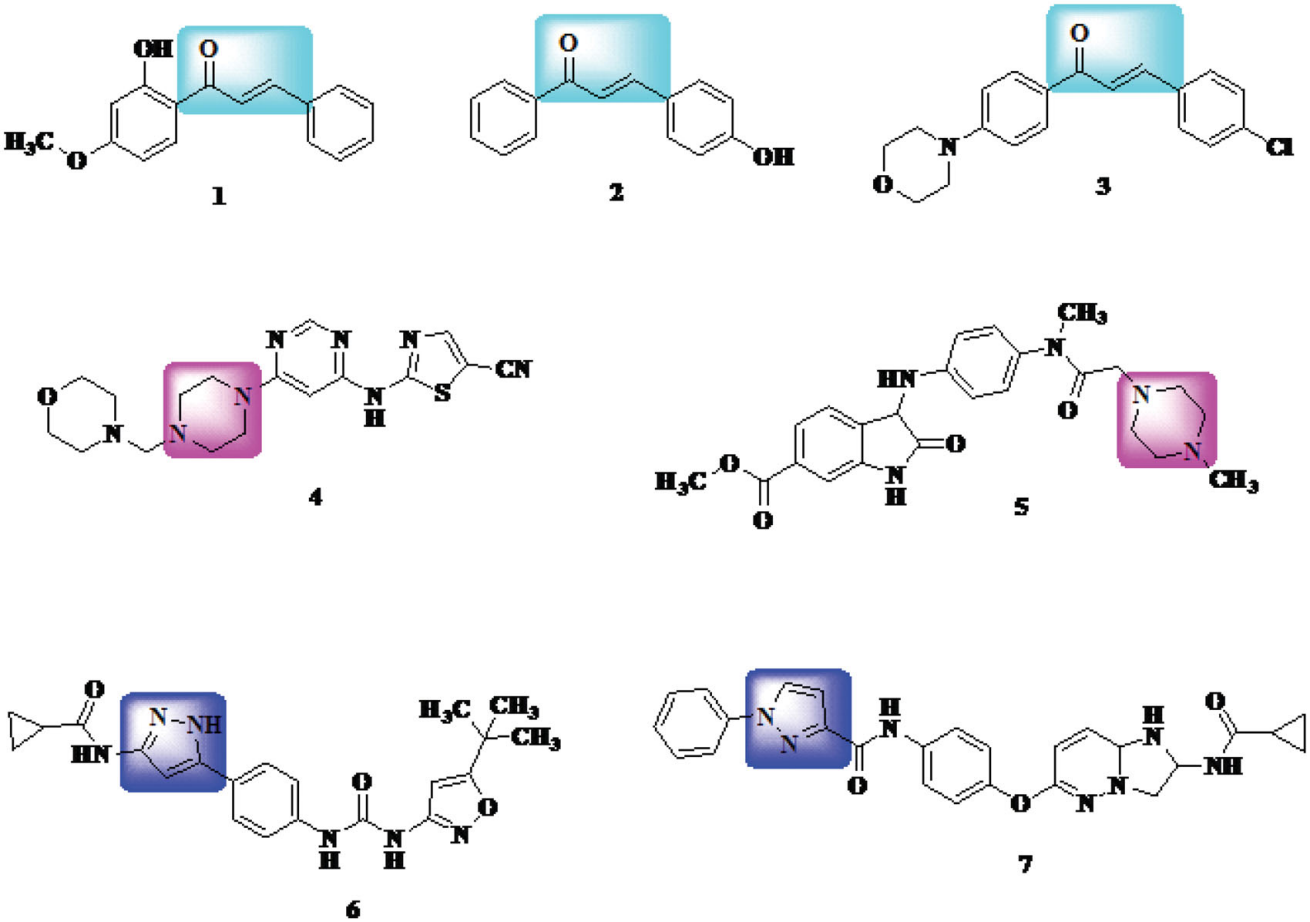
Examples of chalcones, pyrazoles, and piperazine derivatives as VEGFR inhibitors.

On the other hand, many chalcone derivatives (compound **1**^47^, compound **2**^48^, and compound **3**^49^) along with pyrazole derivatives (compound **4**^50^ and compound **5**^51^) and piperazine derivatives (compound **6**^52^ and compound **7**^53^) display potential inhibitory activity of VEGFR-2 kinase which is an essential factor in angiogenesis (Figure 1). Thus, chalcone and analogues demonstrated potential inhibitory activity of VEGFR could be considered as important cancer prevention targets^54–56^.

**Figure 2.**
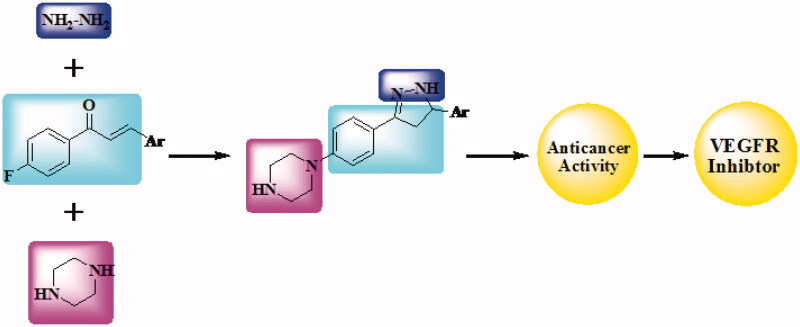
Design of newly synthesised derivatives as VEGFR inhibitor.

Molecular hybridisation is one of the effective chemotherapeutic agent production techniques that require the synthesis of two distinct bioactive units. In the present research, the design and synthesis of novel hybrid compounds bearing piperazine and chalcone or piperazine and pyrazoline (Figure 2) are our target. Newly synthesised compounds have been submitted for evaluation of their anticancer activity to the National Cancer Institute (NCI). Six compounds were selected by NCI, and 60 lines of human cancer cells were screened *in vitro*. The inhibitory activity had been also tested against VEGFR-2.

## 2. Results and discussion

### 2.1. Chemistry

**Scheme 1.**
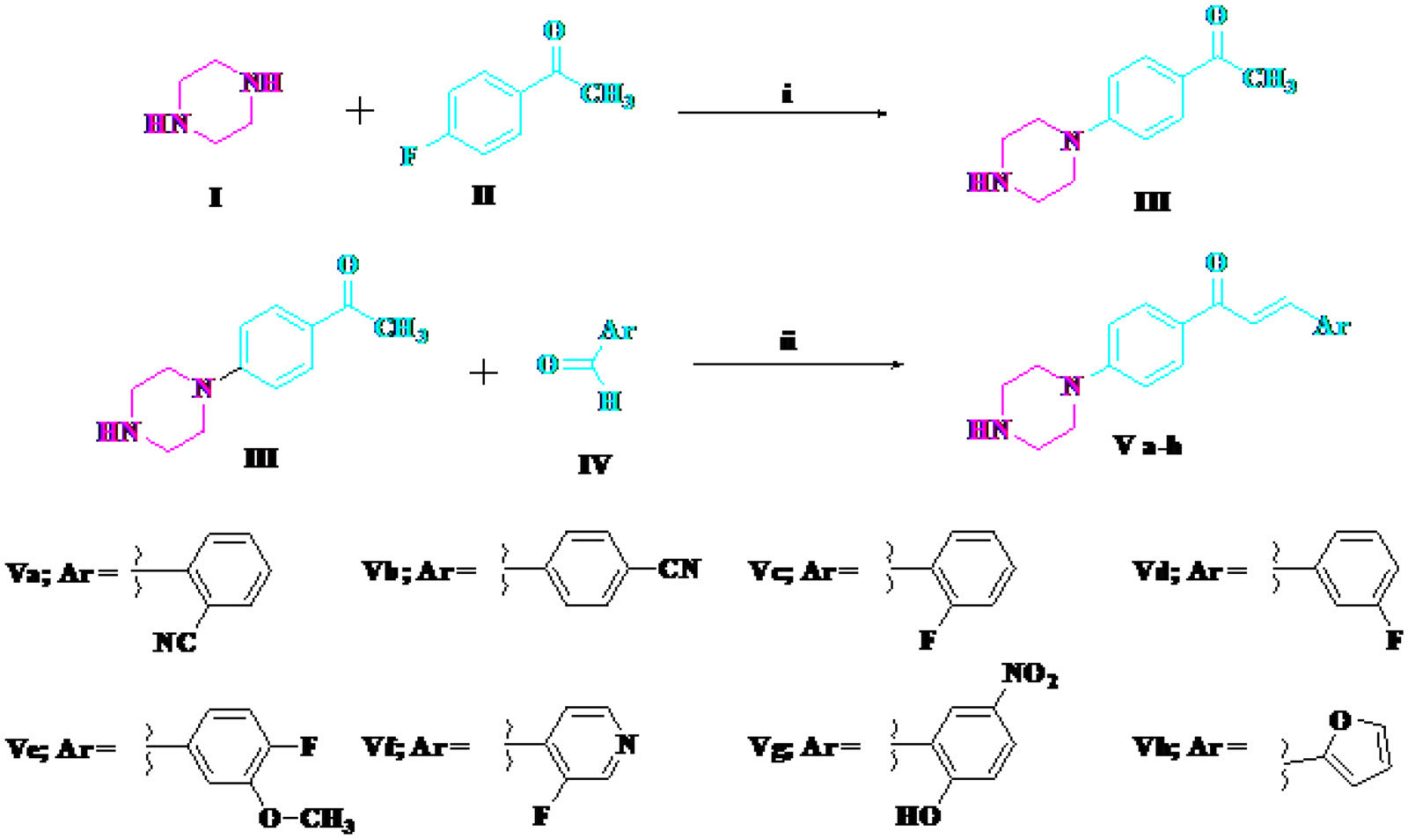
Synthesis of target compounds **Va–h**. Reagents and conditions: (i) DMSO, heating at 110 °C, 24 h; (ii) alcoholic NaOH (10%), stirring 5 h.

**Scheme 2.**
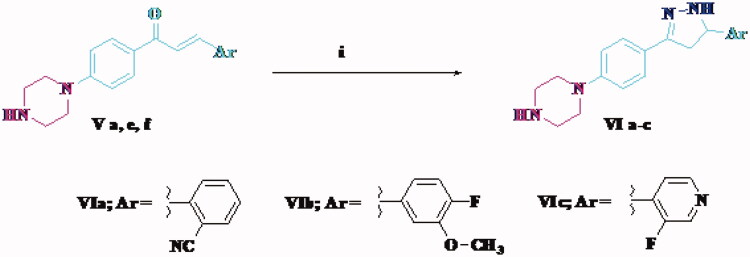
Synthesis of target compounds **VIa–c**. Reagents and conditions: (i) hydrazine hydrate, absolute ethanol, reflux 12 h.

Compounds **Va–h** were synthesised via the reaction of 4′-piperazino-acetophenone **III** and the appropriate aldehyde (Scheme 1). The reaction of the chalcone **Va**, **Ve**, and **Vf** and hydrazine hydrate afforded pyrazoline derivatives **VIa–c** (Scheme 2). The structures of all newly synthesised compounds were confirmed by various methods of spectroscopic analysis, such as ^1^H NMR, ^13^C NMR, IR, and mass spectrometry.

### 2.2. Biological activity

#### 2.2.1. Screening of anticancer activity

Six compounds were selected by the NCI, according to NCI’s DTP selection guidelines^57^, for evaluation of their anticancer activity at a single-dose of 10 µM against 60 human tumours cell lines. The compounds’ screening findings are summarised in Table 1. Data analysis resulting from the primary assay showed that chalcone derivatives **Vd**, **Ve**, and **Vf** showed moderate to good inhibitory activity mainly against leukaemia and colon cancer cell lines.

**Table 1.** *In vitro* growth inhibitory percent for compounds **Vd**, **Ve**, **Vf**, **VIa**, **VIb**, and **VIc** at 10 µM concentration, towards the 60 subpanel cancer cell lines, results were given as a percentage of cell growth promotion.

Panel	Subpanel	Vd	Ve	Vf	VIa	VIb	VIc
Leukaemia	CCRF-CEM	58.18	22.15	78.16	71.21	69.44	80.81
	HL-60(TB)	60.38	28.91	79.28	81.63	76.95	77.32
	K-562	16.55	12.37	85.84	78.12	58.92	77.92
	MOLT-4	68.62	80.75	91.63	96.02	72.10	74.15
	RPMI-8226	76.47	49.59	97.33	88.98	72.01	92.51
	SR	55.72	46.22	92.99	94.43	67.42	90.38
Non-small cell lung cancer	A549/ATCC	76.84	67.25	78.75	78.06	68.21	76.20
	EKVX	95.54	97.12	99.11	101.50	95.41	91.04
	HOP-62	80.17	101.42	102.01	86.44	86.01	83.16
	HOP-92	58.41	55.00	82.34	83.18	57.13	72.42
	NCI-H226	87.11	86.54	99.71	93.10	89.08	82.88
	NCI-H23	77.30	92.63	102.52	91.73	75.49	83.85
	NCI-H322M	75.26	73.01	96.26	98.35	89.15	89.29
	NCI-H460	74.45	97.07	106.01	101.50	95.16	94.92
	NCI-H522	71.57	61.19	78.82	69.51	66.27	81.93
Colon cancer	COLO 205	96.91	83.00	111.89	113.04	93.87	95.69
	HCC-2998	94.99	100.21	100.88	96.75	104.57	101.72
	HCT-116	42.08	27.43	73.87	85.83	61.10	73.82
	HCT-15	73.49	43.16	111.39	112.02	85.57	97.59
	HT29	30.44	49.97	86.42	87.83	20.27	60.16
	KM12	77.75	60.47	105.34	97.48	93.81	96.23
	SW-620	61.08	50.69	105.59	94.11	91.67	93.46
CNS cancer	SF-268	85.41	94.03	100.92	100.24	93.72	107.32
	SF-295	101.78	95.92	101.95	103.66	97.70	98.00
	SF-539	94.49	95.87	101.29	105.04	84.21	104.39
	SNB-19	71.95	59.87	96.07	95.34	95.65	97.45
	SNB-75	85.44	89.05	89.08	90.58	88.85	101.50
	U251	72.39	67.21	95.03	94.54	78.59	95.06
Melanoma	LOX IMVI	71.13	49.01	103.22	99.03	79.56	94.31
	MALME-3M	94.15	112.40	99.98	106.87	102.13	93.43
	M14	81.16	78.43	95.53	104.47	84.68	95.86
	MDA-MB-435	94.27	80.14	102.37	102.57	101.87	105.18
	SK-MEL-2	96.08	85.44	91.63	94.51	87.70	92.72
	SK-MEL-28	109.03	106.02	115.56	114.17	109.51	115.80
	SK-MEL-5	76.85	79.33	101.11	93.27	80.94	85.78
	UACC-257	75.92	76.08	70.86	85.21	80.93	83.07
	UACC-62	79.23	80.13	92.01	88.47	86.00	81.93
Ovarian cancer	IGROV1	67.97	59.94	97.17	93.22	82.04	67.96
	OVCAR-3	93.74	70.18	106.22	101.64	91.15	98.79
	OVCAR-4	80.53	68.87	110.55	101.39	91.49	108.25
	OVCAR-5	94.72	121.73	111.84	113.63	101.51	111.45
	OVCAR-8	67.16	57.23	95.35	90.36	84.09	95.06
	NCI/ADR-RES	109.37	79.31	107.53	102.72	104.69	106.03
	SK-OV-3	79.98	87.98	96.68	96.47	83.58	85.00
Renal cancer	786-0	85.24	87.22	103.38	106.73	94.50	111.09
	A498	95.14	100.79	97.22	93.08	90.59	97.17
	ACHN	84.70	68.11	113.06	104.24	101.47	99.87
	CAKI-1	68.63	79.37	86.18	83.12	86.62	80.62
	RXF 393	75.94	101.92	97.78	108.30	95.32	104.89
	SN12C	68.16	57.67	99.00	98.09	83.08	89.56
	TK-10	114.19	101.24	95.83	130.17	113.60	122.80
	UO-31	76.58	58.35	89.78	86.28	76.56	77.18
Prostate cancer	PC-3	66.01	51.40	73.56	83.35	64.23	70.34
	DU-145	77.70	105.61	108.14	104.00	103.62	107.16
Breast cancer	MCF7	51.72	34.68	99.38	93.05	68.65	73.03
	MDA-MB-231/ATCC	73.60	94.50	98.76	95.96	81.98	84.58
	HS 578T	79.41	91.26	99.19	91.67	84.17	89.15
	BT-549	73.16	66.42	90.81	96.87	70.80	85.33
	T-47D	87.54	74.40	118.12	89.99	73.62	82.20
	MDA-MB-468	94.85	46.48	100.32	101.01	96.57	114.26

As illustrated in Table 1, compound **Vf** bearing 3-fluoropyridine moiety showed weak anti-proliferative activity against non-small cell lung cancer HOP-92, leukaemia K-562, colon cancer HT29, leukaemia SR, and breast cancer MCF7 cancer cell lines, with cell growth promotion (82.34%, 85.84%, 86.42%, 92.99%, 99.38%; cell growth inhibition: 17.66%, 14.16%, 13.85%, 7.01%, and 0.62%, respectively). It also showed moderate activity against leukaemia HL-60(TB) (cell growth promotion 79.28%; cell growth inhibition: 20.72%), leukaemia CCRF-CEM (78.16%; cell growth inhibition: 21.84%), and colon cancer HCT-116 (cell growth promotion 73.87%; cell growth inhibition: 26.13%).

Replacing 3-fluoropyridine of compound **Vf** with 3-fluorophenyl moiety, compound **Vd** significantly increased growth inhibition against number of cancer cell lines, such as leukaemia HL-60(TB), non-small cell lung cancer HOP-92, leukaemia CCRF-CEM, leukaemia SR, and breast cancer MCF7 with percent growth inhibition of 39.17%, 41.59%, 41.82%, 44.28%, and 48.28%, respectively, compared to that of compound **Vf** (20.72%, 17.66%, 21.84%, 7.01%, and 0.62%, respectively). In addition, compound **Vd**, showed good to excellent activity against colon cancer HCT-116, colon cancer HT29, and leukaemia K-562 with percent growth inhibition of 57.92%, 69.56%, and 83.45%, respectively.

Furthermore, replacing 3-fluorophenyl moiety with 4-fluoro-3-methoxyphenyl moiety compound **Ve** markedly increase growth inhibition towards many cancer cell lines. It showed cell growth promotion for non-small cell lung cancer HOP-92 (55.00%; cell growth inhibition: 45.00%), leukaemia SR (46.22%; cell growth inhibition: 53.78%), breast cancer MCF7 (34.68%; cell growth inhibition: 65.32%), leukaemia HL-60(TB) (28.91%; cell growth inhibition: 71.09%), colon cancer HCT-116 (27.43%; cell growth inhibition: 72.57%), leukaemia CCRF-CEM (22.15%; cell growth inhibition: 77.85%), and leukaemia K-562 (12.37%; cell growth inhibition: 87.63%). From previous results, we can conclude that chalcone derivative bearing 4-fluoro-3-methoxyphenyl moiety (**Ve**) was the most potent chalcone towards leukaemia CCRF-CEM, leukaemia HL-60(TB), leukaemia K-562, leukaemia SR non-small cell lung cancer HOP-92, colon cancer HCT-116, and breast cancer MCF7 cell lines.

On the other hand, pyrazole analogues **VIa**, **VIb**, and **VIc** showed promising cytotoxicity towards a variety of cancer cell lines. Compound **VIa** bearing benzonitrile moiety exhibited a weak growth inhibition against several cancer cell lines as it displayed cell growth promotion for leukaemia SR (94.43%; cell growth inhibition: 5.57%), colon cancer HT29 (87.83%; cell growth inhibition: 12.17%), colon cancer HCT-116 (85.83%; cell growth inhibition: 14.17%), and non-small cell lung cancer HOP-92 (83.18%; cell growth inhibition: 16.82%). In addition, it showed good inhibitory activity against many cancer cell lines with cell growth promotion for leukaemia K-562 (78.12%; cell growth inhibition: 21.88%), non-small cell lung cancer A549/ATCC (78.06%; cell growth inhibition: 21.94%), leukaemia CCRF-CEM (71.21%; cell growth inhibition: 28.79%), and non-small cell lung cancer NCI-H522 (69.51%; cell growth inhibition: 30.49%).

Replacing benzonitrile moiety with 3-fluoropyridine moiety, compound **VIc** increased anti-proliferative activity against few cancer cell lines such as colon cancer HCT-116, non-small cell lung cancer HOP-92 and colon cancer HT29 with percent of cell growth inhibition of 26.18%, 27.58%, and 39.84%, respectively.

Finally, compound **VIb** with 4-fluoro-3-methoxyphenyl moiety was the most potent pyrazole derivative and exhibited moderate to good inhibitory activity towards many cancer cell lines. It showed percent of cell growth promotion for colon cancer HCT-116, leukaemia K-562, non-small cell lung cancer HOP-92, and colon cancer HT29 of 61.10%, 58.92%, 57.13%, and 20.27%, respectively (cell growth inhibition: 38.9%, 41.08%, 42.87%, and 79.73%, respectively).

#### 2.2.2. Vascular endothelial growth factor receptor-2 inhibition

VEGFR is considered as an important target for the development of potential anti-cancer candidates^54–56^. Therefore, compounds **Vd**, **Ve**, **Vf**, **VIa**, **VIb**, and **VIc** were further investigated for their ability to inhibit VEGFR-2 using colorimetric assay of human VEGFR-2 ELISA (enzyme-linked immunosorbent assay) and **Sorafenib** as a reference drug. Results were presented as half maximal inhibitory concentration (IC_50_) values (Table 2). IC_50_ of the tested compounds ranged from 0.57 µM to 1.48 µM. Compound **Ve** was the most potent VEGFR-2 inhibitor among the tested compounds with an IC_50_ value of 0.57 µM which was comparable to results of **Sorafenib** that had IC_50_=0.51 µM. In addition, compound **Vd** showed significant VEGFR-2 inhibitory activity with IC_50_=0.80 µM. These results supported that VEGFR-2 could be a possible target for anti-tumour activity of our tested compounds.

**Table 2.** *In vitro* VEGFR-2 inhibitory assay.

Compound	IC_50_ (μM)
**Vd**	0.80
**Ve**	0.57
**Vf**	1.33
**VIa**	1.48
**VIb**	1.06
**VIc**	1.31
**Sorafenib**	0.51

IC_50_ values of compounds **Vd**, **Ve**, **Vf**, **VIa**, **VIb** and **VIc** and **Sorafenib** reference drug.

#### 2.2.3. Cell cycle analysis

**Figure 3.**
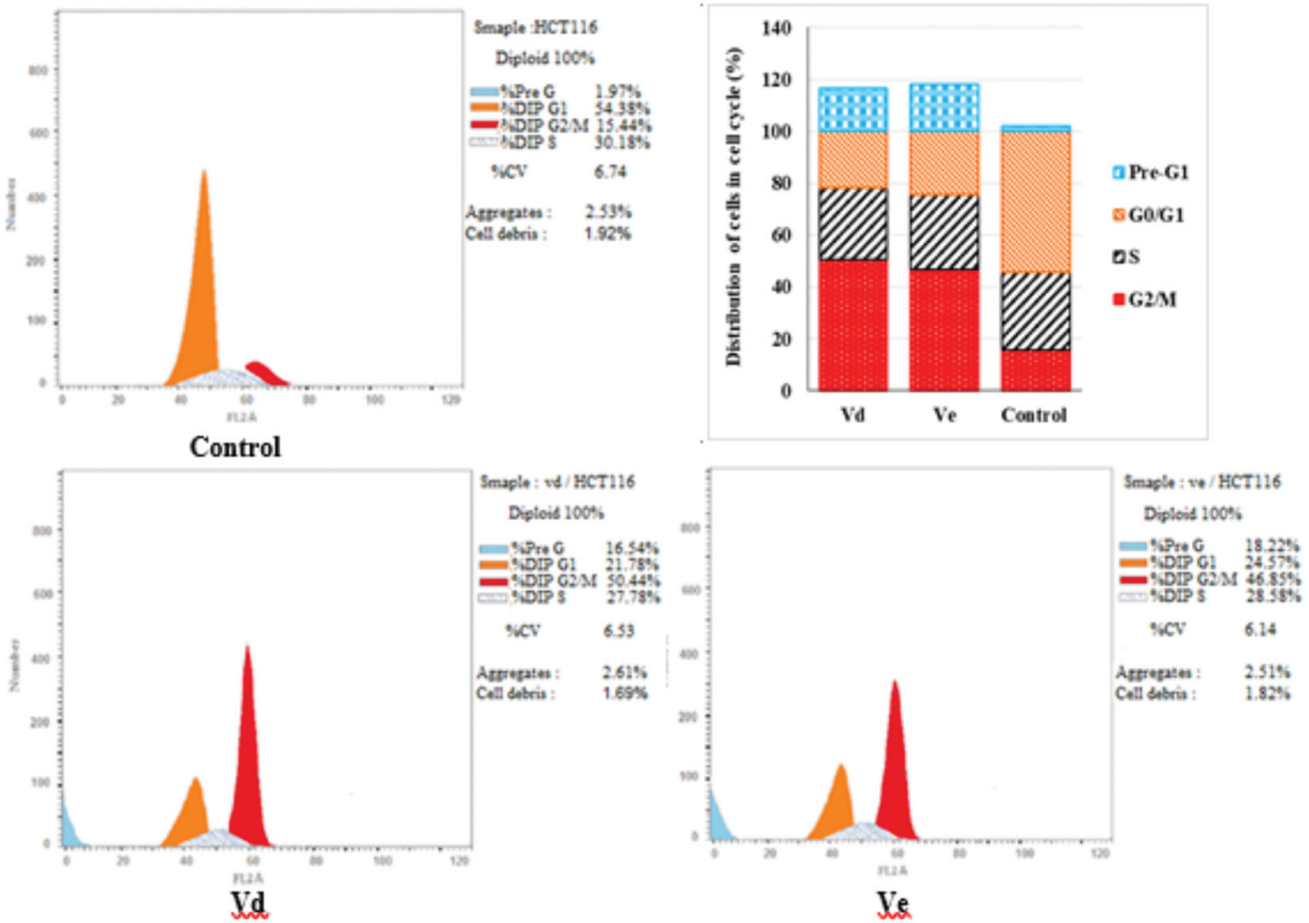
Effect of compounds **Vd** and **Ve** on the phases of cell cycle of HCT-116 cells.

Most of cytotoxic compounds exert their anti-proliferative effect via arresting the cell cycle at certain phase. Flow cytometric analysis is considered a valuable method for determining and analysing the cell cycle parameters^58^. In this study, compounds **Vd** and **Ve** as the most potent derivatives were selected to explore their effect on cell cycle progression and induction of apoptosis in HCT-116 cell line using the standard concentration of 10 µM. The effect on the cell cycle distribution was assessed by a DNA flow cytometry analysis and the cell cycle parameters were compared to untreated control cells in HCT-116 cells which had been incubated with 10 µM of **Vd** and **Ve** compounds and the results are shown in Table 3 and Figure 3. The results revealed that, the percentage of HCT-116 cells at G2/M phase markedly increased from 15.44% to 50.44% and 46.85% after incubation with compound **Vd** and **Ve**, respectively. On the other hand, the percentage of HCT-116 cells at G1 phase decreased from 54.38% in control to 21.78% for compound **Vd** and 24.57% for compound **Ve** indicating that compounds **Vd** and **Ve** induced cell arrest at G2/M phase. The percentage of cell death at pre-G1 phase for compounds **Vd** and **Ve** was 16.54% and 18.22%, respectively.

**Table 3.** Effect of compounds **Vd** and **Ve** on the phases of cell cycle of HCT-116 cells.

Code	%G2/M	%S	%G0/G1	%Pre-G1	Comment
**Vd**	50.44	27.78	21.78	16.54	cell cycle arrest@G2/M
**Ve**	46.85	28.58	24.57	18.22	cell cycle arrest@G2/M
Control	15.44	30.18	54.38	1.97	

#### 2.2.4. Annexin V-FITC apoptosis assay

**Figure 4.**
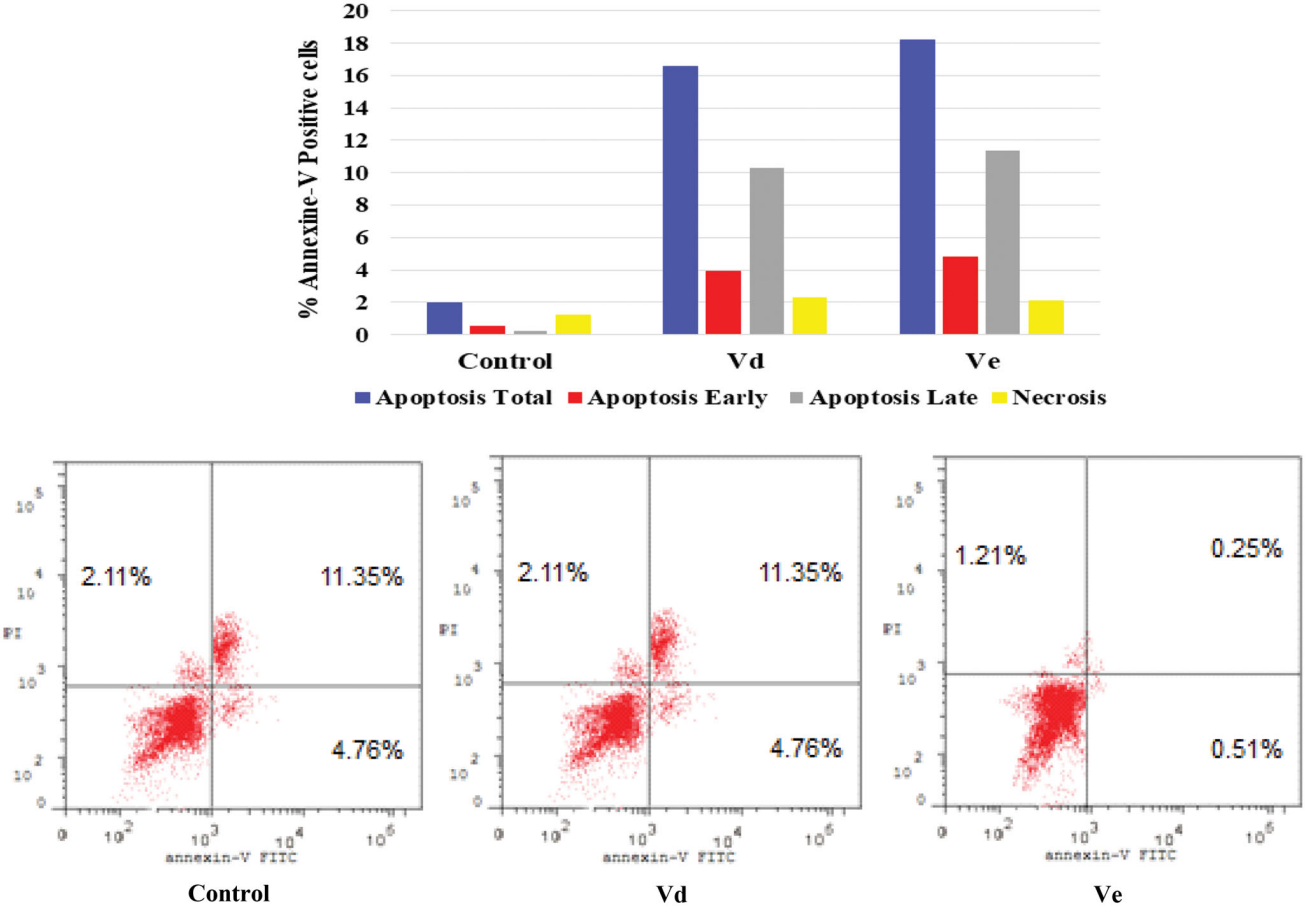
Effect of compounds **Vd** and **Ve** on the percentage of annexin V-FITC-positive staining in HCT-116 cells. The experiments were done in triplicates. The four quadrants identified as: LL: viable; LR: early apoptotic; UR: late apoptotic; UL: necrotic.

Double staining assay of annexin-V/propidium iodide (PI) was used to investigate the mode of induced HCT-116 cell death when treated with the tested compounds **Vd** and **Ve**. HCT-116 cells were treated for 24 h with 10 µM from each tested compound. The results obtained are outlined in Table 4 and Figure 4. The percentage of apoptosis caused by the **Vd** and **Ve** compounds respectively was (16.54 and 18.22). We can also conclude that in early stage treatment of HCT-116 cells with **Vd** and **Ve** compounds results in an increase in the percentage of apoptotic cells from 0.51% for control untreated cells to be 3.92 and 4.76, respectively. In late stage, the percentage of apoptotic cells was 10.32–11.35% compared to control (0.25%). The results indicate that the tested **Vd** and **Ve** induced apoptosis in HCT-116 cell line.

**Table 4.** Apoptosis and necrosis percent induced by compounds **Vd** and **Ve** in HCT-116 cells.

	Apoptosis			Necrosis
	Total	Early	Late	
**Vd**	16.54	3.92	10.32	2.3
**Ve**	18.22	4.76	11.35	2.11
Control	1.97	0.51	0.25	1.21

### 2.3. Molecular docking

Molecular modelling is considered as an important tool to study molecular interactions of certain ligands and binding site of the corresponding protein. The ligand–protein interaction behaviour at the active site was estimated based on the docking score function as implemented in MOE 2015.10^59^. In this study, six active potential anticancer compounds **Vd**, **Ve**, **Vf**, **VIa**, **VIb**, and **VIc** were subjected to molecular docking studies using MOE program on the 3D structure of VEGFR using **Sorafenib** as reference compound. Binding free energy data obtained after the docking procedure showed that the tested compounds exhibit favourable docked complexes with the active site of target protein. The tested compounds **Vd**, **Ve**, **Vf**, **VIc**, **VIb**, and **VIc** exhibited interactions with the VEGFR active site to different extents and the docking score free energy of the tested compounds found to be this order: **Ve**>**VIa**>**Vd**>**Vf**>**VIc**>**VIb**, as shown in Table 5. Also, the other scoring parameters such as rmsd_refine, E_conf, E_place, and E_refine, indicated that the tested compounds were correctly docked in the biding site as the reference ligand **Sorafenib**.

**Table 5.** Docking energy scores (kcal/mol) derived from the MOE for compounds **Vd**–**f**, **VIa**–**c**, and the reference ligand **Sorafenib**.

Comp. no.	Score	rmsd_refine	E_conf	E_place	E_score1	E_score2	E_refine
**Vd**	–7.3974	2.6436	89.8860	–55.3257	–13.3058	–7.3974	–30.4426
**Ve**	–7.9154	1.4800	118.5701	–66.3756	–12.5551	–7.9154	–33.5940
**Vf**	–6.9883	1.5306	101.3355	–81.8762	–11.3026	–6.9883	–27.0719
**VIa**	–7.4371	1.3288	121.3822	–71.7816	–13.2958	–7.4371	–20.1583
**VIb**	–6.7455	0.9160	103.8081	–80.8748	–12.5471	–6.7455	–16.9364
**VIc**	–6.9328	1.2209	99.4951	–46.3408	–11.6422	–6.9328	–22.0011
**Sorafenib**	–11.1354	1.0623	–57.2969	–94.4447	–12.8634	–11.1354	–69.0130

Score: lower scores are more favourable; rmsd_refine: the root mean square deviation of the pose from the docking pose compared to the co-crystal ligand position; E_conf: free binding energy of the conformer; E_place: free binding energy from the placement stage; E_score 1: free binding energy from the first rescoring stage; E_score 2: free binding energy from the second rescoring stage; E_refine: free binding energy from the refinement stage.

**Figure 5.**
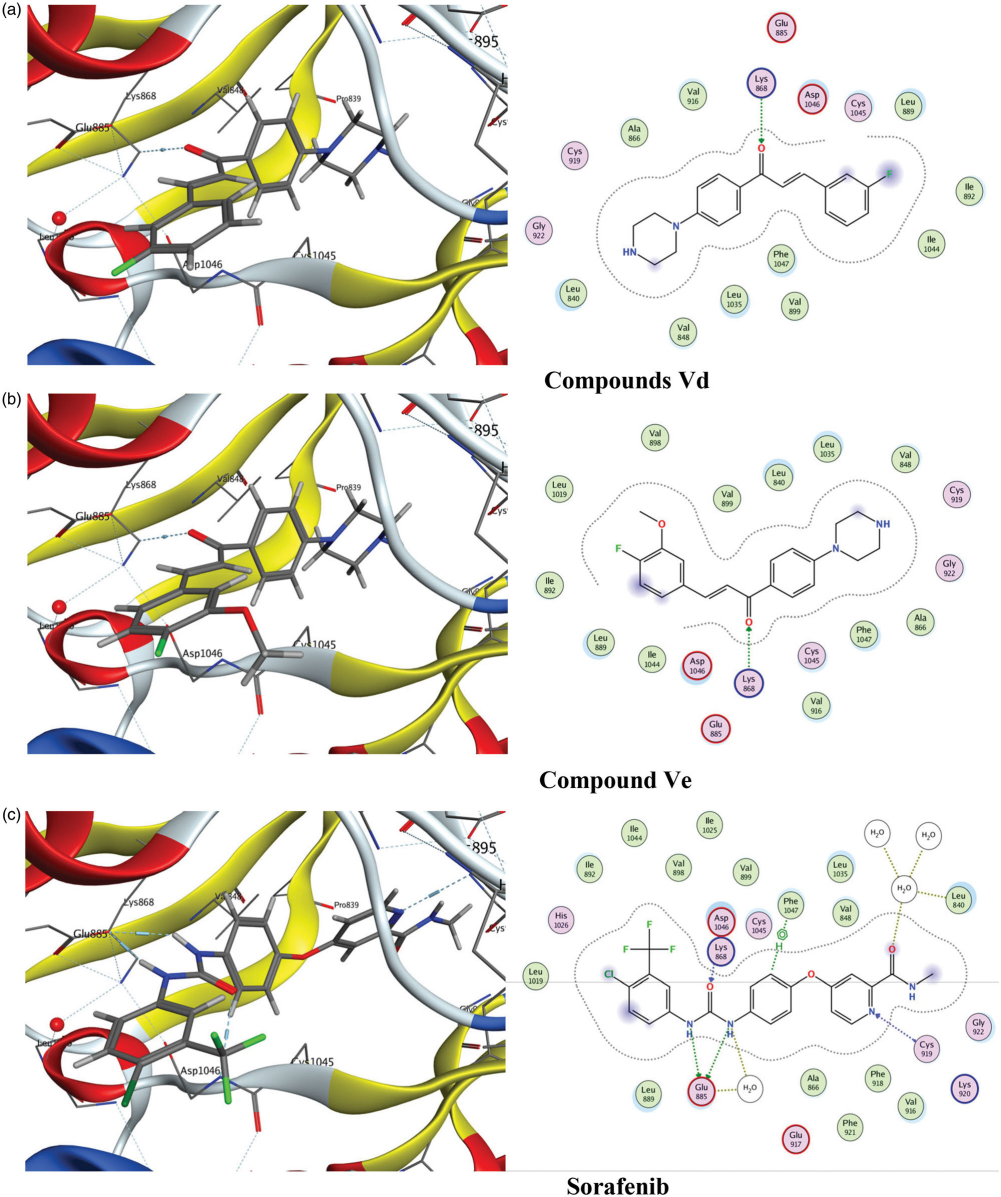
Docking of compounds **Vd**, **Ve** and the reference ligand **Sorafenib** into VEGFR active sites. (a) Compounds **Vd**. (b) Compound **Ve**. (c) **Sorafenib**.

Based on the obtained scoring results, compounds **Ve**, **VIa**, and **Vd** showed the highest binding affinity to the VEGFR-2 active site. Compound **Ve** was the best among the synthesised compounds with docking score (–7.9154 kcal/mol) compared to the reference ligand **Sorafenib** docking score (–11.1354 kcal/mol), and it formed a direct interaction in the active site, similar to that of **Sorafenib**. Also, compounds **VIa** and **Vd** showed a good docking score (–7.4371 kcal/mol) and (–7.3974 kcal/mol), respectively, compared to **Sorafenib**. Figure 5 reveals that the tested compounds **VIa** and **Vd** along with **Sorafenib** reacted with important amino acids in the active binding site. The carbonyl group of the chalcone moiety in both compounds **Ve** and **Vd** acts as H-bond acceptor and formed a H-bonding with LYS868 (Figure 5(a,b)). On the other hand, the reference compound **Sorafenib** formed several hydrogen bonding with the active side nearby amino acids GLU885, CYS919, ASP1046, and PHE1047 (Figure 5(c)).

## 3. Experimental

### 3.1. Chemistry

All melting points were uncorrected and measured by Electro thermal IA 9000 series digital melting point apparatus at the Micro-analytical Center, Cairo University (Giza, Egypt). IR spectra had been reported on FT. IR 670-Nicolet spectrophotometer-Nexus (Thermo Scientific, Madison, WT); determination of ^1^H NMR and ^13^C NMR spectra on a JEOL AS NMR spectrometer (Tokyo, Japan). Mass spectra were measured on Finnigan Mat SSQ 7000 mode EI 70 ev (Thermo Inst. Sys. Inc., Waltham, MA). Thin-layer chromatography was performed using chloroform/methanol (10:1, v/v) on thin-layer chromatographic plates of silica gel 60 F254 (Merck, Kenilworth, NJ), and the spots were observed for a few seconds by exposure to UV lamps at *λ*254 nm and used to monitor the reaction time. 1-(4-(Piperazin-1-yl)phenyl)ethan-1-one III was prepared as reported method^60^.

### 3.2. General method for preparation of (Va–h)

A mixture of 4′-piperazinoacetophenone **III** (0.01 mol) and the corresponding aldehyde derivatives **IV**, namely, 2-cyanobenzaldehyde, 4-cyanobenzaldehyde, 2-flurobenzaldehyde, 3-flurobenzaldehyde, 4-fluro-3-methoxy benzaldehyde, 3-fluroisonicotinaldehyde, 2-hydroxyl-5-nitrobenzaldehyde, and 2-furaldehyde (0.01 mol) was dissolved in 10% alcoholic sodium hydroxide (25 mL) and stirred for 5 h at room temperature. The precipitate was filtered, washed with water, dried, and crystallised from ethanol to give the target compounds **Va–h**, respectively.

#### 3.2.1. 2-(3-Oxo-3-(4-(piperazin-1-yl)phenyl)prop-1-enyl)benzonitrile (Va)

Yield 70%, m.p. 150 °C. Analysis calculated for C_20_H_19_N_3_O; Calc.: % C, 75.69; H, 6.03; N, 13.24; found: % C, 75.74; H, 6.12; N, 13.10. IR: *υ*_max._/cm^−m^ 3230 (NH), 3020 (C–H aromatic), 2200 (CN), 1700 (C=O), 1580 (C=C). ^13^C NMR (DMSO-d_6_): 189, 145, 138, 137,133, 132, 130, 128, 127, 126, 121, 112, 115, 109, 54, 45. ^1^H NMR: *δ* 2.1 (s, 1H, NH, D_2_O exchangeable), 2.4–3.0 (m, 8H, piperazinyl protons), 6.8 (d, 1H, *J*= 15.0 Hz, CH═), and 7.0–8.0 (m, 9H, Ar-H, CH═). MS: *m/z* (% relative intensity)=317 (M^+^, 20%), 215 (100%).

#### 3.2.2. 4-(3-Oxo-3–(4-(piperazin-1-yl)phenyl)prop-1-enyl)benzonitrile (Vb)

Yield 75%, m.p. 170 °C. Analysis calculated for C_20_H_19_N_3_O; calcd.: % C, 75.69; H, 6.03; N, 13.24; found: % C, 75.72; H, 6.10; N, 13.15. IR: *υ*_max._/cm^−1^ 3240 (NH), 3010 (C–H aromatic), 2210 (CN), 1720 (C═O), 1590 (C═C). ^13^C NMR (DMSO-d_6_): 190, 149, 139, 136,133, 132, 128, 127, 121, 118, 112, 111, 55, 46. ^1^H NMR: *δ* 2.0 (s, 1H, NH, D_2_O exchangeable), 2.5–3.1 (m, 8H, piperazinyl protons), 6.8 (d, 1H, *J*= 15.2 Hz, CH═), and 7.3–8.1 (m, 9H, Ar-H, CH═). MS: *m/z* (% relative intensity)=317 (M^+^, 7%), 77 (100%).

#### 3.2.3. 3-(2-Fluorophenyl)-1-(4-(piperazin-1-yl)phenyl)prop-2-en-1- one (Vc)

Yield 70%, m.p. 176 °C. Analysis calculated C_19_H_19_FN_2_O; calcd.: % C, 73.53; H, 6.17; N, 9.03; found: % C, 73.49; H, 6.34; N, 9.12. IR: *υ*_max._/cm^−1^ 3310 (NH), 3000 (C–H aromatic), 1720 (C═O), 1590 (C═C). ^13^C NMR (DMSO-d_6_): 192, 161, 145, 138, 132, 130, 129, 127, 125, 123, 121, 115, 112, 55, 46. ^1^H NMR: *δ* 2.2 (s, 1H, NH, D_2_O exchangeable), 2.7–3.3 (m, 8H, piperazinyl protons), 6.7 (d, 1H, *J*= 15.5 Hz, CH═), and 7.1–7.9 (m, 9H, Ar-H, CH═). MS: *m/z* (% relative intensity)=310 (M^+^, 50%), 291 (100%).

#### 3.2.4. 3-(3-Fluorophenyl)-1-(4-(piperazin-1-yl)phenyl)prop-2-en-1- one (Vd)

Yield 80%, m.p. 200 °C. Analysis calculated C_19_H_19_FN_2_O; calcd.: % C, 73.53; H, 6.17; N, 9.03; found: % C, 73.59; H, 6.25; N, 9.10. IR: *υ*_max._/cm^−1^ 3315 (NH), 3010 (C–H aromatic), 1720 (C═O), 1600 (C═C). ^13^C NMR (DMSO-d_6_): 189, 162, 145, 137, 136, 133, 131, 127, 124, 122, 115, 114, 113, 55, 47. ^1^H NMR: *δ* 2.3 (s, 1H, NH, D_2_O exchangeable), 2.6–3.1 (m, 8H, piperazinyl protons), 6.8 (d, 1H, *J*= 15.0 Hz, CH═), and 7.2–7.7 (m, 9H, Ar-H, CH═). MS: *m/z* (% relative intensity)=310 (M^+^, 100%).

#### 3.2.5. 3-(4-Fluoro-3-methoxyphenyl)-1-(4-(piperazin-1-yl)phenyl) prop-2-en-1-one (Ve)

Yield 70%, m.p. 180 °C. Analysis calculated C_20_H_21_FN_2_O_2_; calcd.: % C, 70.57; H, 6.22; N, 8.23; found: % C, 70.50; H, 6.19; N, 8.27. IR: *υ*_max._/cm^−1^ 3400 (NH), 3050 (C–H aromatic), 1730 (C═O), 1600 (C═C). ^13^C NMR (DMSO-d_6_): 190, 151, 149, 145, 137, 132, 131, 127, 123, 121, 117, 113, 112, 57, 50, 45. ^1^H NMR: *δ* 2.4 (s, 1H, NH, D_2_O exchangeable), 2.8–3.3 (m, 8H, piperazinyl protons), 3.80 (s, 3H, OCH_3_), 7.0 (d, 1H, *J*= 15.1 Hz, CH═), and 7.1–7.8 (m, 8H, Ar-H, CH═). MS: *m/z* (% relative intensity)=340 (M^+^, 24%), 85 (100%).

#### 3.2.6. 3-(3-Fluoropyridin-4-yl)-1-(4-(piperazin-1-yl)phenyl)prop-2- en-1-one (Vf)

Yield 75%, m.p. 225 °C. Analysis calculated C_18_H_18_FN_3_O; calcd.: % C 69.44; H, 5.83; N, 13.50; found: % C, 69.49; H, 5.95; N, 13.61. IR: *υ*_max._/cm^−1^ 3450 (NH), 3040 (C–H aromatic), 1720 (C═O), 1590 (C═C). ^13^C NMR (DMSO-d_6_): 187, 156, 146, 144, 139, 136, 133, 131, 128, 126, 118, 113, 53, 45. ^1^H NMR: *δ* 2.3 (s, 1H, NH, D_2_O exchangeable), 2.7–3.4 (m, 8H, piperazinyl protons), 6.7 (d, 1H, *J*= 15.0 Hz, CH═CH), and 6.9–7.8 (m, 8H, Ar-H, CH═). MS: *m/z* (% relative intensity)=311 (M^+^, 100%).

#### 3.2.7. 3-(2-Hydroxy-5-nitrophenyl)-1-(4-(piperazin-1-yl)phenyl)prop-2-en-1-one (Vg)

Yield 65%, m.p. 185 °C. Analysis calculated C_19_H_19_N_3_O_4_; calcd.: % C 64.58; H, 5.42; N, 11.89; found: % C, 64.50; H, 5.37; N, 11.83. IR: *υ*_max._/cm^−1^ 3400 (NH), 3320 (OH), 3010 (C–H aromatic), 1700 (C═O), 1590 (C═C). ^13^C NMR (DMSO-d_6_): 195, 163, 141, 140, 139, 131, 129, 126, 125, 120, 119, 116, 112, 54, 45. ^1^H NMR: *δ* 2.0 (s, 1H, NH, D_2_O exchangeable), 2.4–3.1 (m, 8H, piperazinyl protons), 6.6 (d, 1H, *J*= 15.2 Hz, CH═), 7.3–7.9 (m, 8H, Ar-H, CH═), and 9.1 (s, 1H, O-H). MS: *m/z* (% relative intensity)=353 (M^+^, 10%), 290 (100%).

#### 3.2.8. 3-(Furan-2-yl)-1-(4-(piperazin-1-yl)phenyl)prop-2-en-1-one (Vh)

Yield 70%, m.p. 190 °C. Analysis calculated C_17_H_18_N_2_O_2_; calcd.: % C 72.32; H, 6.43; N, 9.92; found: % C 72.37; H, 6.52; N, 9.90. IR: *υ*_max._/cm^−1^ 3390 (NH), 3030 (C–H aromatic), 1700 (C═O), 1600 (C═C). ^13^C NMR (DMSO-d_6_): 188, 151, 140, 137, 130, 127, 126, 120, 114, 112, 110, 50, 45. ^1^H NMR: *δ* 2.1 (s, 1H, NH, D_2_O exchangeable), 2.9–3.5 (m, 8H, piperazinyl protons), 6.7 (d, 1H, *J*= 15.5 Hz, CH═), and 7.0–7.8 (m, 8H, Ar-H, CH═). MS: *m/z* (% relative intensity)=282 (M^+^, 30%), 197 (100%).

### 3.3. General method for the preparation of VIa–c

A mixture of the chalcone **Va**, **Ve**, and **Vf** (0.006 mol) and hydrazine hydrate (0.006 mol, 98%) in absolute ethanol (30 mL) was heated for 12 h under reflux. The reaction was cooled, the formed precipitate was filtered off and crystallised from ethanol to give compounds **VIa–c**, respectively.

#### 3.3.1. 2-(3-(4-(Piperazin-1-yl)phenyl)-4,5-dihydro-1H-pyrazol-5-yl)benzonitrile (VIa)

Yield 64%, m.p. 165 °C. Analysis calculated C_20_H_21_N_5_; calcd.: % C 72.48; H, 6.39; N, 21.13; found: % C 72.54; H, 6.42; N, 21.10. IR: *υ*_max._/cm^−1^ 3320 (NH), 3030 (C–H aromatic), 2220 (CN), 1600 (C═C). ^13^C NMR (DMSO-d_6_): 151, 146, 134, 133, 131, 129, 128, 127, 125, 115, 112, 111, 54, 47, 45, 42. ^1^H NMR: *δ* 2.1 (s, 1H, NH, D_2_O exchangeable), 2.4–2.9 (m, 8H, piperazinyl protons), 3.3 (dd, 1H, *J*= 11.4, 5.1 Hz, pyrazoline), 3.4 (dd, 1H, *J*= 11.3, 6.1 Hz, pyrazoline), 5.0 (dd, 1H, *J* = 11.0, 5.5 Hz, pyrazoline), 7.0–7.7 (m, 8H, Ar-H), and 9.0 (s, NH, D_2_O exchangeable). MS: *m/z* (% relative intensity)=331 (M^+^, 14%), 77 (100%).

#### 3.3.2. 1-(4-(5-(4-Fluoro-3-methoxyphenyl)-4,5-dihydro-1H-pyrazol- 3-yl)phenyl) piperazine (VIb)

Yield 70%, m.p. 195 °C. Analysis calculated C_20_H_23_FN_4_O; calcd.: % C 67.78; H, 6.54; N, 15.81; found: % C 67.70; H, 6.59; N, 15.74. IR: *υ*_max._/cm^−1^ 3400 (NH), 3040 (C–H aromatic), 1590 (C═C). ^13^C NMR (DMSO-d_6_): 152, 150, 148, 140, 133, 130, 125, 120, 116, 112, 111, 56, 52, 50, 46, 43. ^1^H NMR: *δ* 2.3 (s, 1H, NH, D_2_O exchangeable), 2.6–3.0 (m, 8H, piperazinyl protons), 3.2 (dd, 1H, *J=* 11.3, 6.1 Hz, pyrazoline), 3.3 (dd, 1H, *J*= 11.7, 5.9 Hz, pyrazoline), 3.8 (s, 3H, OCH_3_), 5.1 (dd, 1H, *J*= 11.9, 6.1 Hz, pyrazoline), 6.9–7.5 (m, 7H, Ar-H), and 8.5 (s, NH, D_2_O exchangeable). MS: *m/z* (% relative intensity)=354 (M^+^, 100%).

#### 3.3.3. 1-(4-(5-(3-Fluoropyridin-4-yl)-4,5-dihydro-1H-pyrazol-3-yl) phenyl) piperazine (VIc)

Yield 70%, m.p. 135 °C. Analysis calculated C_18_H_20_FN_5_; calcd.: % C 66.44; H, 6.20; N, 21.52; found: % C 66.49; H, 6.25; N, 21.548 IR: *υ*_max._/cm^−1^ 3390 (NH), 3020 (C–H aromatic), 1595 (C═C). ^13^C NMR (DMSO-d_6_): 152, 150, 147, 139, 138, 133, 130, 125, 124, 111, 54, 47, 45, 44. ^1^H NMR: *δ* 2.3 (s, 1H, NH, D_2_O exchangeable), 2.7–3.2 (m, 8H, piperazinyl protons), 3.4 (dd, 1H, *J* = 11.2, 5.0 Hz, pyrazoline), 3.6 (dd, 1H, *J*= 11.6, 5.9 Hz, pyrazoline), 5.2 (dd, 1H, *J*= 12.0, 5.7 Hz, pyrazoline), 7.0–7.9 (m, 7H, Ar-H), and 8.8 (s, NH, D_2_O exchangeable). MS: *m/z* (% relative intensity)=325 (M^+^, 19%), 229 (100%).

### 3.4. *In vitro* cytotoxicity

*In vitro* cytotoxicity was performed in NCI according to reported method^61^.

### 3.5. VEGFR-2 inhibition assay

IC_50_s of **Vd**, **Ve**, **Vf**, **VIa**, **VIb**, and **VIc** compounds were evaluated *in vitro* using colorimetric assay of human VEGFR-2 ELISA (enzyme-linked immunosorbent assay) kits (HTScan^®^ VEGF Receptor 2 Kinase Assay Kit). It includes active VEGFR-2 kinase (a biotinylated peptide substrate and a phospho-tyrosine antibody) for detection of the phosphorylated form of the substrate peptide. On a 96-well plate, a particular VEGFR-2 antibody was seeded and 100 µL of the normal solution or compound tested was applied, incubated at room temperature for 2.5 h and washed.

Then, 100 µL of the prepared biotin antibody was added, incubated for an additional 1 h at room temperature and washed. Following, 100 µL of streptavidin solution was added at room temperature, incubated for 45 min and then, 100 µL of TMB substrate solution was applied and incubated at room temperature for 30 min. Finally, 50 µL stop solution was added and the absorption was measured at 450 nm instantly. The standard curve, the *X*-axis concentrations, and the *Y*-axis absorbance were drawn.

### 3.6. Cell cycle analysis

HCT-116 cells were seeded at concentrations of 1 × 10^5^ cells per well in a six-well plate, then incubated for 24 h. The cells were treated for 24 h with vehicles (0.1% DMSO) or 10 µM of **Vd** or **Ve** compounds. Using ice-cold, 70% ethanol at 4 °C, cells were harvested and fixed for 12 h after that. Ethanol removal and cold PBS washing of the cells were done. Then incubated in 0.5 mL of PBS containing 1 mg/mL Ranse for 30 min at 37 °C. In the dark, the cells were stained with PI for 30 min. Flow cytometer was then used to detect contents of DNA^62^.

### 3.7. Annexin V-FITC apoptosis assay

For this study, annexin V-FITC/PI apoptosis detection kit was used; HCT-116 cells were stained with annexin V fluorescein isothiocyanate (FITC) and PI counter-stained. 1 × 10^5^ HCT-116 cells were 48 h incubated with compound **Vd** or **Ve**, trypsinised, washed with phosphate-buffered saline (PBS), stained in the dark at 37 °C for 15 min. Then, analysed with a cytometer of FACS calibre flow^63^.

### 3.8. Molecular docking

Molecular docking simulation studies were performed using molecular operating environment (MOE^®^) version 2015.10. The vascular endothelial growth factor receptor (VEGFR) (PDB 4ASD) was used as a receptor for the docking study and **Sorafenib** as a reference drug.

#### 3.8.1. Target compounds optimisation

Using the MOE program builder interface, the tested compounds **Vd**, **Ve**, **Vf**, **VIa**, **VIb**, and **VIc** were created into a 3D model. The target structures were checked by 2D depiction and formal charges on atoms, and then a conformational search was conducted for the target compounds. All conformers were subjected to energy minimisation done with MOE until an RMSD gradient of 0.01 kcal/mol and an RMS distance of 0.1 Å with MMFF94X were automatically measured as a force-field and the partial charges. The database of target compounds was then saved as MDB file for use in the calculations for molecular docking.

#### 3.8.2. Optimisation of VEGFR active site

The VEGFR has been prepared for docking experiments by adding hydrogen atoms and their standard geometry. The atom's connections and types were checked with automatic correction for any errors that existed. Selection of the receptor and its potential atoms has been fixed. MOE Alpha Site Finder used all default items to search for the active site in the receptor structure, and then dummy atoms were created from the alpha spheres obtained.

#### 3.8.3. Docking of the target compounds to the VEGFR active sites

Docking of the tested compounds' conformational database was performed using MOE-Dock software. To ensure a reasonable docking accuracy and to determine the effect of the water molecules, the co-crystallised ligand in the VEGFR (PDB 4ASD) was docked to its corresponding protein (in the absence and in the presence of water) and the RMSD values were determined between the co-crystallised ligand and docked pose. The success rates obtained were highly excellent where the active site of the VEGFR was calculated from the binding of co-crystallised ligand and saved as MOE file. The active site file of the VEGFR was then loaded, and the docking tool was used. The program specifications have been adjusted to the dummy atoms as docking site, triangle matcher as placement methodology, London dG as scoring methodology that have been adjusted to its default values. The MDB file of the ligands to be docked (**Sorafenib** and target compounds) was loaded, and calculations for docking were run automatically. The poses obtained were studied and the poses which had the best ligand–receptor interactions were selected and stored for calculating energy.

## 4. Conclusions

In summary, novel piperazine–chalcone hybrids and related pyrazoline analogues were synthesised and six of them were selected at a single dose concentration (10^−1^ M) by NCI (Bethesda, MD) to test their *in vitro* anticancer activity against full 60 lines of human cancer cells. VEGFR-2 enzyme inhibitory assay was performed to investigate the mechanism of anticancer activity of the tested compounds. While, all tested compounds demonstrate good inhibitory activity against VEGFR-2, the most active compounds were **Vd** and **Ve** that have been shown to be able to cause cell cycle arrest during the G2/M process and inducing apoptosis in HCT-116 cells. The present research has led to the discovery of new cytotoxic compounds that target the VEGFR-2. Furthermore, a molecular docking study of selected compounds was carried out and confirmed that compounds **Vd** and **Ve** exhibited a direct interaction with the VEGFR.

## Supplementary Material

Supplemental Material
