# Supplementary material for: Novel piperazine–chalcone hybrids and related pyrazoline analogues targeting VEGFR-2 kinase; design, synthesis, molecular docking studies, and anticancer evaluation
Source: J Enzyme Inhib Med Chem. 2020 Dec 21;36(1):307–18. doi: 10.1080/14756366.2020.1861606 (PMC7758046; doi:10.1080/14756366.2020.1861606)
Supplement: Supplemental Material [file IENZ_A_1861606_SM7084.pdf]

Supplementary Materials

Novel Piperazine-Chalcone Hybrids and Related Pyrazoline Analogs Targeting VEGFR-2 kinase; Design, Synthesis, Molecular Docking studies and Anticancer Evaluation

Marwa F. Ahmed[a,b*], Eman Y. Santali[a] and Radwan El-Haggar[b*]

[a]*Department of Pharmaceutical Chemistry, Faculty of Pharmacy, Taif University, Taif 21974, Kingdom of Saudi Arabia.*

[b]*Department of Pharmaceutical Chemistry, Faculty of Pharmacy, Helwan University, Cairo, 11795, Egypt.*

* **Corresponding authors**:

Dr. Radwan El-Haggar, **Email:** radwan_elhaggar@pharm.helwan.edu.eg

Dr. Marwa F. Ahmed, **Email:** marwafarag80@yahoo.com

Supplementary Materials

NCI data 3-14

Representative ^{1}H and ^{13}C NMR spectra for selected compounds 15-36

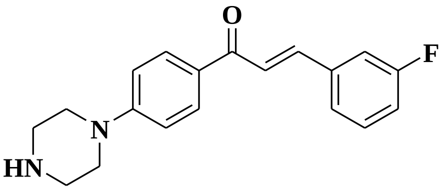

Vd

Developmental Therapeutics Program	NSC: D-799996 / 1	Conc: 1.00E-5 Molar	Test Date: Aug 14, 2017
One Dose Mean Graph	Experiment ID: 1708OS54		Report Date: Apr 18, 2020

Panel/Cell Line	Growth Percent
**Leukemia**	
CCRF-CEM	58.18
HL-60(TB)	60.38
K-562	16.55
MOLT-4	68.62
RPMI-8226	76.47
SR	55.72
**Non-Small Cell Lung Cancer**	
A549/ATCC	76.84
EKVX	95.54
HOP-62	80.17
HOP-92	58.41
NCI-H226	87.11
NCI-H23	77.30
NCI-H322M	75.26
NCI-H460	74.45
NCI-H522	71.57
**Colon Cancer**	
COLO 205	96.91
HCC-2998	94.99
HCT-116	42.08
HCT-15	73.49
HT29	30.44
KM12	77.75
SW-620	61.08
**CNS Cancer**	
SF-268	85.41
SF-295	101.78
SF-539	94.49
SNB-19	71.95
SNB-75	85.44
U251	72.39
**Melanoma**	
LOX IMVI	71.13
MALME-3M	94.15
M14	81.16
MDA-MB-435	94.27
SK-MEL-2	96.08
SK-MEL-28	109.03
SK-MEL-5	76.85
UACC-257	75.92
UACC-62	79.23
**Ovarian Cancer**	
IGROV1	67.97
OVCAR-3	93.74
OVCAR-4	80.53
OVCAR-5	94.72
OVCAR-8	67.16
NCI/ADR-RES	109.37
SK-OV-3	79.98
**Renal Cancer**	
786-0	85.24
A498	95.14
ACHN	84.70
CAKI-1	68.63
RXF 393	75.94
SN12C	68.16
TK-10	114.19
UO-31	76.58
**Prostate Cancer**	
PC-3	66.01
DU-145	77.70
**Breast Cancer**	
MCF7	51.72
MDA-MB-231/ATCC	73.60
HS 578T	79.41
BT-549	73.16
T-47D	87.54
MDA-MB-468	94.85
Mean	77.74
Delta	61.19
Range	97.64

Mean Growth Percent - Growth Percent

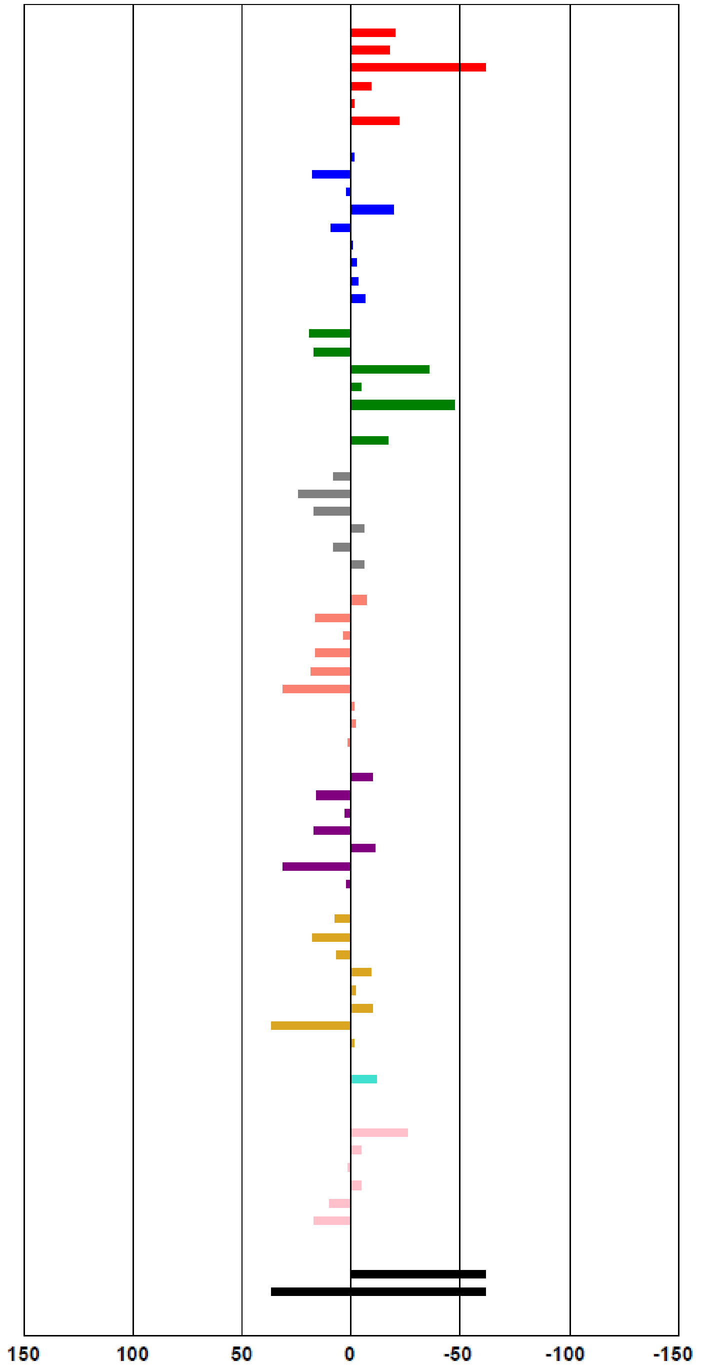

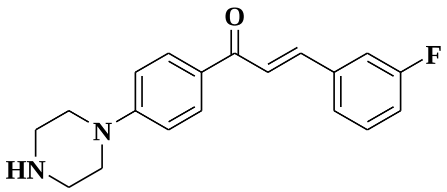

Vd

	A	B	C	D	E	F	G	H	I	J	K	L	M	N	O	P
1	NSC	EXPID	PLANDA	TESTSE	PREFIX	SAMPLE	DISCREI	CONC	CONCUI	CONCUI	PANELN	CELLNB	PANELN	CELLNA	GIPRCNT	
2	799996	1708OS5	Mon Aug	103	S	1	D	0.00001	M	Molar	7	3	Leukemi	CCRF-C	58.1814	
3	799996	1708OS5	Mon Aug	103	S	1	D	0.00001	M	Molar	7	8	Leukemi	HL-60(TE	60.3797	
4	799996	1708OS5	Mon Aug	103	S	1	D	0.00001	M	Molar	7	5	Leukemi	K-562	16.5503	
5	799996	1708OS5	Mon Aug	103	S	1	D	0.00001	M	Molar	7	6	Leukemi	MOLT-4	68.6169	
6	799996	1708OS5	Mon Aug	103	S	1	D	0.00001	M	Molar	7	10	Leukemi	RPMI-82	76.466	
7	799996	1708OS5	Mon Aug	103	S	1	D	0.00001	M	Molar	7	19	Leukemi	SR	55.7191	
8	799996	1708OS5	Mon Aug	103	S	1	D	0.00001	M	Molar	1	4	Non-Sm	A549/AT	76.8433	
9	799996	1708OS5	Mon Aug	103	S	1	D	0.00001	M	Molar	1	8	Non-Sm	EKVX	95.5423	
10	799996	1708OS5	Mon Aug	103	S	1	D	0.00001	M	Molar	1	26	Non-Sm	HOP-62	80.1676	
11	799996	1708OS5	Mon Aug	103	S	1	D	0.00001	M	Molar	1	29	Non-Sm	HOP-92	58.4131	
12	799996	1708OS5	Mon Aug	103	S	1	D	0.00001	M	Molar	1	13	Non-Sm	NCI-H22	87.1077	
13	799996	1708OS5	Mon Aug	103	S	1	D	0.00001	M	Molar	1	1	Non-Sm	NCI-H23	77.3021	
14	799996	1708OS5	Mon Aug	103	S	1	D	0.00001	M	Molar	1	17	Non-Sm	NCI-H32	75.2553	
15	799996	1708OS5	Mon Aug	103	S	1	D	0.00001	M	Molar	1	21	Non-Sm	NCI-H46	74.4469	
16	799996	1708OS5	Mon Aug	103	S	1	D	0.00001	M	Molar	1	3	Non-Sm	NCI-H52	71.5729	
17	799996	1708OS5	Mon Aug	103	S	1	D	0.00001	M	Molar	4	10	Colon C	COLO 20	96.9142	
18	799996	1708OS5	Mon Aug	103	S	1	D	0.00001	M	Molar	4	2	Colon C	HCC-29	94.9904	
19	799996	1708OS5	Mon Aug	103	S	1	D	0.00001	M	Molar	4	3	Colon C	HCT-116	42.0827	
20	799996	1708OS5	Mon Aug	103	S	1	D	0.00001	M	Molar	4	15	Colon C	HCT-15	73.4915	
21	799996	1708OS5	Mon Aug	103	S	1	D	0.00001	M	Molar	4	1	Colon C	HT29	30.4422	
22	799996	1708OS5	Mon Aug	103	S	1	D	0.00001	M	Molar	4	17	Colon C	KM12	77.7476	
23	799996	1708OS5	Mon Aug	103	S	1	D	0.00001	M	Molar	4	9	Colon C	SW-620	61.0752	
24	799996	1708OS5	Mon Aug	103	S	1	D	0.00001	M	Molar	12	14	CNS Car	SF-268	85.4138	
25	799996	1708OS5	Mon Aug	103	S	1	D	0.00001	M	Molar	12	15	CNS Car	SF-295	101.78	
26	799996	1708OS5	Mon Aug	103	S	1	D	0.00001	M	Molar	12	16	CNS Car	SF-539	94.4948	
27	799996	1708OS5	Mon Aug	103	S	1	D	0.00001	M	Molar	12	2	CNS Car	SNB-19	71.9539	
28	799996	1708OS5	Mon Aug	103	S	1	D	0.00001	M	Molar	12	5	CNS Car	SNB-75	85.4389	
29	799996	1708OS5	Mon Aug	103	S	1	D	0.00001	M	Molar	12	9	CNS Car	U251	72.3859	
30	799996	1708OS5	Mon Aug	103	S	1	D	0.00001	M	Molar	10	1	Melanon	LOX IMV	71.1303	
31	799996	1708OS5	Mon Aug	103	S	1	D	0.00001	M	Molar	10	2	Melanon	MALME	94.1479	
32	799996	1708OS5	Mon Aug	103	S	1	D	0.00001	M	Molar	10	14	Melanon	M14	81.162	
33	799996	1708OS5	Mon Aug	103	S	1	D	0.00001	M	Molar	5	11	Melanon	MDA-MI	94.2726	
34	799996	1708OS5	Mon Aug	103	S	1	D	0.00001	M	Molar	10	5	Melanon	SK-MEL-	96.0771	
35	799996	1708OS5	Mon Aug	103	S	1	D	0.00001	M	Molar	10	8	Melanon	SK-MEL-	109.026	
36	799996	1708OS5	Mon Aug	103	S	1	D	0.00001	M	Molar	10	7	Melanon	SK-MEL-	76.8535	
37	799996	1708OS5	Mon Aug	103	S	1	D	0.00001	M	Molar	10	21	Melanon	UACC-2	75.9151	
38	799996	1708OS5	Mon Aug	103	S	1	D	0.00001	M	Molar	10	20	Melanon	UACC-6	79.2331	
39	799996	1708OS5	Mon Aug	103	S	1	D	0.00001	M	Molar	6	10	Ovarian (	IGROV1	67.9669	
40	799996	1708OS5	Mon Aug	103	S	1	D	0.00001	M	Molar	6	1	Ovarian (	OVCAR-	93.7416	
41	799996	1708OS5	Mon Aug	103	S	1	D	0.00001	M	Molar	6	2	Ovarian (	OVCAR-	80.5332	
42	799996	1708OS5	Mon Aug	103	S	1	D	0.00001	M	Molar	6	3	Ovarian (	OVCAR-	94.7192	
43	799996	1708OS5	Mon Aug	103	S	1	D	0.00001	M	Molar	6	5	Ovarian (	OVCAR-	67.1646	
44	799996	1708OS5	Mon Aug	103	S	1	D	0.00001	M	Molar	5	2	Ovarian (	NCI/ADF	109.366	
45	799996	1708OS5	Mon Aug	103	S	1	D	0.00001	M	Molar	6	11	Ovarian (	SK-OV-3	79.9765	
46	799996	1708OS5	Mon Aug	103	S	1	D	0.00001	M	Molar	9	18	Renal Ca	786-0	85.2404	
47	799996	1708OS5	Mon Aug	103	S	1	D	0.00001	M	Molar	9	13	Renal Ca	A498	95.1442	
48	799996	1708OS5	Mon Aug	103	S	1	D	0.00001	M	Molar	9	23	Renal Ca	ACHN	84.6973	
49	799996	1708OS5	Mon Aug	103	S	1	D	0.00001	M	Molar	9	15	Renal Ca	CAKI-1	68.6283	
50	799996	1708OS5	Mon Aug	103	S	1	D	0.00001	M	Molar	9	16	Renal Ca	RXF 393	75.9381	
51	799996	1708OS5	Mon Aug	103	S	1	D	0.00001	M	Molar	9	8	Renal Ca	SN12C	68.1601	
52	799996	1708OS5	Mon Aug	103	S	1	D	0.00001	M	Molar	9	24	Renal Ca	TK-10	114.193	
53	799996	1708OS5	Mon Aug	103	S	1	D	0.00001	M	Molar	9	4	Renal Ca	UO-31	76.5835	
54	799996	1708OS5	Mon Aug	103	S	1	D	0.00001	M	Molar	11	1	Prostate	PC-3	66.0123	
55	799996	1708OS5	Mon Aug	103	S	1	D	0.00001	M	Molar	11	3	Prostate	DU-145	77.6958	
56	799996	1708OS5	Mon Aug	103	S	1	D	0.00001	M	Molar	5	1	Breast C	MCF7	51.7161	
57	799996	1708OS5	Mon Aug	103	S	1	D	0.00001	M	Molar	5	5	Breast C	MDA-MI	73.5986	
58	799996	1708OS5	Mon Aug	103	S	1	D	0.00001	M	Molar	5	6	Breast C	HS 578T	79.4085	
59	799996	1708OS5	Mon Aug	103	S	1	D	0.00001	M	Molar	5	13	Breast C	BT-549	73.1605	
60	799996	1708OS5	Mon Aug	103	S	1	D	0.00001	M	Molar	5	14	Breast C	T-47D	87.5421	
61	799996	1708OS5	Mon Aug	103	S	1	D	0.00001	M	Molar	5	18	Breast C	MDA-MI	94.8477	
62																
63																
64																

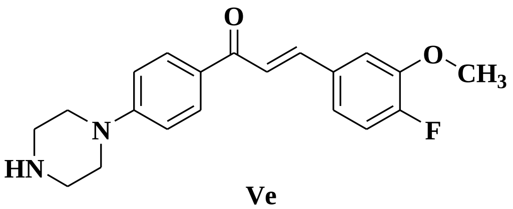

Ve

Developmental Therapeutics Program	NSC: D-799997 / 1	Conc: 1.00E-5 Molar	Test Date: Aug 14, 2017
One Dose Mean Graph	Experiment ID: 1708OS54		Report Date: Apr 18, 2020

Panel/Cell Line	Growth Percent
**Leukemia**	
CCRF-CEM	22.15
HL-60(TB)	28.91
K-562	12.37
MOLT-4	80.75
RPMI-8226	49.59
SR	46.22
**Non-Small Cell Lung Cancer**	
A549/ATCC	67.25
EKVX	97.12
HOP-62	101.42
HOP-92	55.00
NCI-H226	86.54
NCI-H23	92.63
NCI-H322M	73.01
NCI-H460	97.07
NCI-H522	61.19
**Colon Cancer**	
COLO 205	83.00
HCC-2998	100.21
HCT-116	27.43
HCT-15	43.16
HT29	49.97
KM12	60.47
SW-620	50.69
**CNS Cancer**	
SF-268	94.03
SF-295	95.92
SF-539	95.87
SNB-19	59.87
SNB-75	89.05
U251	67.21
**Melanoma**	
LOX IMVI	49.01
MALME-3M	112.40
M14	78.43
MDA-MB-435	80.14
SK-MEL-2	85.44
SK-MEL-28	106.02
SK-MEL-5	79.33
UACC-257	76.08
UACC-62	80.13
**Ovarian Cancer**	
IGROV1	59.94
OVCAR-3	70.18
OVCAR-4	68.87
OVCAR-5	121.73
OVCAR-8	57.23
NCI/ADR-RES	79.31
SK-OV-3	87.98
**Renal Cancer**	
786-0	87.22
A498	100.79
ACHN	68.11
CAKI-1	79.37
RXF 393	101.92
SN12C	57.67
TK-10	101.24
UO-31	58.35
**Prostate Cancer**	
PC-3	51.40
DU-145	105.61
**Breast Cancer**	
MCF7	34.68
MDA-MB-231/ATCC	94.50
HS 578T	91.26
BT-549	66.42
T-47D	74.40
MDA-MB-468	46.48
Mean	73.33
Delta	60.96
Range	109.36

Mean Growth Percent - Growth Percent

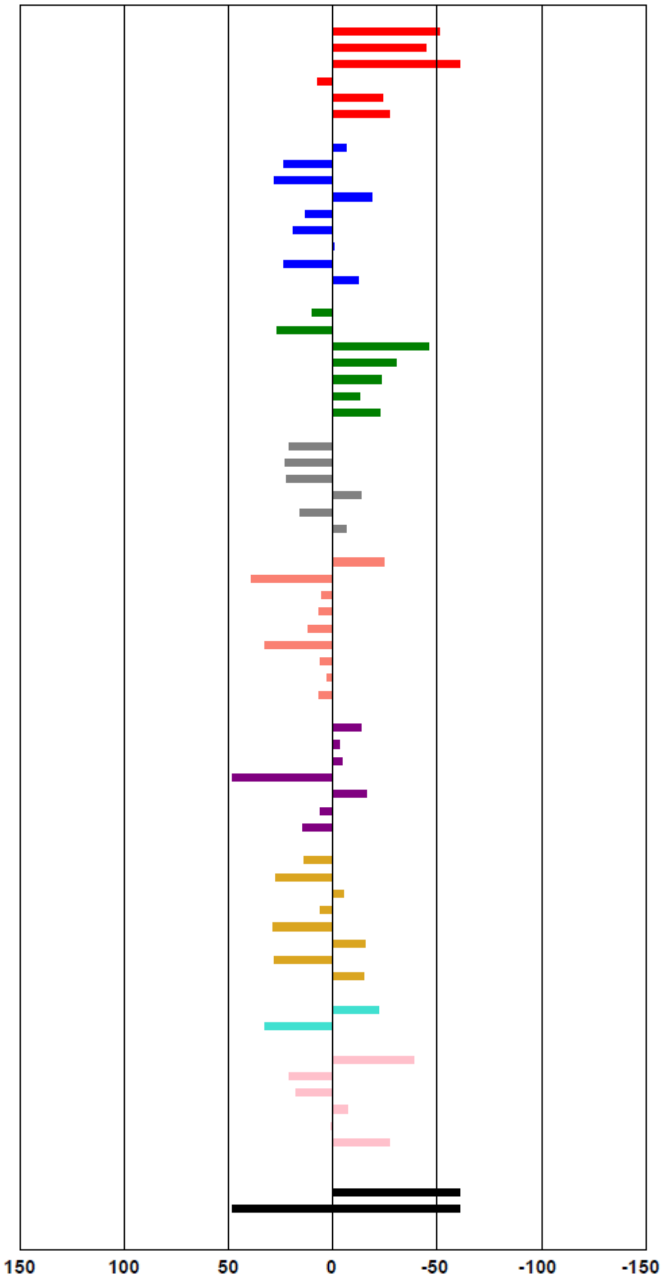

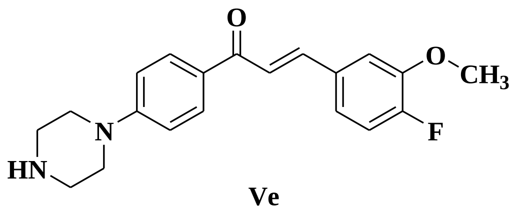

Ve

A1 *fx* NSC

	A	B	C	D	E	F	G	H	I	J	K	L	M	N	O
1	NSC	EXPID	PLANDA	TESTSE	PREFIX	SAMPLE	DISCREI	CONC	CONCUI	CONCUI	PANELN	CELLNB	PANELN	CELLNA	GIPRCNT
2	799997	1708OS5	Mon Aug	113	S	1	D	0.00001	M	Molar	7	3	Leukemi	CCRF-C	22.1479
3	799997	1708OS5	Mon Aug	113	S	1	D	0.00001	M	Molar	7	8	Leukemi	HL-60(TE	28.9061
4	799997	1708OS5	Mon Aug	113	S	1	D	0.00001	M	Molar	7	5	Leukemi	K-562	12.373
5	799997	1708OS5	Mon Aug	113	S	1	D	0.00001	M	Molar	7	6	Leukemi	MOLT-4	80.7468
6	799997	1708OS5	Mon Aug	113	S	1	D	0.00001	M	Molar	7	10	Leukemi	RPMI-82	49.5889
7	799997	1708OS5	Mon Aug	113	S	1	D	0.00001	M	Molar	7	19	Leukemi	SR	46.2222
8	799997	1708OS5	Mon Aug	113	S	1	D	0.00001	M	Molar	1	4	Non-Sm	A549/AT	67.25
9	799997	1708OS5	Mon Aug	113	S	1	D	0.00001	M	Molar	1	8	Non-Sm	EKVX	97.1164
10	799997	1708OS5	Mon Aug	113	S	1	D	0.00001	M	Molar	1	26	Non-Sm	HOP-62	101.422
11	799997	1708OS5	Mon Aug	113	S	1	D	0.00001	M	Molar	1	29	Non-Sm	HOP-92	54.9957
12	799997	1708OS5	Mon Aug	113	S	1	D	0.00001	M	Molar	1	13	Non-Sm	NCI-H22	86.5426
13	799997	1708OS5	Mon Aug	113	S	1	D	0.00001	M	Molar	1	1	Non-Sm	NCI-H23	92.629
14	799997	1708OS5	Mon Aug	113	S	1	D	0.00001	M	Molar	1	17	Non-Sm	NCI-H32	73.0061
15	799997	1708OS5	Mon Aug	113	S	1	D	0.00001	M	Molar	1	21	Non-Sm	NCI-H46	97.0664
16	799997	1708OS5	Mon Aug	113	S	1	D	0.00001	M	Molar	1	3	Non-Sm	NCI-H52	61.1934
17	799997	1708OS5	Mon Aug	113	S	1	D	0.00001	M	Molar	4	10	Colon C	COLO 20	82.9973
18	799997	1708OS5	Mon Aug	113	S	1	D	0.00001	M	Molar	4	2	Colon C	HCC-29	100.206
19	799997	1708OS5	Mon Aug	113	S	1	D	0.00001	M	Molar	4	3	Colon C	HCT-116	27.4341
20	799997	1708OS5	Mon Aug	113	S	1	D	0.00001	M	Molar	4	15	Colon C	HCT-15	43.1587
21	799997	1708OS5	Mon Aug	113	S	1	D	0.00001	M	Molar	4	1	Colon C	HT29	49.9667
22	799997	1708OS5	Mon Aug	113	S	1	D	0.00001	M	Molar	4	17	Colon C	KM12	60.4651
23	799997	1708OS5	Mon Aug	113	S	1	D	0.00001	M	Molar	4	9	Colon C	SW-620	50.6927
24	799997	1708OS5	Mon Aug	113	S	1	D	0.00001	M	Molar	12	14	CNS Car	SF-268	94.031
25	799997	1708OS5	Mon Aug	113	S	1	D	0.00001	M	Molar	12	15	CNS Car	SF-295	95.9248
26	799997	1708OS5	Mon Aug	113	S	1	D	0.00001	M	Molar	12	16	CNS Car	SF-539	95.871
27	799997	1708OS5	Mon Aug	113	S	1	D	0.00001	M	Molar	12	2	CNS Car	SNB-19	59.8714
28	799997	1708OS5	Mon Aug	113	S	1	D	0.00001	M	Molar	12	5	CNS Car	SNB-75	89.0457
29	799997	1708OS5	Mon Aug	113	S	1	D	0.00001	M	Molar	12	9	CNS Car	U251	67.2089
30	799997	1708OS5	Mon Aug	113	S	1	D	0.00001	M	Molar	10	1	Melanon	LOX IMV	49.0062
31	799997	1708OS5	Mon Aug	113	S	1	D	0.00001	M	Molar	10	2	Melanon	MALME	112.398
32	799997	1708OS5	Mon Aug	113	S	1	D	0.00001	M	Molar	10	14	Melanon	M14	78.4271
33	799997	1708OS5	Mon Aug	113	S	1	D	0.00001	M	Molar	5	11	Melanon	MDA-MI	80.1436
34	799997	1708OS5	Mon Aug	113	S	1	D	0.00001	M	Molar	10	5	Melanon	SK-MEL-	85.4361
35	799997	1708OS5	Mon Aug	113	S	1	D	0.00001	M	Molar	10	8	Melanon	SK-MEL-	106.017
36	799997	1708OS5	Mon Aug	113	S	1	D	0.00001	M	Molar	10	7	Melanon	SK-MEL-	79.325
37	799997	1708OS5	Mon Aug	113	S	1	D	0.00001	M	Molar	10	21	Melanon	UACC-2	76.077
38	799997	1708OS5	Mon Aug	113	S	1	D	0.00001	M	Molar	10	20	Melanon	UACC-6	80.1276
39	799997	1708OS5	Mon Aug	113	S	1	D	0.00001	M	Molar	6	10	Ovarian (	IGROV1	59.9407
40	799997	1708OS5	Mon Aug	113	S	1	D	0.00001	M	Molar	6	1	Ovarian (	OVCAR-	70.1833
41	799997	1708OS5	Mon Aug	113	S	1	D	0.00001	M	Molar	6	2	Ovarian (	OVCAR-	68.8713
42	799997	1708OS5	Mon Aug	113	S	1	D	0.00001	M	Molar	6	3	Ovarian (	OVCAR-	121.728
43	799997	1708OS5	Mon Aug	113	S	1	D	0.00001	M	Molar	6	5	Ovarian (	OVCAR-	57.2274
44	799997	1708OS5	Mon Aug	113	S	1	D	0.00001	M	Molar	5	2	Ovarian (	NCI/ADF	79.3056
45	799997	1708OS5	Mon Aug	113	S	1	D	0.00001	M	Molar	6	11	Ovarian (	SK-OV-3	87.9844
46	799997	1708OS5	Mon Aug	113	S	1	D	0.00001	M	Molar	9	18	Renal Ca	786-0	87.2188
47	799997	1708OS5	Mon Aug	113	S	1	D	0.00001	M	Molar	9	13	Renal Ca	A498	100.791
48	799997	1708OS5	Mon Aug	113	S	1	D	0.00001	M	Molar	9	23	Renal Ca	ACHN	68.1073
49	799997	1708OS5	Mon Aug	113	S	1	D	0.00001	M	Molar	9	15	Renal Ca	CAKI-1	79.3708
50	799997	1708OS5	Mon Aug	113	S	1	D	0.00001	M	Molar	9	16	Renal Ca	RXF 393	101.925
51	799997	1708OS5	Mon Aug	113	S	1	D	0.00001	M	Molar	9	8	Renal Ca	SN12C	57.6682
52	799997	1708OS5	Mon Aug	113	S	1	D	0.00001	M	Molar	9	24	Renal Ca	TK-10	101.241
53	799997	1708OS5	Mon Aug	113	S	1	D	0.00001	M	Molar	9	4	Renal Ca	UO-31	58.3531
54	799997	1708OS5	Mon Aug	113	S	1	D	0.00001	M	Molar	11	1	Prostate	PC-3	51.3971
55	799997	1708OS5	Mon Aug	113	S	1	D	0.00001	M	Molar	11	3	Prostate	DU-145	105.611
56	799997	1708OS5	Mon Aug	113	S	1	D	0.00001	M	Molar	5	1	Breast C	MCF7	34.6834
57	799997	1708OS5	Mon Aug	113	S	1	D	0.00001	M	Molar	5	5	Breast C	MDA-MI	94.4986
58	799997	1708OS5	Mon Aug	113	S	1	D	0.00001	M	Molar	5	6	Breast C	HS 578T	91.2629
59	799997	1708OS5	Mon Aug	113	S	1	D	0.00001	M	Molar	5	13	Breast C	BT-549	66.4165
60	799997	1708OS5	Mon Aug	113	S	1	D	0.00001	M	Molar	5	14	Breast C	T-47D	74.4048
61	799997	1708OS5	Mon Aug	113	S	1	D	0.00001	M	Molar	5	18	Breast C	MDA-MI	46.475

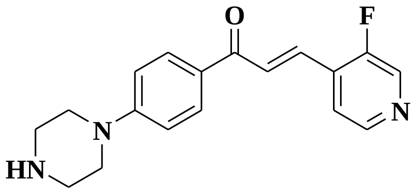

Vf

Developmental Therapeutics Program One Dose Mean Graph	NSC: D-799998 / 1	Conc: 1.00E-5 Molar	Test Date: Aug 14, 2017
	Experiment ID: 1708OS54		Report Date: Apr 18, 2020

Panel/Cell Line	Growth Percent
**Leukemia**	
CCRF-CEM	78.16
HL-60(TB)	79.28
K-562	85.84
MOLT-4	91.63
RPMI-8226	97.33
SR	92.99
**Non-Small Cell Lung Cancer**	
A549/ATCC	78.75
EKVX	99.11
HOP-62	102.01
HOP-92	82.34
NCI-H226	99.71
NCI-H23	102.52
NCI-H322M	96.26
NCI-H460	106.01
NCI-H522	78.82
**Colon Cancer**	
COLO 205	111.89
HCC-2998	100.88
HCT-116	73.87
HCT-15	111.39
HT29	86.42
KM12	105.34
SW-620	105.59
**CNS Cancer**	
SF-268	100.92
SF-295	101.95
SF-539	101.29
SNB-19	96.07
SNB-75	89.08
U251	95.03
**Melanoma**	
LOX IMVI	103.22
MALME-3M	99.98
M14	95.53
MDA-MB-435	102.37
SK-MEL-2	91.63
SK-MEL-28	115.56
SK-MEL-5	101.11
UACC-257	70.86
UACC-62	92.01
**Ovarian Cancer**	
IGROV1	97.17
OVCAR-3	106.22
OVCAR-4	110.55
OVCAR-5	111.84
OVCAR-8	95.35
NCI/ADR-RES	107.53
SK-OV-3	96.68
**Renal Cancer**	
786-0	103.38
A498	97.22
ACHN	113.06
CAKI-1	86.18
RXF 393	97.78
SN12C	99.00
TK-10	95.83
UO-31	89.78
**Prostate Cancer**	
PC-3	73.56
DU-145	108.14
**Breast Cancer**	
MCF7	99.38
MDA-MB-231/ATCC	98.76
HS 578T	99.19
BT-549	90.81
T-47D	118.12
MDA-MB-468	100.32
Mean	96.98
Delta	26.12
Range	47.26

Mean Growth Percent - Growth Percent

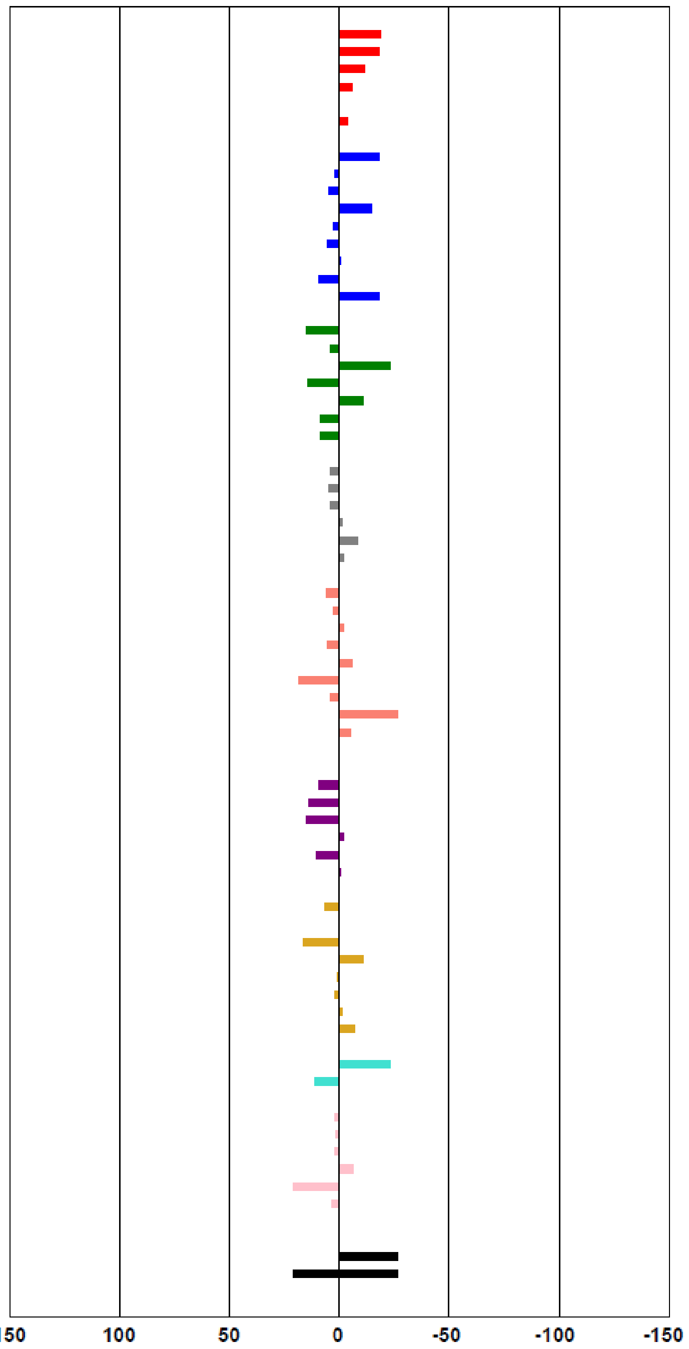

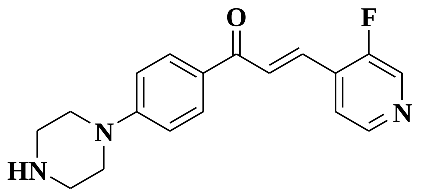

Vf

A1 | NSC

	A	B	C	D	E	F	G	H	I	J	K	L	M	N	O
1	NSC	EXPID	PLANDA	TESTSE	PREFIX	SAMPLE	DISCREI	CONC	CONCUI	CONCUI	PANELN	CELLNB	PANELN	CELLNA	GIPRCNT
2	799998	1708OS5	Mon Aug	123	S	1	D	0.00001	M	Molar	7	3	Leukemi	CCRF-C	78.1606
3	799998	1708OS5	Mon Aug	123	S	1	D	0.00001	M	Molar	7	8	Leukemi	HL-60(TE	79.2846
4	799998	1708OS5	Mon Aug	123	S	1	D	0.00001	M	Molar	7	5	Leukemi	K-562	85.836
5	799998	1708OS5	Mon Aug	123	S	1	D	0.00001	M	Molar	7	6	Leukemi	MOLT-4	91.6312
6	799998	1708OS5	Mon Aug	123	S	1	D	0.00001	M	Molar	7	10	Leukemi	RPMI-82	97.3264
7	799998	1708OS5	Mon Aug	123	S	1	D	0.00001	M	Molar	7	19	Leukemi	SR	92.9947
8	799998	1708OS5	Mon Aug	123	S	1	D	0.00001	M	Molar	1	4	Non-Sm	A549/AT	78.7503
9	799998	1708OS5	Mon Aug	123	S	1	D	0.00001	M	Molar	1	8	Non-Sm	EKVX	99.1057
10	799998	1708OS5	Mon Aug	123	S	1	D	0.00001	M	Molar	1	26	Non-Sm	HOP-62	102.011
11	799998	1708OS5	Mon Aug	123	S	1	D	0.00001	M	Molar	1	29	Non-Sm	HOP-92	82.3412
12	799998	1708OS5	Mon Aug	123	S	1	D	0.00001	M	Molar	1	13	Non-Sm	NCI-H22	99.713
13	799998	1708OS5	Mon Aug	123	S	1	D	0.00001	M	Molar	1	1	Non-Sm	NCI-H23	102.517
14	799998	1708OS5	Mon Aug	123	S	1	D	0.00001	M	Molar	1	17	Non-Sm	NCI-H32	96.2614
15	799998	1708OS5	Mon Aug	123	S	1	D	0.00001	M	Molar	1	21	Non-Sm	NCI-H46	106.014
16	799998	1708OS5	Mon Aug	123	S	1	D	0.00001	M	Molar	1	3	Non-Sm	NCI-H52	78.8167
17	799998	1708OS5	Mon Aug	123	S	1	D	0.00001	M	Molar	4	10	Colon C	COLO 20	111.888
18	799998	1708OS5	Mon Aug	123	S	1	D	0.00001	M	Molar	4	2	Colon C	HCC-29	100.88
19	799998	1708OS5	Mon Aug	123	S	1	D	0.00001	M	Molar	4	3	Colon C	HCT-116	73.8715
20	799998	1708OS5	Mon Aug	123	S	1	D	0.00001	M	Molar	4	15	Colon C	HCT-15	111.394
21	799998	1708OS5	Mon Aug	123	S	1	D	0.00001	M	Molar	4	1	Colon C	HT29	86.4204
22	799998	1708OS5	Mon Aug	123	S	1	D	0.00001	M	Molar	4	17	Colon C	KM12	105.342
23	799998	1708OS5	Mon Aug	123	S	1	D	0.00001	M	Molar	4	9	Colon C	SW-620	105.588
24	799998	1708OS5	Mon Aug	123	S	1	D	0.00001	M	Molar	12	14	CNS Car	SF-268	100.919
25	799998	1708OS5	Mon Aug	123	S	1	D	0.00001	M	Molar	12	15	CNS Car	SF-295	101.945
26	799998	1708OS5	Mon Aug	123	S	1	D	0.00001	M	Molar	12	16	CNS Car	SF-539	101.294
27	799998	1708OS5	Mon Aug	123	S	1	D	0.00001	M	Molar	12	2	CNS Car	SNB-19	96.0684
28	799998	1708OS5	Mon Aug	123	S	1	D	0.00001	M	Molar	12	5	CNS Car	SNB-75	89.0759
29	799998	1708OS5	Mon Aug	123	S	1	D	0.00001	M	Molar	12	9	CNS Car	U251	95.0339
30	799998	1708OS5	Mon Aug	123	S	1	D	0.00001	M	Molar	10	1	Melanon	LOX IMV	103.218
31	799998	1708OS5	Mon Aug	123	S	1	D	0.00001	M	Molar	10	2	Melanon	MALME	99.98
32	799998	1708OS5	Mon Aug	123	S	1	D	0.00001	M	Molar	10	14	Melanon	M14	95.5348
33	799998	1708OS5	Mon Aug	123	S	1	D	0.00001	M	Molar	5	11	Melanon	MDA-MI	102.373
34	799998	1708OS5	Mon Aug	123	S	1	D	0.00001	M	Molar	10	5	Melanon	SK-MEL-	91.6318
35	799998	1708OS5	Mon Aug	123	S	1	D	0.00001	M	Molar	10	8	Melanon	SK-MEL-	115.561
36	799998	1708OS5	Mon Aug	123	S	1	D	0.00001	M	Molar	10	7	Melanon	SK-MEL-	101.113
37	799998	1708OS5	Mon Aug	123	S	1	D	0.00001	M	Molar	10	21	Melanon	UACC-2	70.8639
38	799998	1708OS5	Mon Aug	123	S	1	D	0.00001	M	Molar	10	20	Melanon	UACC-6	92.014
39	799998	1708OS5	Mon Aug	123	S	1	D	0.00001	M	Molar	6	10	Ovarian (	IGROV1	97.1688
40	799998	1708OS5	Mon Aug	123	S	1	D	0.00001	M	Molar	6	1	Ovarian (	OVCAR-	106.217
41	799998	1708OS5	Mon Aug	123	S	1	D	0.00001	M	Molar	6	2	Ovarian (	OVCAR-	110.546
42	799998	1708OS5	Mon Aug	123	S	1	D	0.00001	M	Molar	6	3	Ovarian (	OVCAR-	111.843
43	799998	1708OS5	Mon Aug	123	S	1	D	0.00001	M	Molar	6	5	Ovarian (	OVCAR-	95.3451
44	799998	1708OS5	Mon Aug	123	S	1	D	0.00001	M	Molar	5	2	Ovarian (	NCI/ADF	107.526
45	799998	1708OS5	Mon Aug	123	S	1	D	0.00001	M	Molar	6	11	Ovarian (	SK-OV-3	96.685
46	799998	1708OS5	Mon Aug	123	S	1	D	0.00001	M	Molar	9	18	Renal Ca	786-0	103.378
47	799998	1708OS5	Mon Aug	123	S	1	D	0.00001	M	Molar	9	13	Renal Ca	A498	97.2198
48	799998	1708OS5	Mon Aug	123	S	1	D	0.00001	M	Molar	9	23	Renal Ca	ACHN	113.064
49	799998	1708OS5	Mon Aug	123	S	1	D	0.00001	M	Molar	9	15	Renal Ca	CAKI-1	86.1807
50	799998	1708OS5	Mon Aug	123	S	1	D	0.00001	M	Molar	9	16	Renal Ca	RXF 393	97.7834
51	799998	1708OS5	Mon Aug	123	S	1	D	0.00001	M	Molar	9	8	Renal Ca	SN12C	99.0019
52	799998	1708OS5	Mon Aug	123	S	1	D	0.00001	M	Molar	9	24	Renal Ca	TK-10	95.834
53	799998	1708OS5	Mon Aug	123	S	1	D	0.00001	M	Molar	9	4	Renal Ca	UO-31	89.7822
54	799998	1708OS5	Mon Aug	123	S	1	D	0.00001	M	Molar	11	1	Prostate	PC-3	73.5632
55	799998	1708OS5	Mon Aug	123	S	1	D	0.00001	M	Molar	11	3	Prostate	DU-145	108.144
56	799998	1708OS5	Mon Aug	123	S	1	D	0.00001	M	Molar	5	1	Breast C	MCF7	99.378
57	799998	1708OS5	Mon Aug	123	S	1	D	0.00001	M	Molar	5	5	Breast C	MDA-MI	98.7594
58	799998	1708OS5	Mon Aug	123	S	1	D	0.00001	M	Molar	5	6	Breast C	HS 578T	99.1854
59	799998	1708OS5	Mon Aug	123	S	1	D	0.00001	M	Molar	5	13	Breast C	BT-549	90.8091
60	799998	1708OS5	Mon Aug	123	S	1	D	0.00001	M	Molar	5	14	Breast C	T-47D	118.118
61	799998	1708OS5	Mon Aug	123	S	1	D	0.00001	M	Molar	5	18	Breast C	MDA-MI	100.323
62															
63															

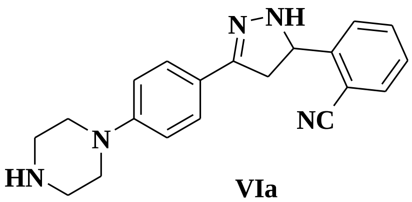
N
NH
NC
HN
N

VIa

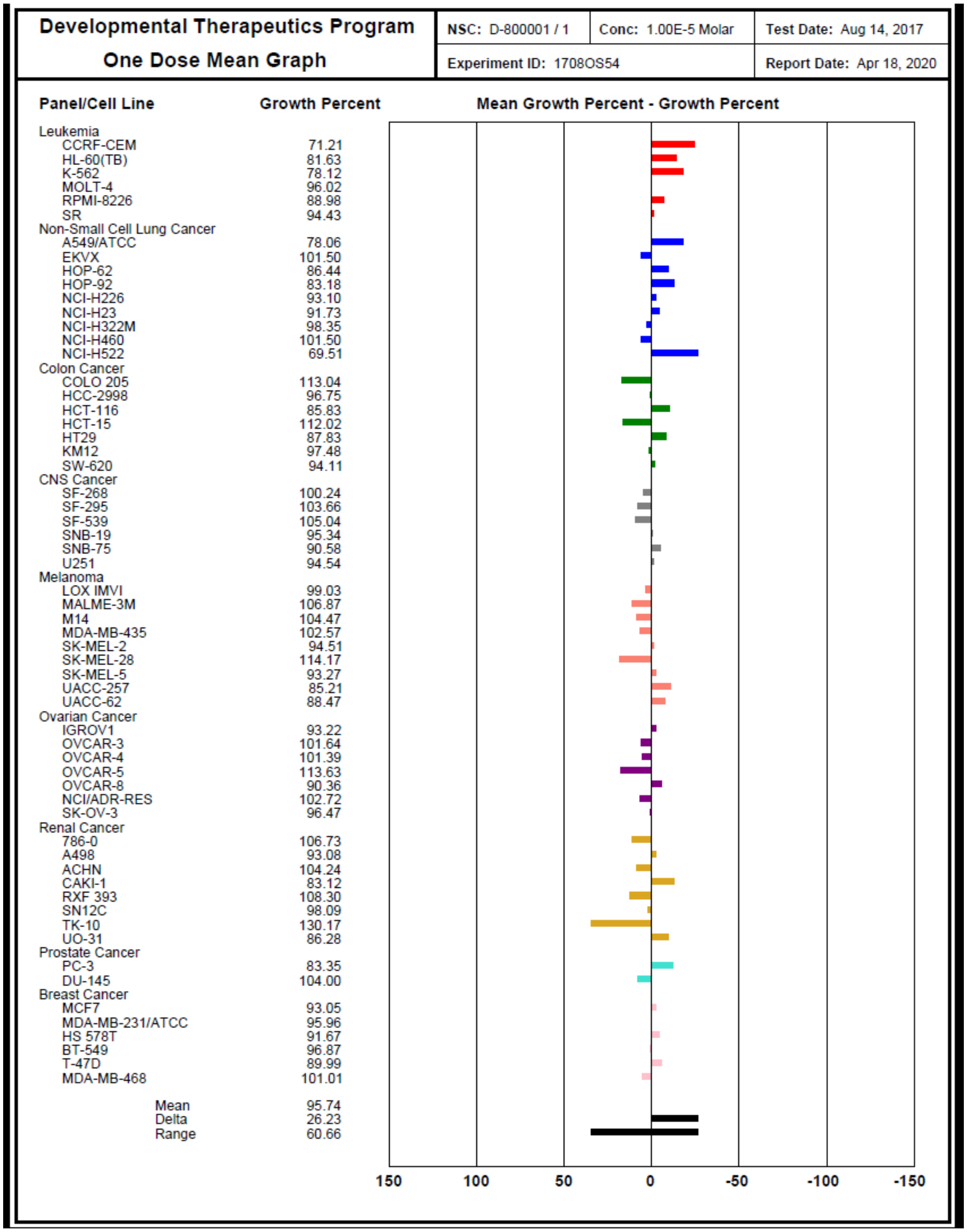
Developmental Therapeutics Program
One Dose Mean Graph
NSC: D-800001 / 1
Conc: 1.00E-5 Molar
Test Date: Aug 14, 2017
Experiment ID: 1708OS54
Report Date: Apr 18, 2020
Panel/Cell Line
Growth Percent
Mean Growth Percent - Growth Percent
Leukemia
CCRF-CEM 71.21
HL-60(TB) 81.63
K-562 78.12
MOLT-4 96.02
RPMI-8226 88.98
SR 94.43
Non-Small Cell Lung Cancer
A549/ATCC 78.06
EKVX 101.50
HOP-62 86.44
HOP-92 83.18
NCI-H226 93.10
NCI-H23 91.73
NCI-H322M 98.35
NCI-H460 101.50
NCI-H522 69.51
Colon Cancer
COLO 205 113.04
HCC-2998 96.75
HCT-116 85.83
HCT-15 112.02
HT29 87.83
KM12 97.48
SW-620 94.11
CNS Cancer
SF-268 100.24
SF-295 103.66
SF-539 105.04
SNB-19 95.34
SNB-75 90.58
U251 94.54
Melanoma
LOX IMVI 99.03
MALME-3M 106.87
M14 104.47
MDA-MB-435 102.57
SK-MEL-2 94.51
SK-MEL-28 114.17
SK-MEL-5 93.27
UACC-257 85.21
UACC-62 88.47
Ovarian Cancer
IGROV1 93.22
OVCAR-3 101.64
OVCAR-4 101.39
OVCAR-5 113.63
OVCAR-8 90.36
NCI/ADR-RES 102.72
SK-OV-3 96.47
Renal Cancer
786-0 106.73
A498 93.08
ACHN 104.24
CAKI-1 83.12
RXF 393 108.30
SN12C 98.09
TK-10 130.17
UO-31 86.28
Prostate Cancer
PC-3 83.35
DU-145 104.00
Breast Cancer
MCF7 93.05
MDA-MB-231/ATCC 95.96
HS 578T 91.67
BT-549 96.87
T-47D 89.99
MDA-MB-468 101.01
Mean 95.74
Delta 26.23
Range 60.66
150
100
50
0
-50
-100
-150

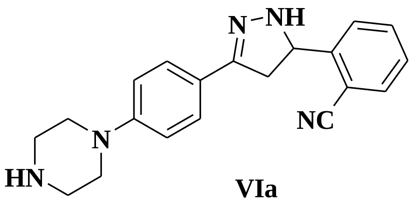

VIa

A1 | NSC

	A	B	C	D	E	F	G	H	I	J	K	L	M	N	O	P
1	NSC	EXPID	PLANDA	TESTSE	PREFIX	SAMPLE	DISCREI	CONC	CONCUI	CONCUI	PANELN	CELLNB	PANELN	CELLNA	GIPRCNT	
2	800001	1708OS5	Mon Aug	153	S	1	D	0.00001	M	Molar	7	3	Leukemi	CCRF-C	71.2122	
3	800001	1708OS5	Mon Aug	153	S	1	D	0.00001	M	Molar	7	8	Leukemi	HL-60(TE	81.6321	
4	800001	1708OS5	Mon Aug	153	S	1	D	0.00001	M	Molar	7	5	Leukemi	K-562	78.1181	
5	800001	1708OS5	Mon Aug	153	S	1	D	0.00001	M	Molar	7	6	Leukemi	MOLT-4	96.0205	
6	800001	1708OS5	Mon Aug	153	S	1	D	0.00001	M	Molar	7	10	Leukemi	RPMI-82	88.9759	
7	800001	1708OS5	Mon Aug	153	S	1	D	0.00001	M	Molar	7	19	Leukemi	SR	94.4288	
8	800001	1708OS5	Mon Aug	153	S	1	D	0.00001	M	Molar	1	4	Non-Sm	A549/AT	78.0557	
9	800001	1708OS5	Mon Aug	153	S	1	D	0.00001	M	Molar	1	8	Non-Sm	EKVX	101.498	
10	800001	1708OS5	Mon Aug	153	S	1	D	0.00001	M	Molar	1	26	Non-Sm	HOP-62	86.4428	
11	800001	1708OS5	Mon Aug	153	S	1	D	0.00001	M	Molar	1	29	Non-Sm	HOP-92	83.1833	
12	800001	1708OS5	Mon Aug	153	S	1	D	0.00001	M	Molar	1	13	Non-Sm	NCI-H22	93.0984	
13	800001	1708OS5	Mon Aug	153	S	1	D	0.00001	M	Molar	1	1	Non-Sm	NCI-H23	91.7293	
14	800001	1708OS5	Mon Aug	153	S	1	D	0.00001	M	Molar	1	17	Non-Sm	NCI-H32	98.3467	
15	800001	1708OS5	Mon Aug	153	S	1	D	0.00001	M	Molar	1	21	Non-Sm	NCI-H46	101.504	
16	800001	1708OS5	Mon Aug	153	S	1	D	0.00001	M	Molar	1	3	Non-Sm	NCI-H52	69.5058	
17	800001	1708OS5	Mon Aug	153	S	1	D	0.00001	M	Molar	4	10	Colon C	COLO 20	113.037	
18	800001	1708OS5	Mon Aug	153	S	1	D	0.00001	M	Molar	4	2	Colon C	HCC-29	96.7456	
19	800001	1708OS5	Mon Aug	153	S	1	D	0.00001	M	Molar	4	3	Colon C	HCT-116	85.8348	
20	800001	1708OS5	Mon Aug	153	S	1	D	0.00001	M	Molar	4	15	Colon C	HCT-15	112.025	
21	800001	1708OS5	Mon Aug	153	S	1	D	0.00001	M	Molar	4	1	Colon C	HT29	87.8292	
22	800001	1708OS5	Mon Aug	153	S	1	D	0.00001	M	Molar	4	17	Colon C	KM12	97.48	
23	800001	1708OS5	Mon Aug	153	S	1	D	0.00001	M	Molar	4	9	Colon C	SW-620	94.1092	
24	800001	1708OS5	Mon Aug	153	S	1	D	0.00001	M	Molar	12	14	CNS Car	SF-268	100.243	
25	800001	1708OS5	Mon Aug	153	S	1	D	0.00001	M	Molar	12	15	CNS Car	SF-295	103.663	
26	800001	1708OS5	Mon Aug	153	S	1	D	0.00001	M	Molar	12	16	CNS Car	SF-539	105.041	
27	800001	1708OS5	Mon Aug	153	S	1	D	0.00001	M	Molar	12	2	CNS Car	SNB-19	95.3413	
28	800001	1708OS5	Mon Aug	153	S	1	D	0.00001	M	Molar	12	5	CNS Car	SNB-75	90.5763	
29	800001	1708OS5	Mon Aug	153	S	1	D	0.00001	M	Molar	12	9	CNS Car	U251	94.5448	
30	800001	1708OS5	Mon Aug	153	S	1	D	0.00001	M	Molar	10	1	Melanon	LOX IMV	99.0253	
31	800001	1708OS5	Mon Aug	153	S	1	D	0.00001	M	Molar	10	2	Melanon	MALME	106.868	
32	800001	1708OS5	Mon Aug	153	S	1	D	0.00001	M	Molar	10	14	Melanon	M14	104.469	
33	800001	1708OS5	Mon Aug	153	S	1	D	0.00001	M	Molar	5	11	Melanon	MDA-MI	102.568	
34	800001	1708OS5	Mon Aug	153	S	1	D	0.00001	M	Molar	10	5	Melanon	SK-MEL-	94.5115	
35	800001	1708OS5	Mon Aug	153	S	1	D	0.00001	M	Molar	10	8	Melanon	SK-MEL-	114.171	
36	800001	1708OS5	Mon Aug	153	S	1	D	0.00001	M	Molar	10	7	Melanon	SK-MEL-	93.2679	
37	800001	1708OS5	Mon Aug	153	S	1	D	0.00001	M	Molar	10	21	Melanon	UACC-2	85.2072	
38	800001	1708OS5	Mon Aug	153	S	1	D	0.00001	M	Molar	10	20	Melanon	UACC-6	88.4675	
39	800001	1708OS5	Mon Aug	153	S	1	D	0.00001	M	Molar	6	10	Ovarian (	IGROV1	93.2241	
40	800001	1708OS5	Mon Aug	153	S	1	D	0.00001	M	Molar	6	1	Ovarian (	OVCAR-	101.644	
41	800001	1708OS5	Mon Aug	153	S	1	D	0.00001	M	Molar	6	2	Ovarian (	OVCAR-	101.389	
42	800001	1708OS5	Mon Aug	153	S	1	D	0.00001	M	Molar	6	3	Ovarian (	OVCAR-	113.627	
43	800001	1708OS5	Mon Aug	153	S	1	D	0.00001	M	Molar	6	5	Ovarian (	OVCAR-	90.3568	
44	800001	1708OS5	Mon Aug	153	S	1	D	0.00001	M	Molar	5	2	Ovarian (	NCI/ADF	102.721	
45	800001	1708OS5	Mon Aug	153	S	1	D	0.00001	M	Molar	6	11	Ovarian (	SK-OV-3	96.4726	
46	800001	1708OS5	Mon Aug	153	S	1	D	0.00001	M	Molar	9	18	Renal Ca	786-0	106.733	
47	800001	1708OS5	Mon Aug	153	S	1	D	0.00001	M	Molar	9	13	Renal Ca	A498	93.0829	
48	800001	1708OS5	Mon Aug	153	S	1	D	0.00001	M	Molar	9	23	Renal Ca	ACHN	104.241	
49	800001	1708OS5	Mon Aug	153	S	1	D	0.00001	M	Molar	9	15	Renal Ca	CAKI-1	83.1227	
50	800001	1708OS5	Mon Aug	153	S	1	D	0.00001	M	Molar	9	16	Renal Ca	RXF 393	108.304	
51	800001	1708OS5	Mon Aug	153	S	1	D	0.00001	M	Molar	9	8	Renal Ca	SN12C	98.0871	
52	800001	1708OS5	Mon Aug	153	S	1	D	0.00001	M	Molar	9	24	Renal Ca	TK-10	130.172	
53	800001	1708OS5	Mon Aug	153	S	1	D	0.00001	M	Molar	9	4	Renal Ca	UO-31	86.2805	
54	800001	1708OS5	Mon Aug	153	S	1	D	0.00001	M	Molar	11	1	Prostate	PC-3	83.346	
55	800001	1708OS5	Mon Aug	153	S	1	D	0.00001	M	Molar	11	3	Prostate	DU-145	104	
56	800001	1708OS5	Mon Aug	153	S	1	D	0.00001	M	Molar	5	1	Breast C	MCF7	93.0491	
57	800001	1708OS5	Mon Aug	153	S	1	D	0.00001	M	Molar	5	5	Breast C	MDA-MI	95.9613	
58	800001	1708OS5	Mon Aug	153	S	1	D	0.00001	M	Molar	5	6	Breast C	HS 578T	91.6742	
59	800001	1708OS5	Mon Aug	153	S	1	D	0.00001	M	Molar	5	13	Breast C	BT-549	96.8658	
60	800001	1708OS5	Mon Aug	153	S	1	D	0.00001	M	Molar	5	14	Breast C	T-47D	89.9887	
61	800001	1708OS5	Mon Aug	153	S	1	D	0.00001	M	Molar	5	18	Breast C	MDA-MI	101.01	
62																

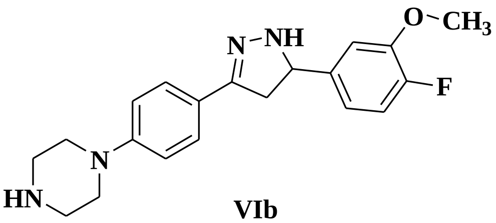

VIb

Developmental Therapeutics Program One Dose Mean Graph	NSC: D-799999 / 1	Conc: 1.00E-5 Molar	Test Date: Aug 14, 2017
	Experiment ID: 1708OS54		Report Date: Apr 18, 2020

Panel/Cell Line	Growth Percent
**Leukemia**	
CCRF-CEM	69.44
HL-60(TB)	76.95
K-562	58.92
MOLT-4	72.10
RPMI-8226	72.01
SR	67.42
**Non-Small Cell Lung Cancer**	
A549/ATCC	68.21
EKVX	95.41
HOP-62	86.01
HOP-92	57.13
NCI-H226	89.08
NCI-H23	75.49
NCI-H322M	89.15
NCI-H460	95.16
NCI-H522	66.27
**Colon Cancer**	
COLO 205	93.87
HCC-2998	104.57
HCT-116	61.10
HCT-15	85.57
HT29	20.27
KM12	93.81
SW-620	91.67
**CNS Cancer**	
SF-268	93.72
SF-295	97.70
SF-539	84.21
SNB-19	95.65
SNB-75	88.85
U251	78.59
**Melanoma**	
LOX IMVI	79.56
MALME-3M	102.13
M14	84.68
MDA-MB-435	101.87
SK-MEL-2	87.70
SK-MEL-28	109.51
SK-MEL-5	80.94
UACC-257	80.93
UACC-62	86.00
**Ovarian Cancer**	
IGROV1	82.04
OVCAR-3	91.15
OVCAR-4	91.49
OVCAR-5	101.51
OVCAR-8	84.09
NCI/ADR-RES	104.69
SK-OV-3	83.58
**Renal Cancer**	
786-0	94.50
A498	90.59
ACHN	101.47
CAKI-1	86.62
RXF 393	95.32
SN12C	83.08
TK-10	113.60
UO-31	76.56
**Prostate Cancer**	
PC-3	64.23
DU-145	103.62
**Breast Cancer**	
MCF7	68.65
MDA-MB-231/ATCC	81.98
HS 578T	84.17
BT-549	70.80
T-47D	73.62
MDA-MB-468	96.57
Mean	84.43
Delta	64.16
Range	93.33

Mean Growth Percent - Growth Percent

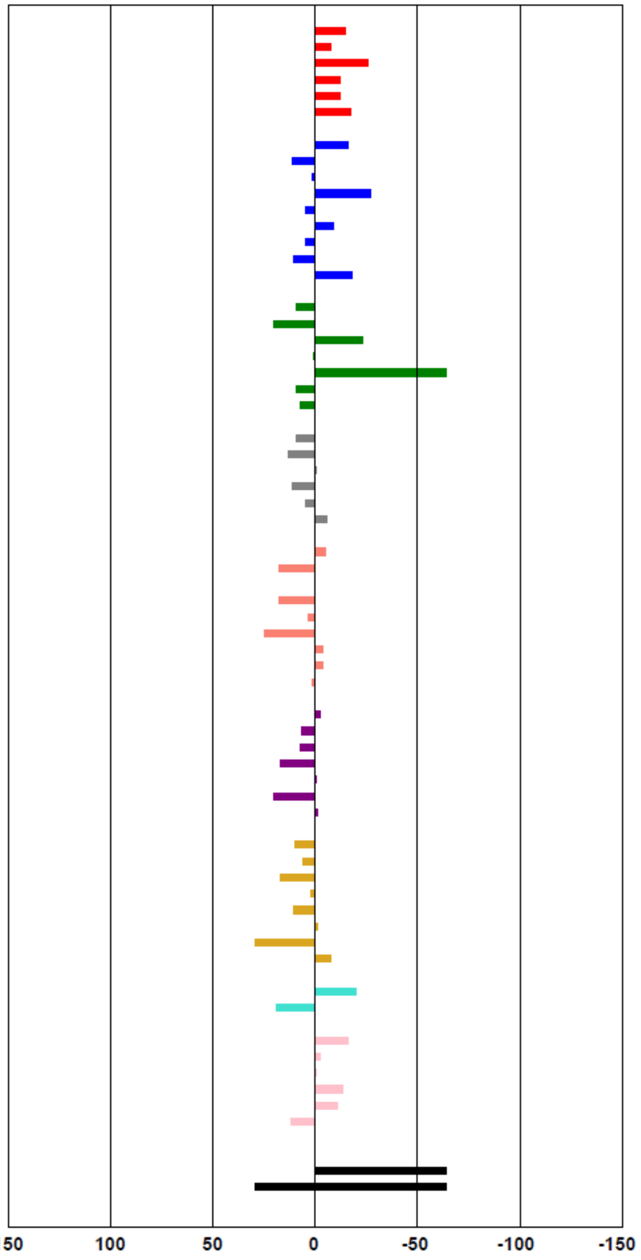

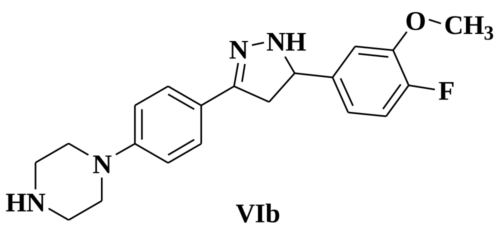

VIb

A1 | NSC

	A	B	C	D	E	F	G	H	I	J	K	L	M	N	O	P
1	NSC	EXPID	PLANDA	TESTSE	PREFIX	SAMPLE	DISCREI	CONC	CONCUI	CONCUI	PANELN	CELLNB	PANELN	CELLNA	GIPRCNT	
2	799999	1708OS5	Mon Aug	133	S	1	D	0.00001	M	Molar	7	3	Leukemi	CCRF-C	69.4377	
3	799999	1708OS5	Mon Aug	133	S	1	D	0.00001	M	Molar	7	8	Leukemi	HL-60(TE	76.9489	
4	799999	1708OS5	Mon Aug	133	S	1	D	0.00001	M	Molar	7	5	Leukemi	K-562	58.915	
5	799999	1708OS5	Mon Aug	133	S	1	D	0.00001	M	Molar	7	6	Leukemi	MOLT-4	72.1014	
6	799999	1708OS5	Mon Aug	133	S	1	D	0.00001	M	Molar	7	10	Leukemi	RPMI-82	72.0067	
7	799999	1708OS5	Mon Aug	133	S	1	D	0.00001	M	Molar	7	19	Leukemi	SR	67.4222	
8	799999	1708OS5	Mon Aug	133	S	1	D	0.00001	M	Molar	1	4	Non-Sm	A549/AT	68.2079	
9	799999	1708OS5	Mon Aug	133	S	1	D	0.00001	M	Molar	1	8	Non-Sm	EKVX	95.4054	
10	799999	1708OS5	Mon Aug	133	S	1	D	0.00001	M	Molar	1	26	Non-Sm	HOP-62	86.0135	
11	799999	1708OS5	Mon Aug	133	S	1	D	0.00001	M	Molar	1	29	Non-Sm	HOP-92	57.1292	
12	799999	1708OS5	Mon Aug	133	S	1	D	0.00001	M	Molar	1	13	Non-Sm	NCI-H22	89.0776	
13	799999	1708OS5	Mon Aug	133	S	1	D	0.00001	M	Molar	1	1	Non-Sm	NCI-H23	75.4886	
14	799999	1708OS5	Mon Aug	133	S	1	D	0.00001	M	Molar	1	17	Non-Sm	NCI-H32	89.1519	
15	799999	1708OS5	Mon Aug	133	S	1	D	0.00001	M	Molar	1	21	Non-Sm	NCI-H46	95.1636	
16	799999	1708OS5	Mon Aug	133	S	1	D	0.00001	M	Molar	1	3	Non-Sm	NCI-H52	66.2655	
17	799999	1708OS5	Mon Aug	133	S	1	D	0.00001	M	Molar	4	10	Colon C	COLO 20	93.8722	
18	799999	1708OS5	Mon Aug	133	S	1	D	0.00001	M	Molar	4	2	Colon C	HCC-29	104.565	
19	799999	1708OS5	Mon Aug	133	S	1	D	0.00001	M	Molar	4	3	Colon C	HCT-116	61.1043	
20	799999	1708OS5	Mon Aug	133	S	1	D	0.00001	M	Molar	4	15	Colon C	HCT-15	85.5705	
21	799999	1708OS5	Mon Aug	133	S	1	D	0.00001	M	Molar	4	1	Colon C	HT29	20.2691	
22	799999	1708OS5	Mon Aug	133	S	1	D	0.00001	M	Molar	4	17	Colon C	KM12	93.8055	
23	799999	1708OS5	Mon Aug	133	S	1	D	0.00001	M	Molar	4	9	Colon C	SW-620	91.6712	
24	799999	1708OS5	Mon Aug	133	S	1	D	0.00001	M	Molar	12	14	CNS Car	SF-268	93.7175	
25	799999	1708OS5	Mon Aug	133	S	1	D	0.00001	M	Molar	12	15	CNS Car	SF-295	97.695	
26	799999	1708OS5	Mon Aug	133	S	1	D	0.00001	M	Molar	12	16	CNS Car	SF-539	84.2062	
27	799999	1708OS5	Mon Aug	133	S	1	D	0.00001	M	Molar	12	2	CNS Car	SNB-19	95.6468	
28	799999	1708OS5	Mon Aug	133	S	1	D	0.00001	M	Molar	12	5	CNS Car	SNB-75	88.8473	
29	799999	1708OS5	Mon Aug	133	S	1	D	0.00001	M	Molar	12	9	CNS Car	U251	78.5884	
30	799999	1708OS5	Mon Aug	133	S	1	D	0.00001	M	Molar	10	1	Melanon	LOX IMV	79.5577	
31	799999	1708OS5	Mon Aug	133	S	1	D	0.00001	M	Molar	10	2	Melanon	MALME	102.129	
32	799999	1708OS5	Mon Aug	133	S	1	D	0.00001	M	Molar	10	14	Melanon	M14	84.6833	
33	799999	1708OS5	Mon Aug	133	S	1	D	0.00001	M	Molar	5	11	Melanon	MDA-MI	101.869	
34	799999	1708OS5	Mon Aug	133	S	1	D	0.00001	M	Molar	10	5	Melanon	SK-MEL-	87.6977	
35	799999	1708OS5	Mon Aug	133	S	1	D	0.00001	M	Molar	10	8	Melanon	SK-MEL-	109.512	
36	799999	1708OS5	Mon Aug	133	S	1	D	0.00001	M	Molar	10	7	Melanon	SK-MEL-	80.9433	
37	799999	1708OS5	Mon Aug	133	S	1	D	0.00001	M	Molar	10	21	Melanon	UACC-2	80.9349	
38	799999	1708OS5	Mon Aug	133	S	1	D	0.00001	M	Molar	10	20	Melanon	UACC-6	86.0022	
39	799999	1708OS5	Mon Aug	133	S	1	D	0.00001	M	Molar	6	10	Ovarian (	IGROV1	82.0413	
40	799999	1708OS5	Mon Aug	133	S	1	D	0.00001	M	Molar	6	1	Ovarian (	OVCAR-	91.1451	
41	799999	1708OS5	Mon Aug	133	S	1	D	0.00001	M	Molar	6	2	Ovarian (	OVCAR-	91.4863	
42	799999	1708OS5	Mon Aug	133	S	1	D	0.00001	M	Molar	6	3	Ovarian (	OVCAR-	101.512	
43	799999	1708OS5	Mon Aug	133	S	1	D	0.00001	M	Molar	6	5	Ovarian (	OVCAR-	84.0872	
44	799999	1708OS5	Mon Aug	133	S	1	D	0.00001	M	Molar	5	2	Ovarian (	NCI/ADF	104.69	
45	799999	1708OS5	Mon Aug	133	S	1	D	0.00001	M	Molar	6	11	Ovarian (	SK-OV-3	83.5786	
46	799999	1708OS5	Mon Aug	133	S	1	D	0.00001	M	Molar	9	18	Renal Ca	786-0	94.5011	
47	799999	1708OS5	Mon Aug	133	S	1	D	0.00001	M	Molar	9	13	Renal Ca	A498	90.5925	
48	799999	1708OS5	Mon Aug	133	S	1	D	0.00001	M	Molar	9	23	Renal Ca	ACHN	101.474	
49	799999	1708OS5	Mon Aug	133	S	1	D	0.00001	M	Molar	9	15	Renal Ca	CAKI-1	86.6237	
50	799999	1708OS5	Mon Aug	133	S	1	D	0.00001	M	Molar	9	16	Renal Ca	RXF 393	95.3233	
51	799999	1708OS5	Mon Aug	133	S	1	D	0.00001	M	Molar	9	8	Renal Ca	SN12C	83.0777	
52	799999	1708OS5	Mon Aug	133	S	1	D	0.00001	M	Molar	9	24	Renal Ca	TK-10	113.604	
53	799999	1708OS5	Mon Aug	133	S	1	D	0.00001	M	Molar	9	4	Renal Ca	UO-31	76.5557	
54	799999	1708OS5	Mon Aug	133	S	1	D	0.00001	M	Molar	11	1	Prostate	PC-3	64.2337	
55	799999	1708OS5	Mon Aug	133	S	1	D	0.00001	M	Molar	11	3	Prostate	DU-145	103.625	
56	799999	1708OS5	Mon Aug	133	S	1	D	0.00001	M	Molar	5	1	Breast C	MCF7	68.6514	
57	799999	1708OS5	Mon Aug	133	S	1	D	0.00001	M	Molar	5	5	Breast C	MDA-MI	81.9785	
58	799999	1708OS5	Mon Aug	133	S	1	D	0.00001	M	Molar	5	6	Breast C	HS 578T	84.1707	
59	799999	1708OS5	Mon Aug	133	S	1	D	0.00001	M	Molar	5	13	Breast C	BT-549	70.804	
60	799999	1708OS5	Mon Aug	133	S	1	D	0.00001	M	Molar	5	14	Breast C	T-47D	73.6154	
61	799999	1708OS5	Mon Aug	133	S	1	D	0.00001	M	Molar	5	18	Breast C	MDA-MI	96.5683	
62																
63																
64																

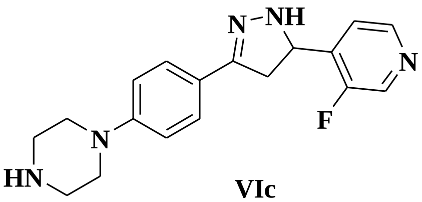

VIc

Developmental Therapeutics Program One Dose Mean Graph	NSC: D-800000 / 1	Conc: 1.00E-5 Molar	Test Date: Aug 14, 2017
	Experiment ID: 1708OS54		Report Date: Apr 18, 2020

Panel/Cell Line	Growth Percent
**Leukemia**	
CCRF-CEM	80.81
HL-60(TB)	77.32
K-562	77.92
MOLT-4	74.15
RPMI-8226	92.51
SR	90.38
**Non-Small Cell Lung Cancer**	
A549/ATCC	76.20
EKVX	91.04
HOP-62	83.16
HOP-92	72.42
NCI-H226	82.88
NCI-H23	83.85
NCI-H322M	89.29
NCI-H460	94.92
NCI-H522	81.93
**Colon Cancer**	
COLO 205	95.69
HCC-2998	101.72
HCT-116	73.82
HCT-15	97.59
HT29	60.16
KM12	96.23
SW-620	93.46
**CNS Cancer**	
SF-268	107.32
SF-295	98.00
SF-539	104.39
SNB-19	97.45
SNB-75	101.50
U251	95.06
**Melanoma**	
LOX IMVI	94.31
MALME-3M	93.43
M14	95.86
MDA-MB-435	105.18
SK-MEL-2	92.72
SK-MEL-28	115.80
SK-MEL-5	85.78
UACC-257	83.07
UACC-62	81.93
**Ovarian Cancer**	
IGROV1	67.96
OVCAR-3	98.79
OVCAR-4	108.25
OVCAR-5	111.45
OVCAR-8	95.06
NCI/ADR-RES	106.03
SK-OV-3	85.00
**Renal Cancer**	
786-0	111.09
A498	97.17
ACHN	99.87
CAKI-1	80.62
RXF 393	104.89
SN12C	89.56
TK-10	122.80
UO-31	77.18
**Prostate Cancer**	
PC-3	70.34
DU-145	107.16
**Breast Cancer**	
MCF7	73.03
MDA-MB-231/ATCC	84.58
HS 578T	89.15
BT-549	85.33
T-47D	82.20
MDA-MB-468	114.26
Mean	91.35
Delta	31.19
Range	62.64

Mean Growth Percent - Growth Percent

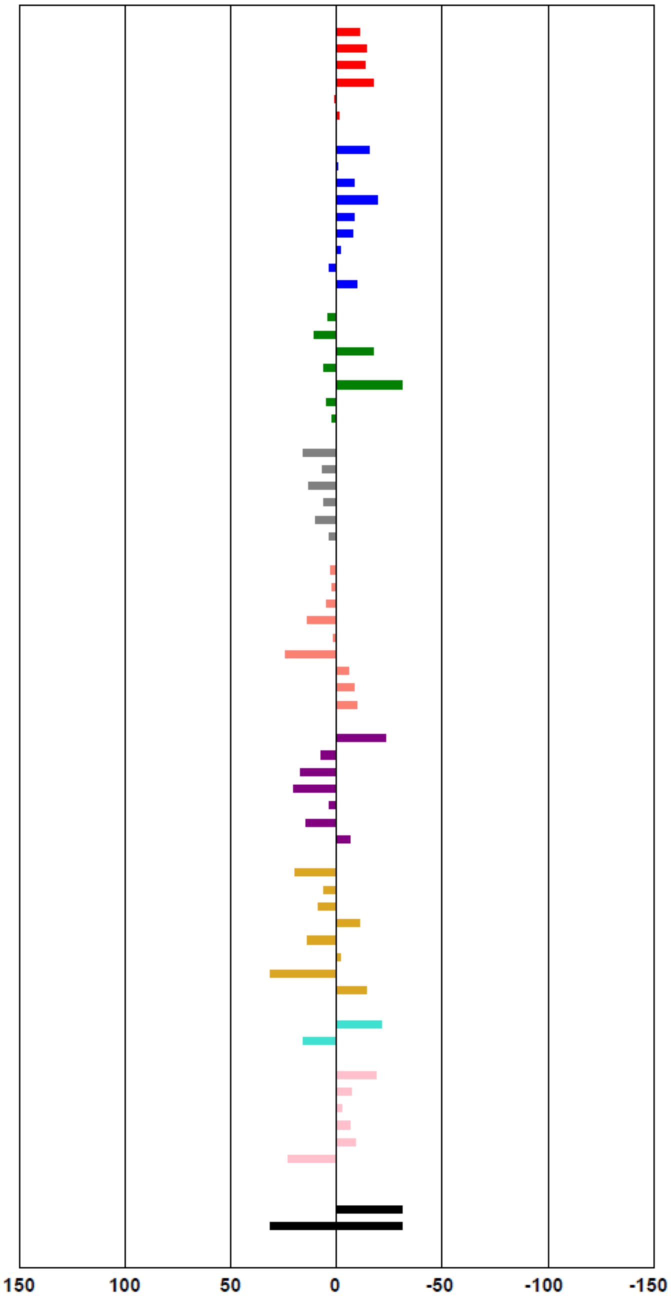

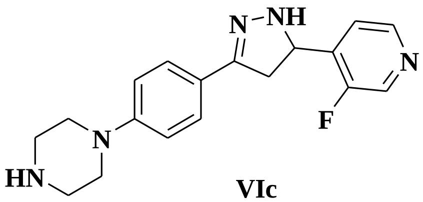

VIc

A1 | NSC

	A	B	C	D	E	F	G	H	I	J	K	L	M	N	O	P
1	NSC	EXPID	PLANDA	TESTSE	PREFIX	SAMPLE	DISCREI	CONC	CONCUI	CONCUI	PANELN	CELLNB	PANELN	CELLNA	GIPRCNT	
2	800000	1708OS5	Mon Aug	143	S	1	D	0.00001	M	Molar	7	3	Leukemi	CCRF-C	80.8113	
3	800000	1708OS5	Mon Aug	143	S	1	D	0.00001	M	Molar	7	8	Leukemi	HL-60(TE	77.3207	
4	800000	1708OS5	Mon Aug	143	S	1	D	0.00001	M	Molar	7	5	Leukemi	K-562	77.9245	
5	800000	1708OS5	Mon Aug	143	S	1	D	0.00001	M	Molar	7	6	Leukemi	MOLT-4	74.1549	
6	800000	1708OS5	Mon Aug	143	S	1	D	0.00001	M	Molar	7	10	Leukemi	RPMI-82	92.5113	
7	800000	1708OS5	Mon Aug	143	S	1	D	0.00001	M	Molar	7	19	Leukemi	SR	90.3809	
8	800000	1708OS5	Mon Aug	143	S	1	D	0.00001	M	Molar	1	4	Non-Sm	A549/AT	76.1998	
9	800000	1708OS5	Mon Aug	143	S	1	D	0.00001	M	Molar	1	8	Non-Sm	EKVX	91.0423	
10	800000	1708OS5	Mon Aug	143	S	1	D	0.00001	M	Molar	1	26	Non-Sm	HOP-62	83.1608	
11	800000	1708OS5	Mon Aug	143	S	1	D	0.00001	M	Molar	1	29	Non-Sm	HOP-92	72.4175	
12	800000	1708OS5	Mon Aug	143	S	1	D	0.00001	M	Molar	1	13	Non-Sm	NCI-H22	82.8754	
13	800000	1708OS5	Mon Aug	143	S	1	D	0.00001	M	Molar	1	1	Non-Sm	NCI-H23	83.846	
14	800000	1708OS5	Mon Aug	143	S	1	D	0.00001	M	Molar	1	17	Non-Sm	NCI-H32	89.2938	
15	800000	1708OS5	Mon Aug	143	S	1	D	0.00001	M	Molar	1	21	Non-Sm	NCI-H46	94.9227	
16	800000	1708OS5	Mon Aug	143	S	1	D	0.00001	M	Molar	1	3	Non-Sm	NCI-H52	81.9278	
17	800000	1708OS5	Mon Aug	143	S	1	D	0.00001	M	Molar	4	10	Colon C	COLO 20	95.6917	
18	800000	1708OS5	Mon Aug	143	S	1	D	0.00001	M	Molar	4	2	Colon C	HCC-299	101.719	
19	800000	1708OS5	Mon Aug	143	S	1	D	0.00001	M	Molar	4	3	Colon C	HCT-116	73.8224	
20	800000	1708OS5	Mon Aug	143	S	1	D	0.00001	M	Molar	4	15	Colon C	HCT-15	97.5886	
21	800000	1708OS5	Mon Aug	143	S	1	D	0.00001	M	Molar	4	1	Colon C	HT29	60.158	
22	800000	1708OS5	Mon Aug	143	S	1	D	0.00001	M	Molar	4	17	Colon C	KM12	96.232	
23	800000	1708OS5	Mon Aug	143	S	1	D	0.00001	M	Molar	4	9	Colon C	SW-620	93.4636	
24	800000	1708OS5	Mon Aug	143	S	1	D	0.00001	M	Molar	12	14	CNS Car	SF-268	107.316	
25	800000	1708OS5	Mon Aug	143	S	1	D	0.00001	M	Molar	12	15	CNS Car	SF-295	98.0044	
26	800000	1708OS5	Mon Aug	143	S	1	D	0.00001	M	Molar	12	16	CNS Car	SF-539	104.387	
27	800000	1708OS5	Mon Aug	143	S	1	D	0.00001	M	Molar	12	2	CNS Car	SNB-19	97.4539	
28	800000	1708OS5	Mon Aug	143	S	1	D	0.00001	M	Molar	12	5	CNS Car	SNB-75	101.499	
29	800000	1708OS5	Mon Aug	143	S	1	D	0.00001	M	Molar	12	9	CNS Car	U251	95.0588	
30	800000	1708OS5	Mon Aug	143	S	1	D	0.00001	M	Molar	10	1	Melanon	LOX IMV	94.3125	
31	800000	1708OS5	Mon Aug	143	S	1	D	0.00001	M	Molar	10	2	Melanon	MALME	93.4318	
32	800000	1708OS5	Mon Aug	143	S	1	D	0.00001	M	Molar	10	14	Melanon	M14	95.8636	
33	800000	1708OS5	Mon Aug	143	S	1	D	0.00001	M	Molar	5	11	Melanon	MDA-MI	105.182	
34	800000	1708OS5	Mon Aug	143	S	1	D	0.00001	M	Molar	10	5	Melanon	SK-MEL-	92.7206	
35	800000	1708OS5	Mon Aug	143	S	1	D	0.00001	M	Molar	10	8	Melanon	SK-MEL-	115.799	
36	800000	1708OS5	Mon Aug	143	S	1	D	0.00001	M	Molar	10	7	Melanon	SK-MEL-	85.7776	
37	800000	1708OS5	Mon Aug	143	S	1	D	0.00001	M	Molar	10	21	Melanon	UACC-2	83.0735	
38	800000	1708OS5	Mon Aug	143	S	1	D	0.00001	M	Molar	10	20	Melanon	UACC-6	81.9328	
39	800000	1708OS5	Mon Aug	143	S	1	D	0.00001	M	Molar	6	10	Ovarian (	IGROV1	67.9582	
40	800000	1708OS5	Mon Aug	143	S	1	D	0.00001	M	Molar	6	1	Ovarian (	OVCAR-	98.7926	
41	800000	1708OS5	Mon Aug	143	S	1	D	0.00001	M	Molar	6	2	Ovarian (	OVCAR-	108.252	
42	800000	1708OS5	Mon Aug	143	S	1	D	0.00001	M	Molar	6	3	Ovarian (	OVCAR-	111.455	
43	800000	1708OS5	Mon Aug	143	S	1	D	0.00001	M	Molar	6	5	Ovarian (	OVCAR-	95.0576	
44	800000	1708OS5	Mon Aug	143	S	1	D	0.00001	M	Molar	5	2	Ovarian (	NCI/ADF	106.035	
45	800000	1708OS5	Mon Aug	143	S	1	D	0.00001	M	Molar	6	11	Ovarian (	SK-OV-3	85	
46	800000	1708OS5	Mon Aug	143	S	1	D	0.00001	M	Molar	9	18	Renal Ca	786-0	111.087	
47	800000	1708OS5	Mon Aug	143	S	1	D	0.00001	M	Molar	9	13	Renal Ca	A498	97.1731	
48	800000	1708OS5	Mon Aug	143	S	1	D	0.00001	M	Molar	9	23	Renal Ca	ACHN	99.8655	
49	800000	1708OS5	Mon Aug	143	S	1	D	0.00001	M	Molar	9	15	Renal Ca	CAKI-1	80.6225	
50	800000	1708OS5	Mon Aug	143	S	1	D	0.00001	M	Molar	9	16	Renal Ca	RXF 393	104.893	
51	800000	1708OS5	Mon Aug	143	S	1	D	0.00001	M	Molar	9	8	Renal Ca	SN12C	89.5574	
52	800000	1708OS5	Mon Aug	143	S	1	D	0.00001	M	Molar	9	24	Renal Ca	TK-10	122.802	
53	800000	1708OS5	Mon Aug	143	S	1	D	0.00001	M	Molar	9	4	Renal Ca	UO-31	77.183	
54	800000	1708OS5	Mon Aug	143	S	1	D	0.00001	M	Molar	11	1	Prostate	PC-3	70.3411	
55	800000	1708OS5	Mon Aug	143	S	1	D	0.00001	M	Molar	11	3	Prostate	DU-145	107.156	
56	800000	1708OS5	Mon Aug	143	S	1	D	0.00001	M	Molar	5	1	Breast C	MCF7	73.0257	
57	800000	1708OS5	Mon Aug	143	S	1	D	0.00001	M	Molar	5	5	Breast C	MDA-MI	84.5839	
58	800000	1708OS5	Mon Aug	143	S	1	D	0.00001	M	Molar	5	6	Breast C	HS 578T	89.1513	
59	800000	1708OS5	Mon Aug	143	S	1	D	0.00001	M	Molar	5	13	Breast C	BT-549	85.3253	
60	800000	1708OS5	Mon Aug	143	S	1	D	0.00001	M	Molar	5	14	Breast C	T-47D	82.202	
61	800000	1708OS5	Mon Aug	143	S	1	D	0.00001	M	Molar	5	18	Breast C	MDA-MI	114.258	
62																
63																
64																

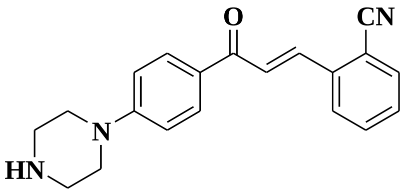
O
CN
N
HN

Va

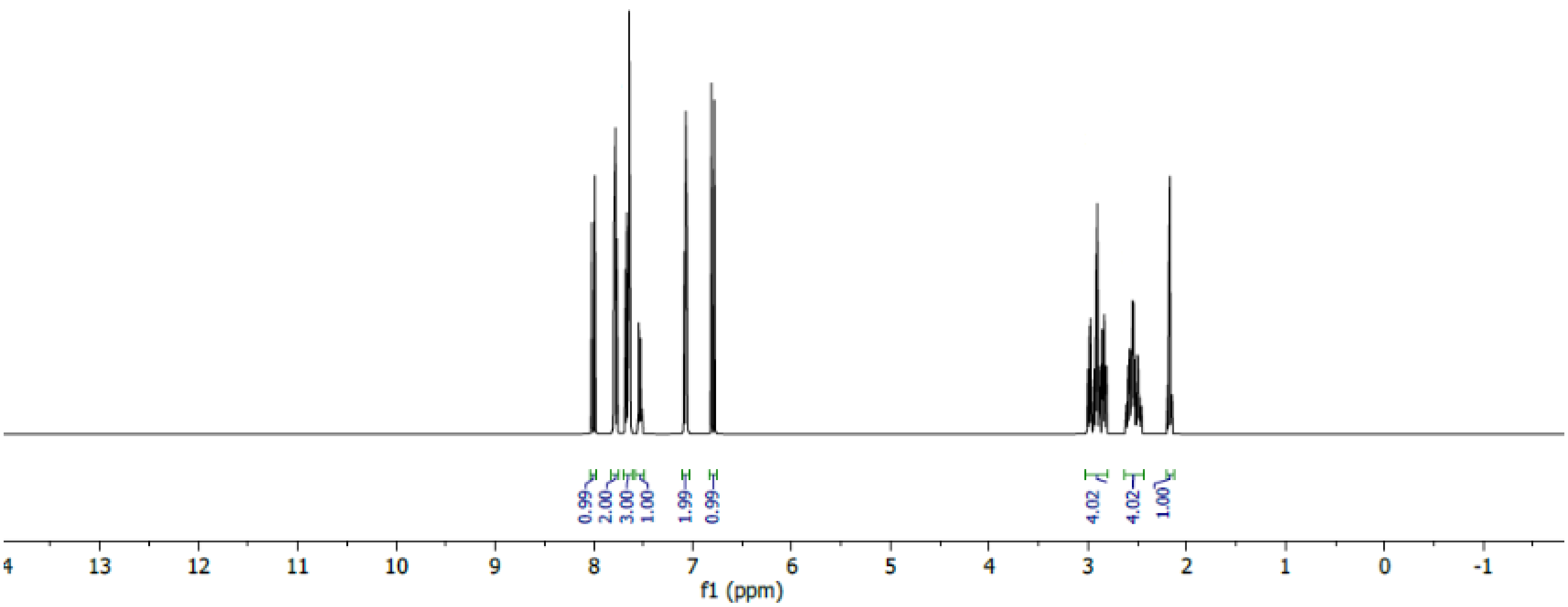
0.99
2.00
3.00
1.00
1.99
0.99
4.02
4.02
1.00
13
12
11
10
9
8
7
6
5
4
3
2
1
0
-1
f1 (ppm)

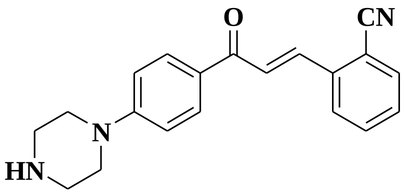
O
CN
N
HN

Va

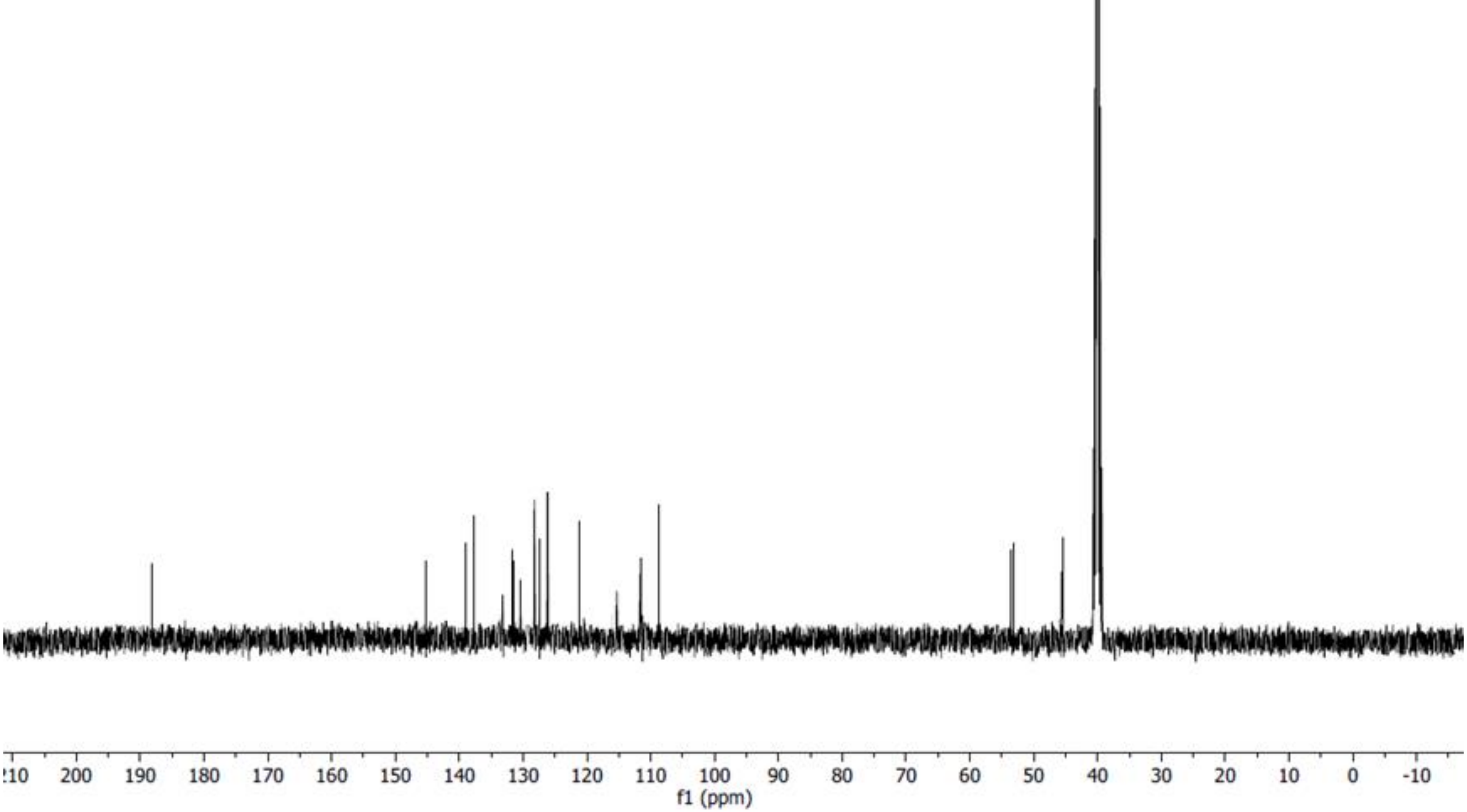
210 200 190 180 170 160 150 140 130 120 110 100 90 80 70 60 50 40 30 20 10 0 -10
f1 (ppm)

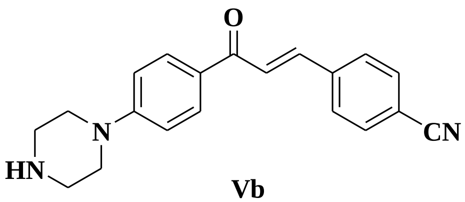
O
N
HN
CN

Vb

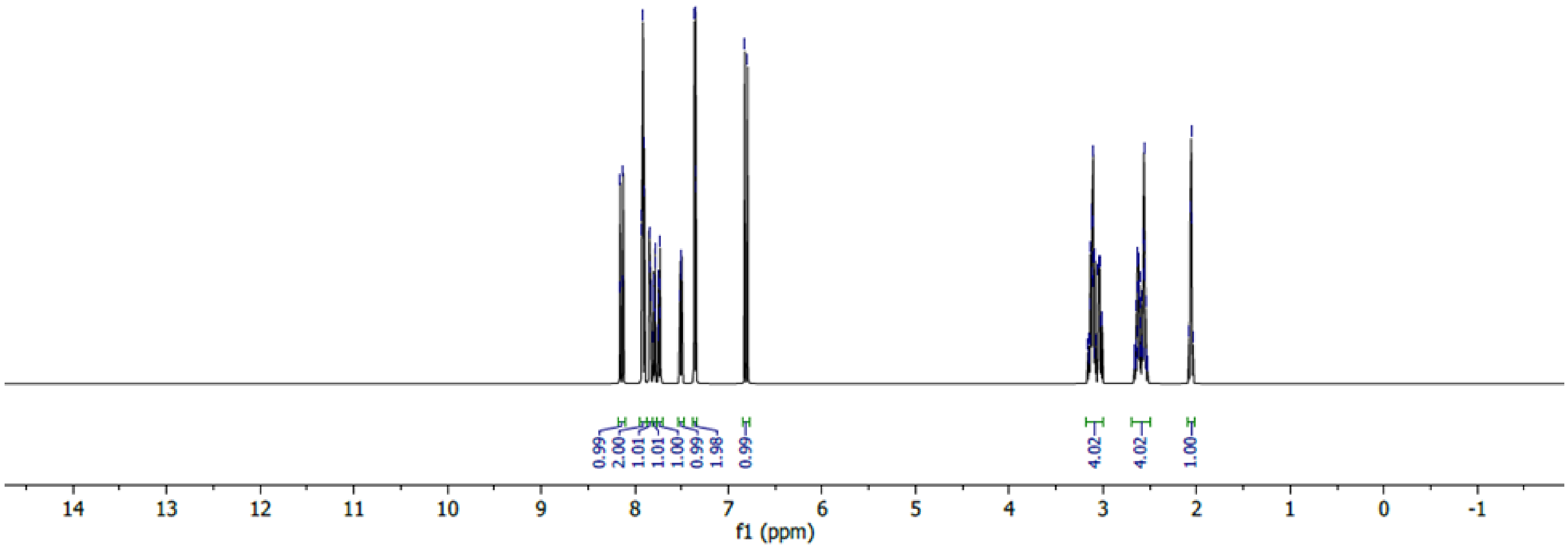
0.99
2.00
1.01
1.01
1.00
0.99
1.98
0.99
4.02
4.02
1.00
14
13
12
11
10
9
8
7
6
5
4
3
2
1
0
-1
f1 (ppm)

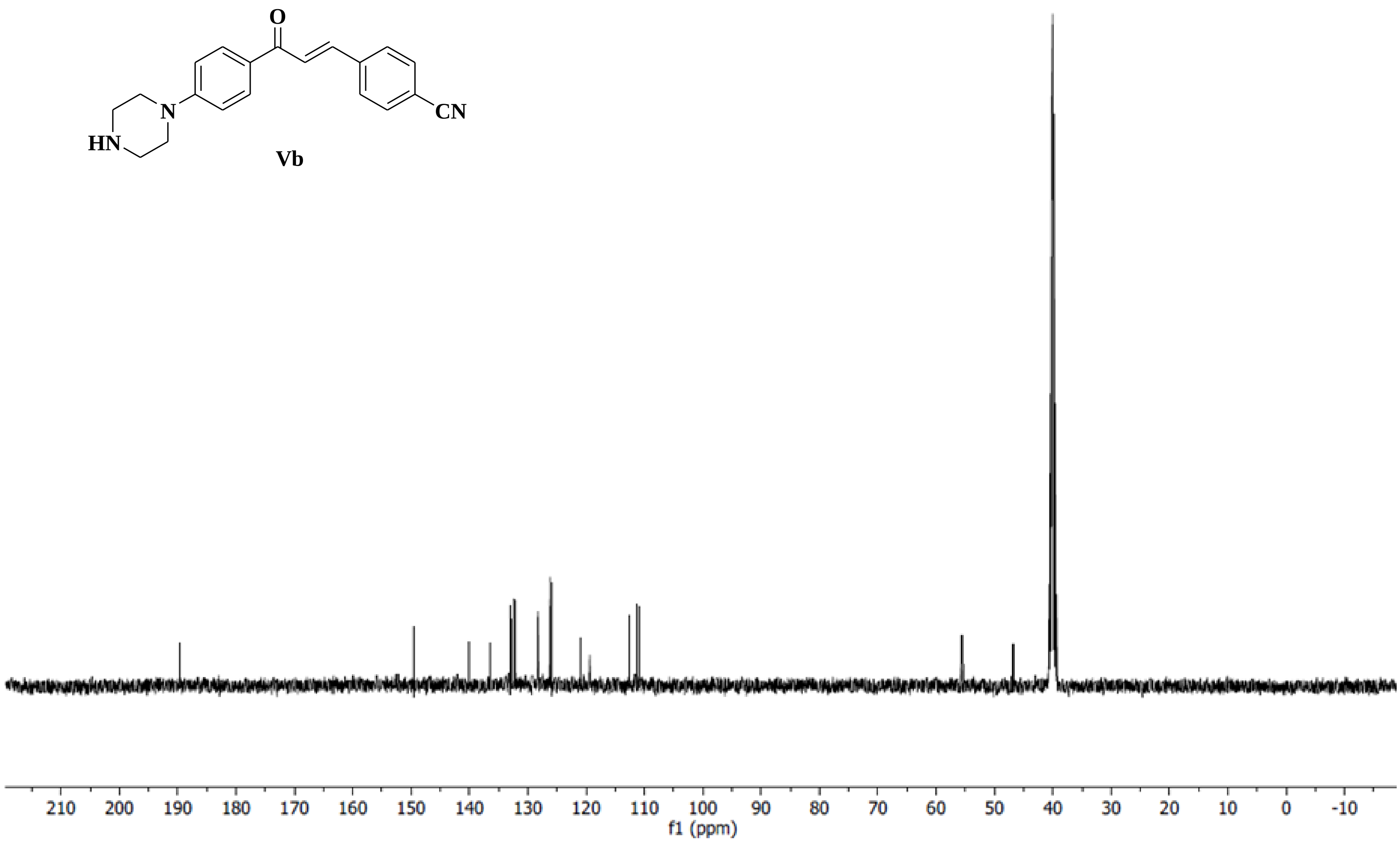

O
N
HN
CN
210 200 190 180 170 160 150 140 130 120 110 100 90 80 70 60 50 40 30 20 10 0 -10
f1 (ppm)

Vb

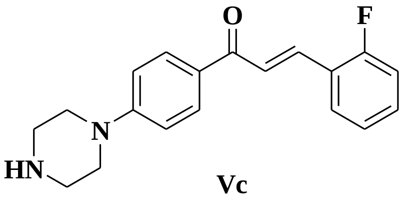

Vc

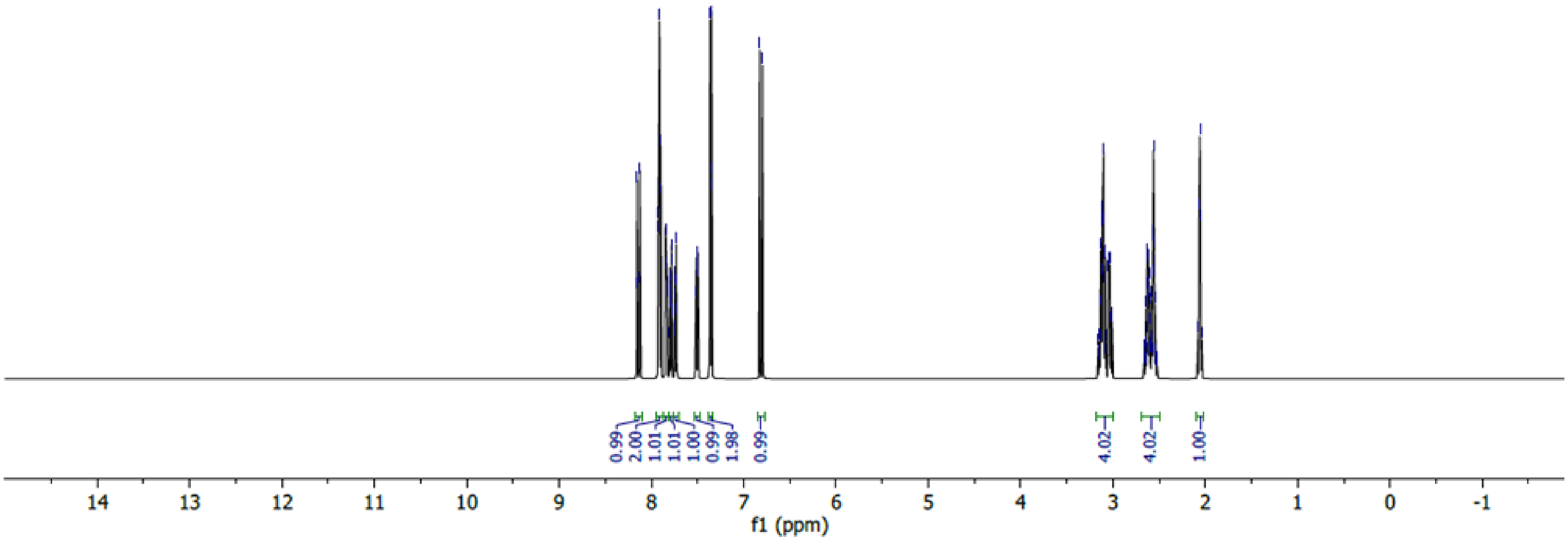
0.99
2.00
1.01
1.01
1.00
0.99
1.98
0.99
4.02
4.02
1.00
14
13
12
11
10
9
8
7
6
5
4
3
2
1
0
-1
f1 (ppm)

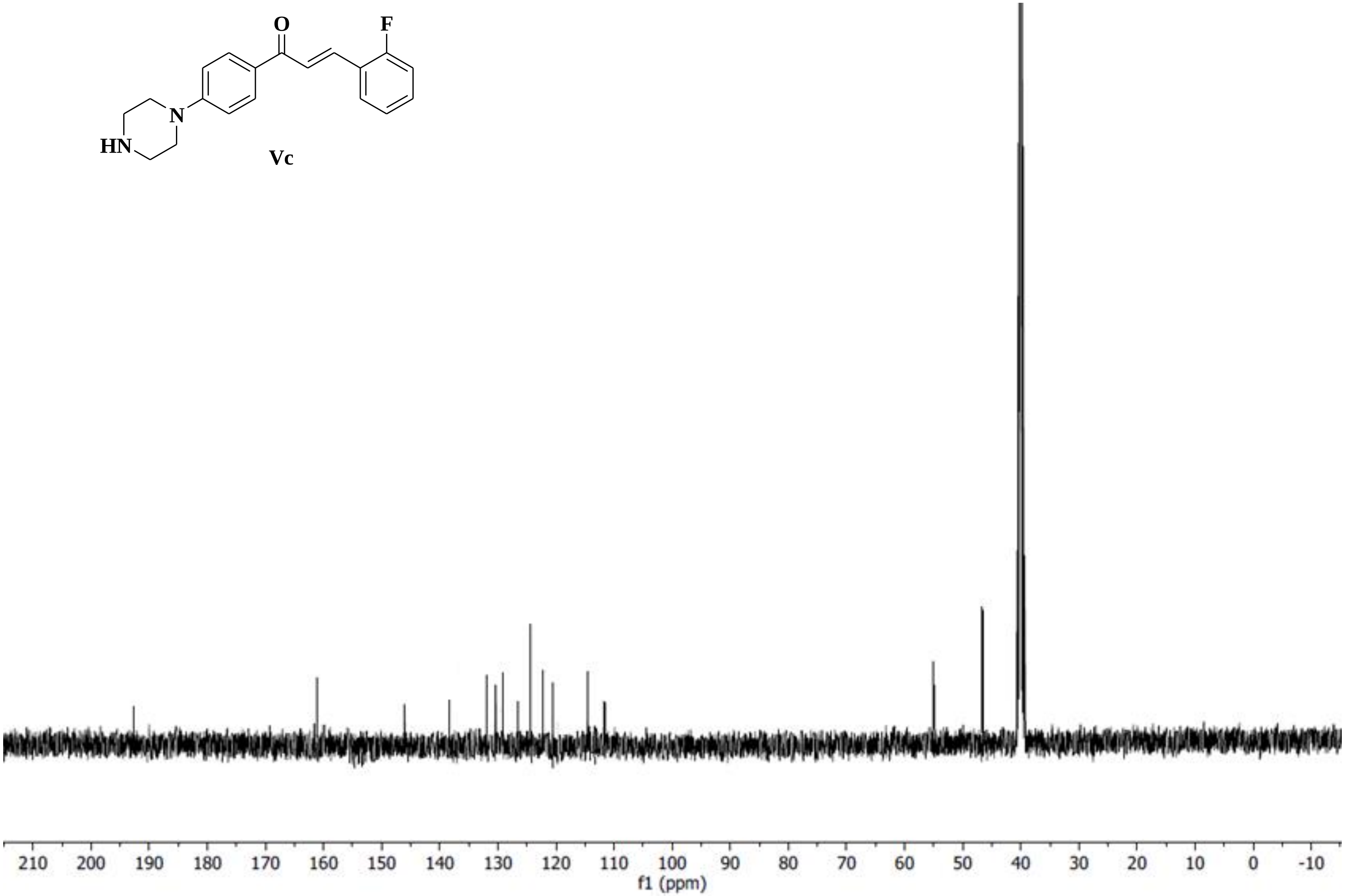
O
F
N
HN

Vc

210 200 190 180 170 160 150 140 130 120 110 100 90 80 70 60 50 40 30 20 10 0 -10
f1 (ppm)

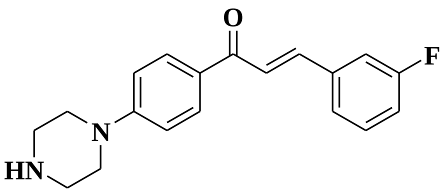
O
F
N
HN

Vd

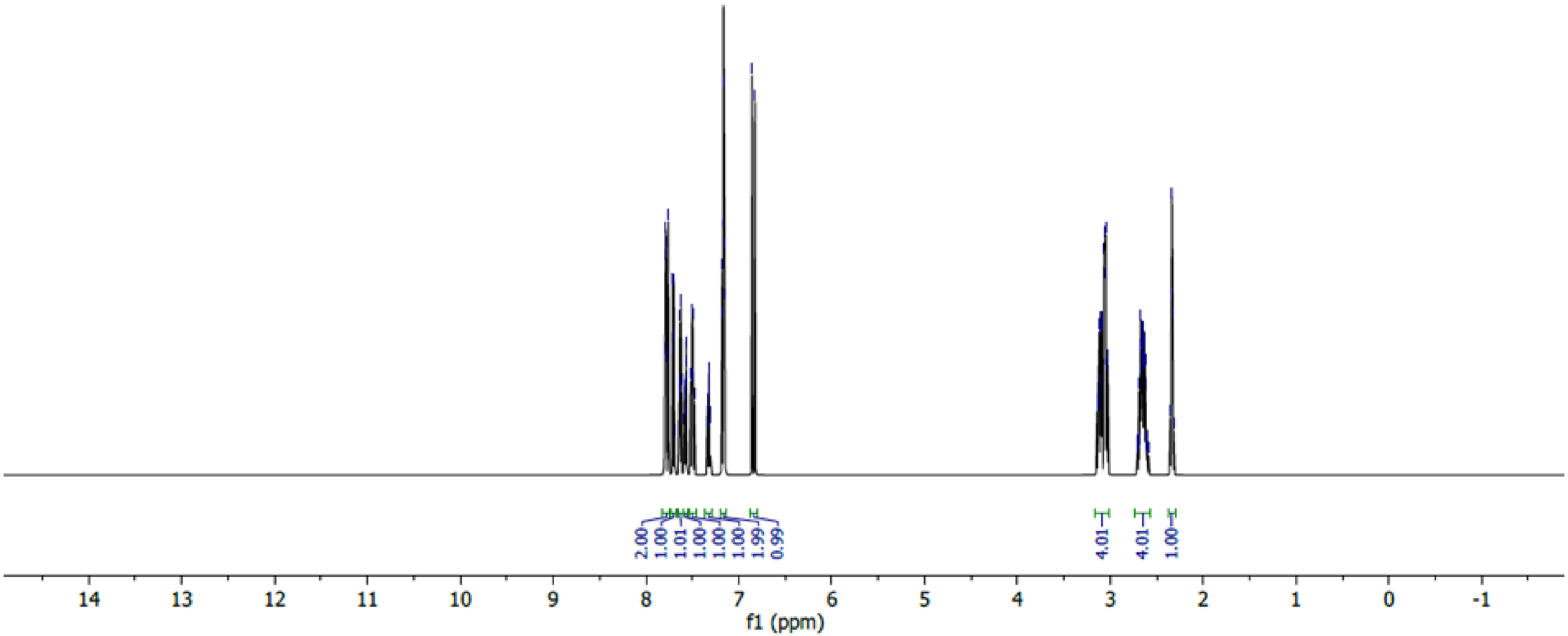
2.00
1.00
1.01
1.00
1.00
1.00
1.99
0.99
4.01
4.01
1.00
14
13
12
11
10
9
8
7
6
5
4
3
2
1
0
-1
f1 (ppm)

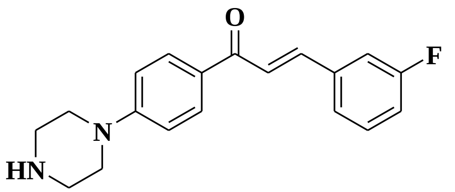
O
F
N
HN

Vd

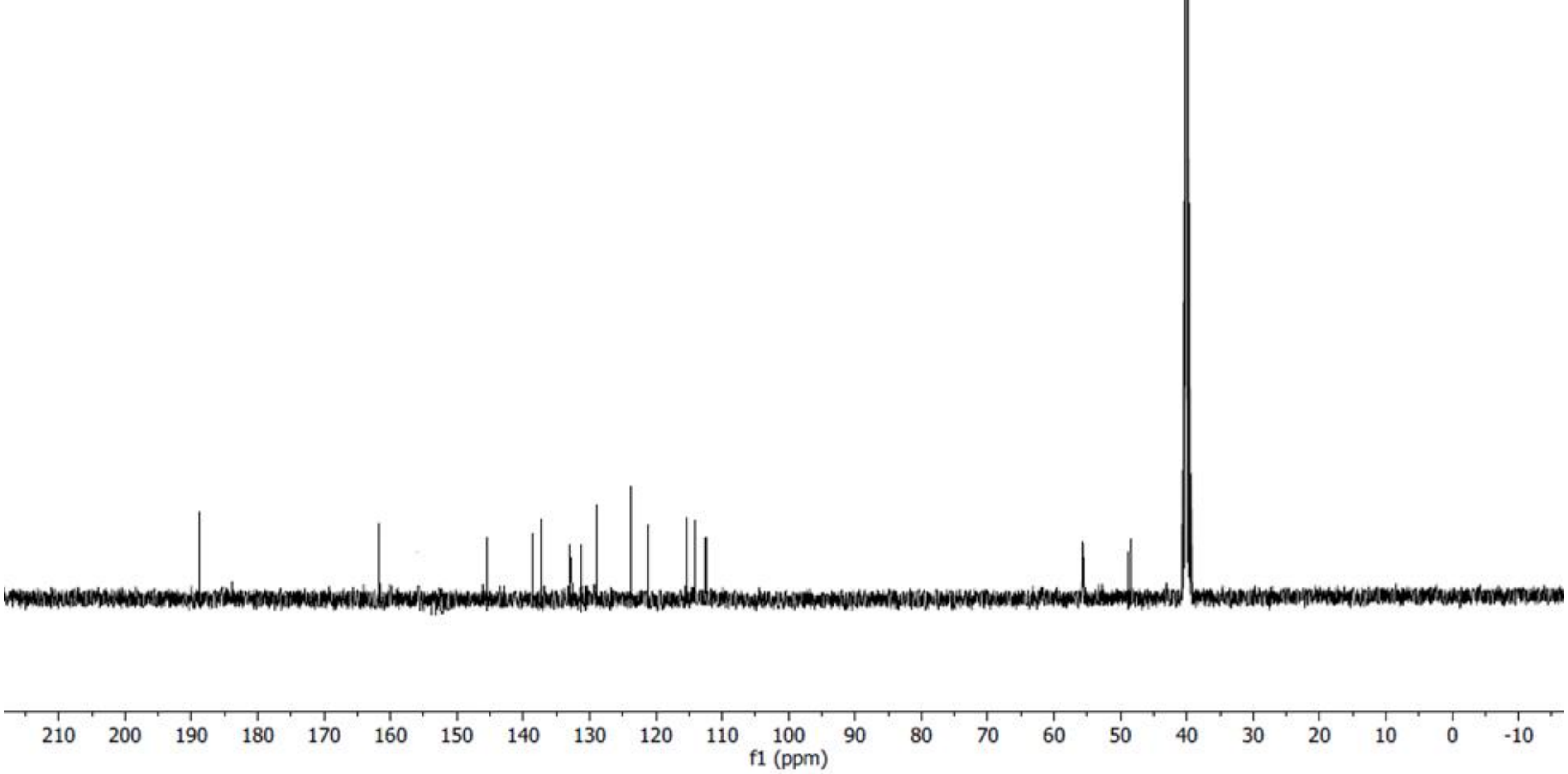
210 200 190 180 170 160 150 140 130 120 110 100 90 80 70 60 50 40 30 20 10 0 -10
f1 (ppm)

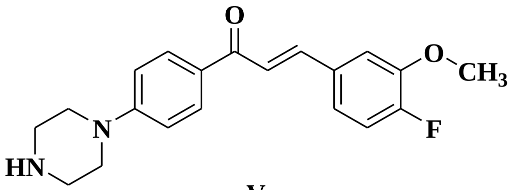
O
O
CH3
N
HN
F

Ve

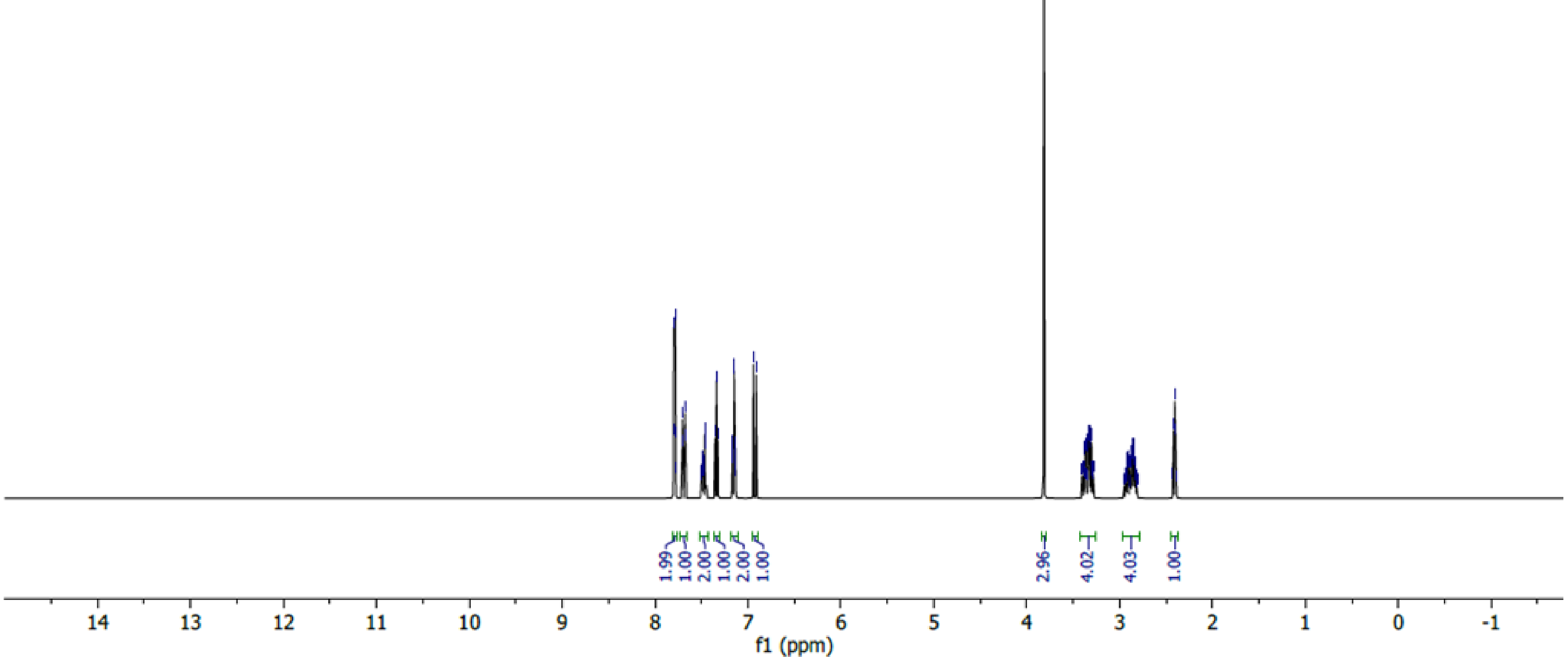
1.99
1.00
2.00
1.00
2.00
1.00
2.96
4.02
4.03
1.00
14
13
12
11
10
9
8
7
6
5
4
3
2
1
0
-1
f1 (ppm)

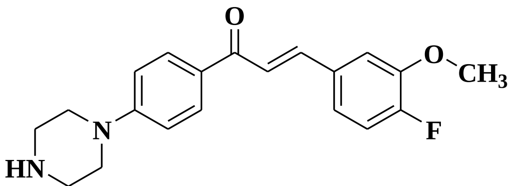
O
O
CH3
N
HN
F

Ve

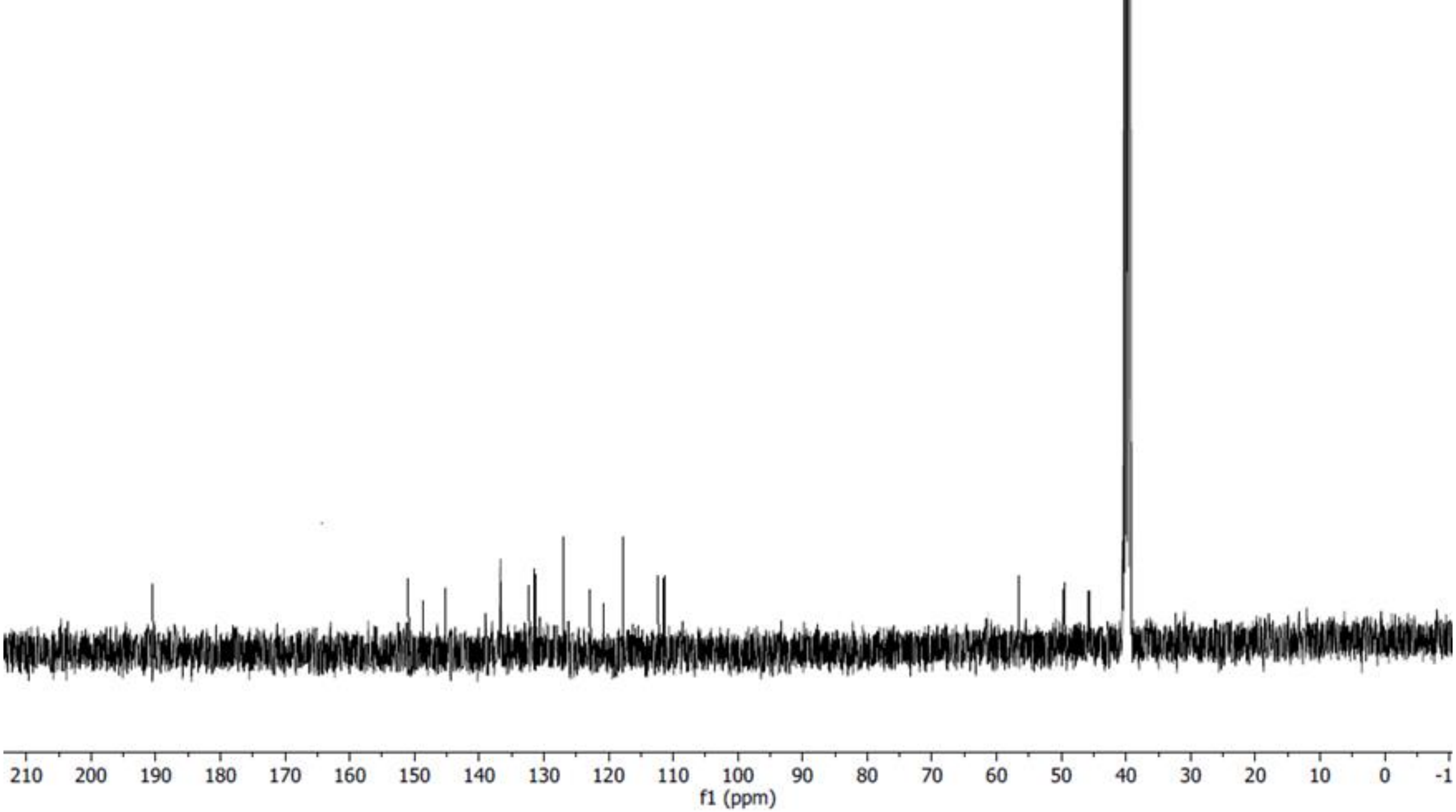
210 200 190 180 170 160 150 140 130 120 110 100 90 80 70 60 50 40 30 20 10 0 -1
f1 (ppm)

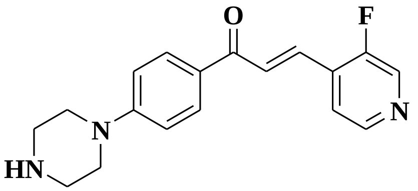
O
F
N
N
HN

Vf

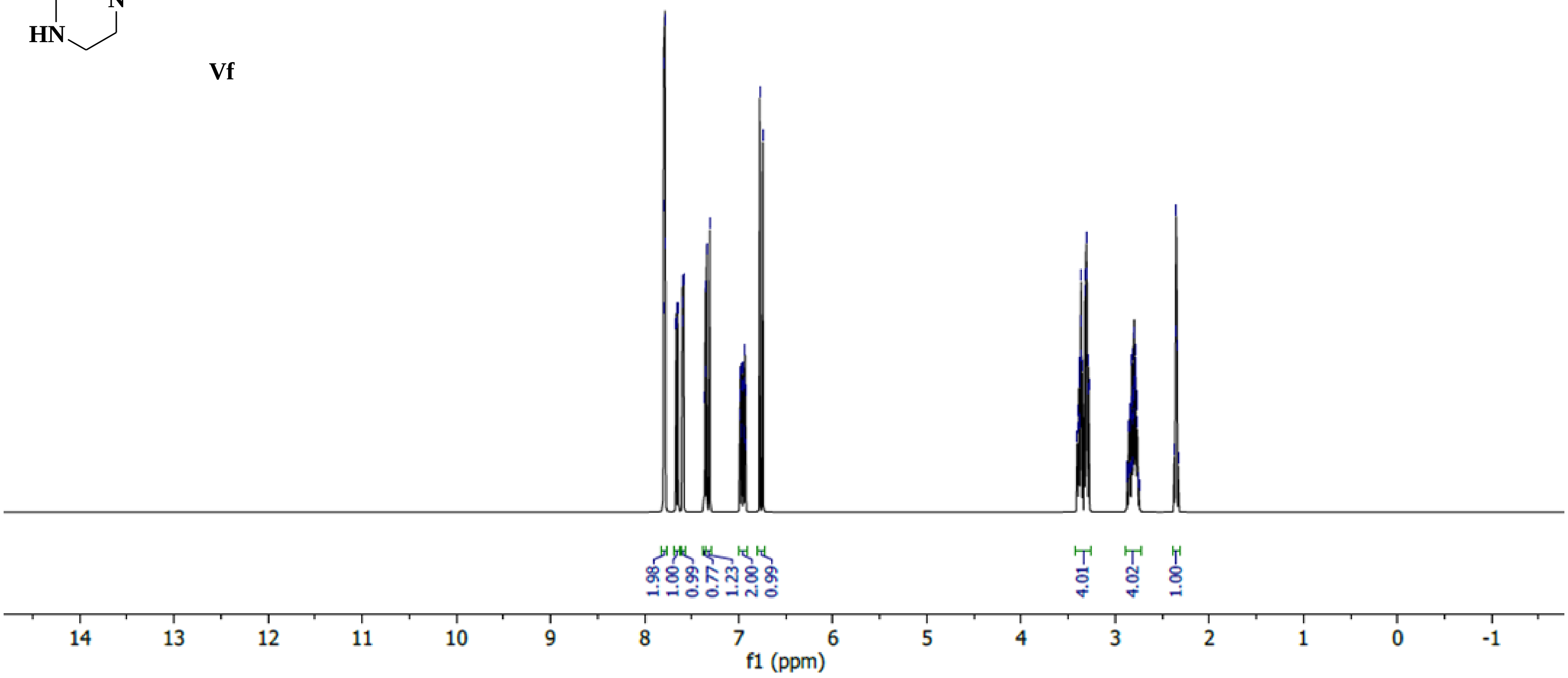
1.98
1.00
0.99
0.77
1.23
2.00
0.99
4.01
4.02
1.00
14
13
12
11
10
9
8
7
6
5
4
3
2
1
0
-1
f1 (ppm)

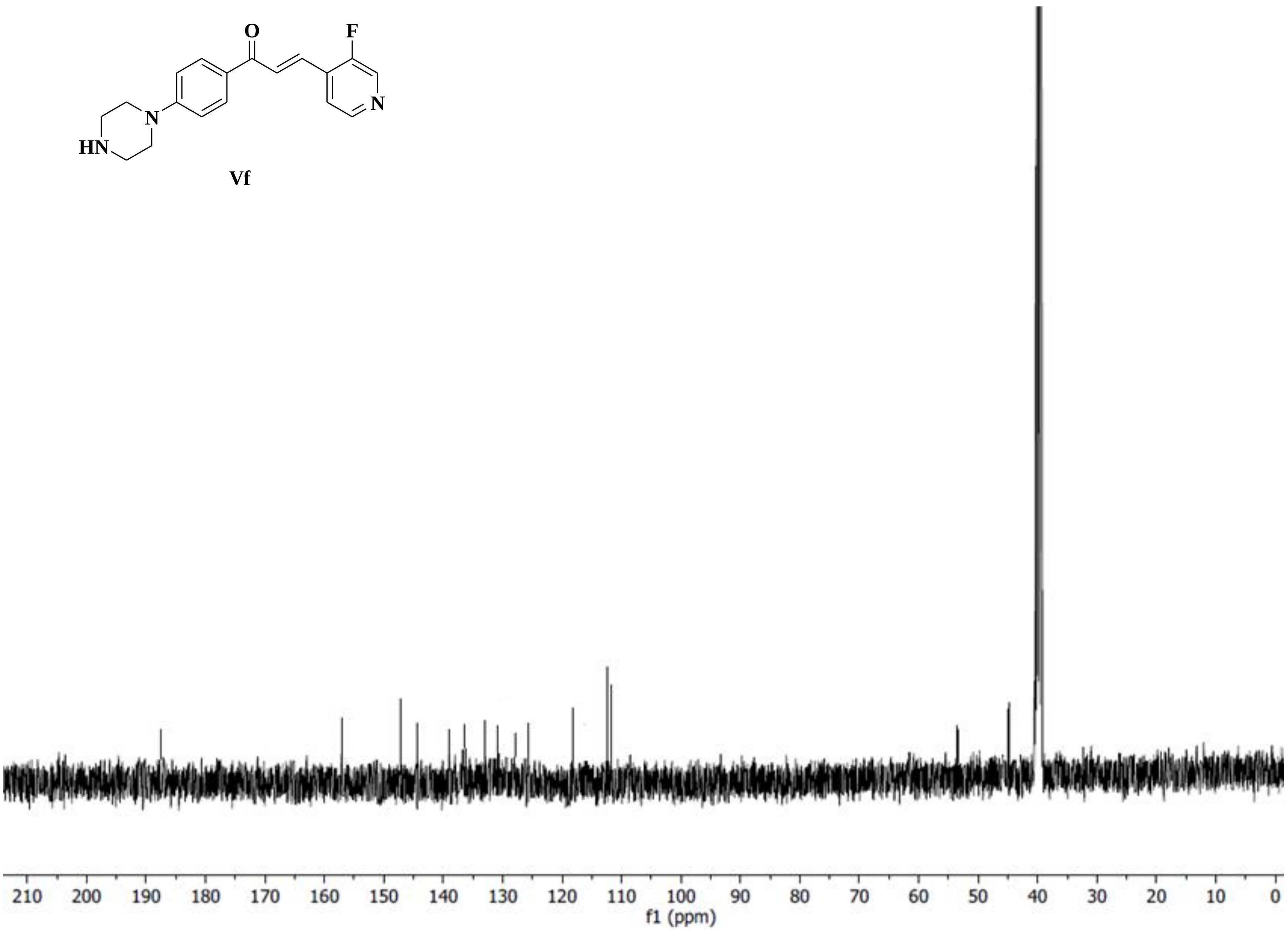
O
F
N
HN
N
Vf
210 200 190 180 170 160 150 140 130 120 110 100 90 80 70 60 50 40 30 20 10 0
f1 (ppm)

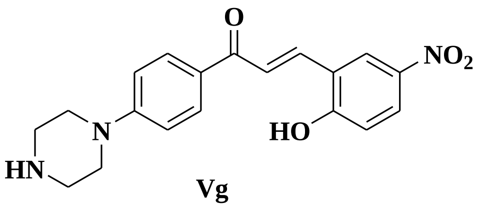
O
NO2
HO
N
HN

Vg

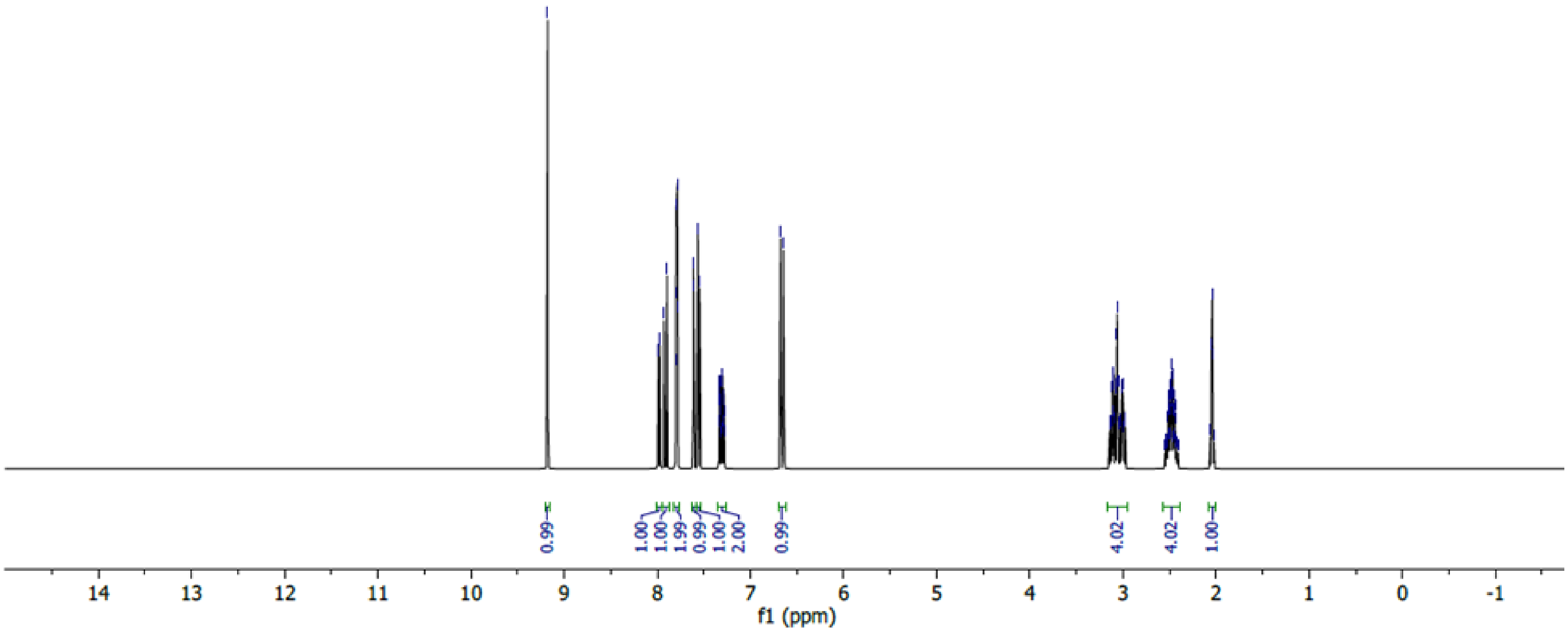
0.99
1.00
1.00
1.99
0.99
1.00
2.00
0.99
4.02
4.02
1.00
14 13 12 11 10 9 8 7 6 5 4 3 2 1 0 -1
f1 (ppm)

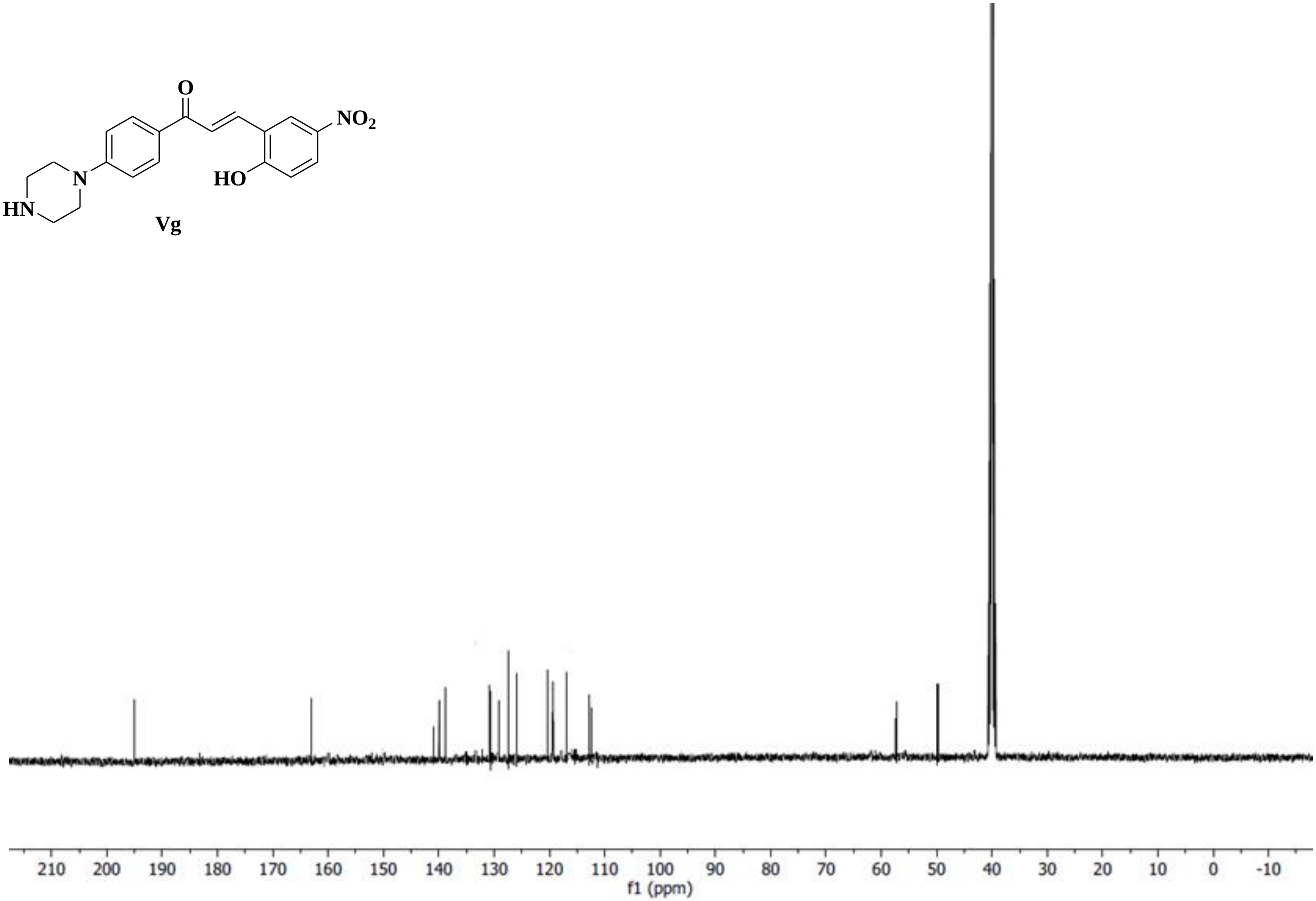
O
NO_2
HO
N
HN

Vg

210 200 190 180 170 160 150 140 130 120 110 100 90 80 70 60 50 40 30 20 10 0 -10
f1 (ppm)

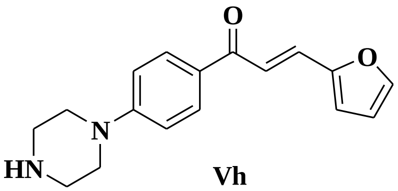
O
O
N
HN

Vh

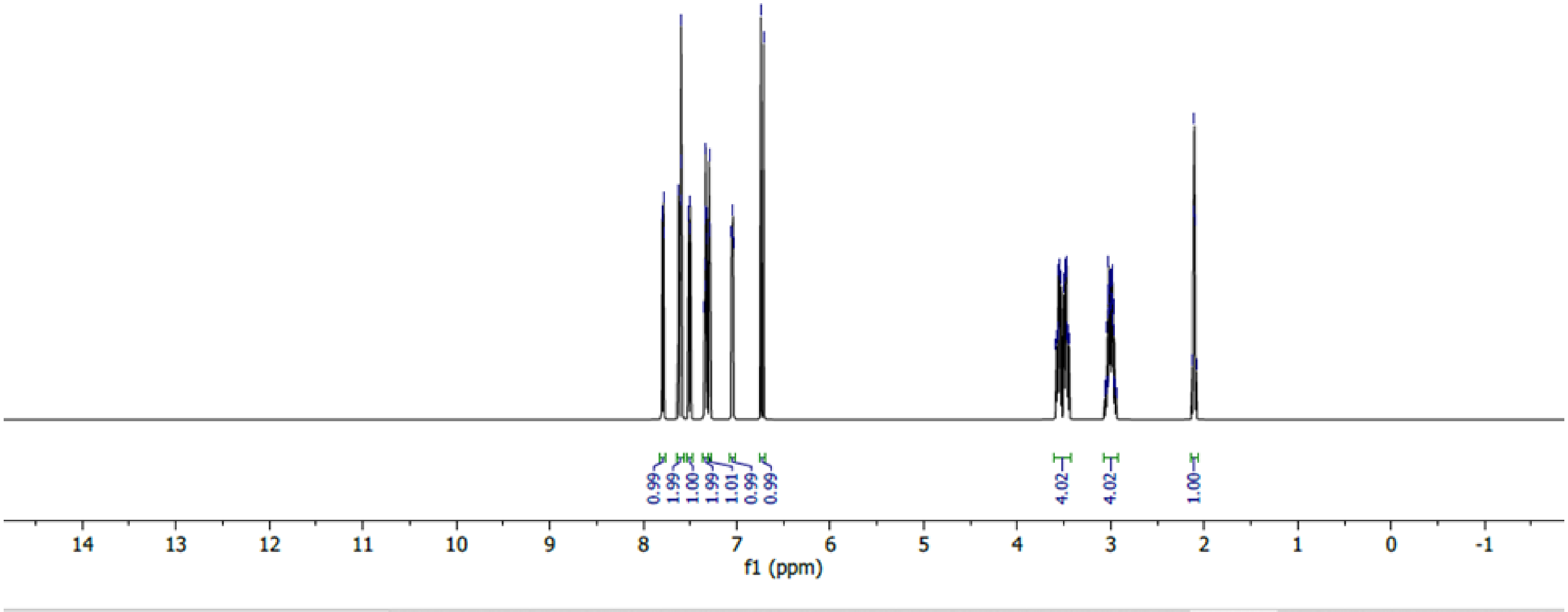
0.99
1.99
1.00
1.99
1.01
0.99
0.99
4.02
4.02
1.00
14
13
12
11
10
9
8
7
6
5
4
3
2
1
0
-1
f1 (ppm)

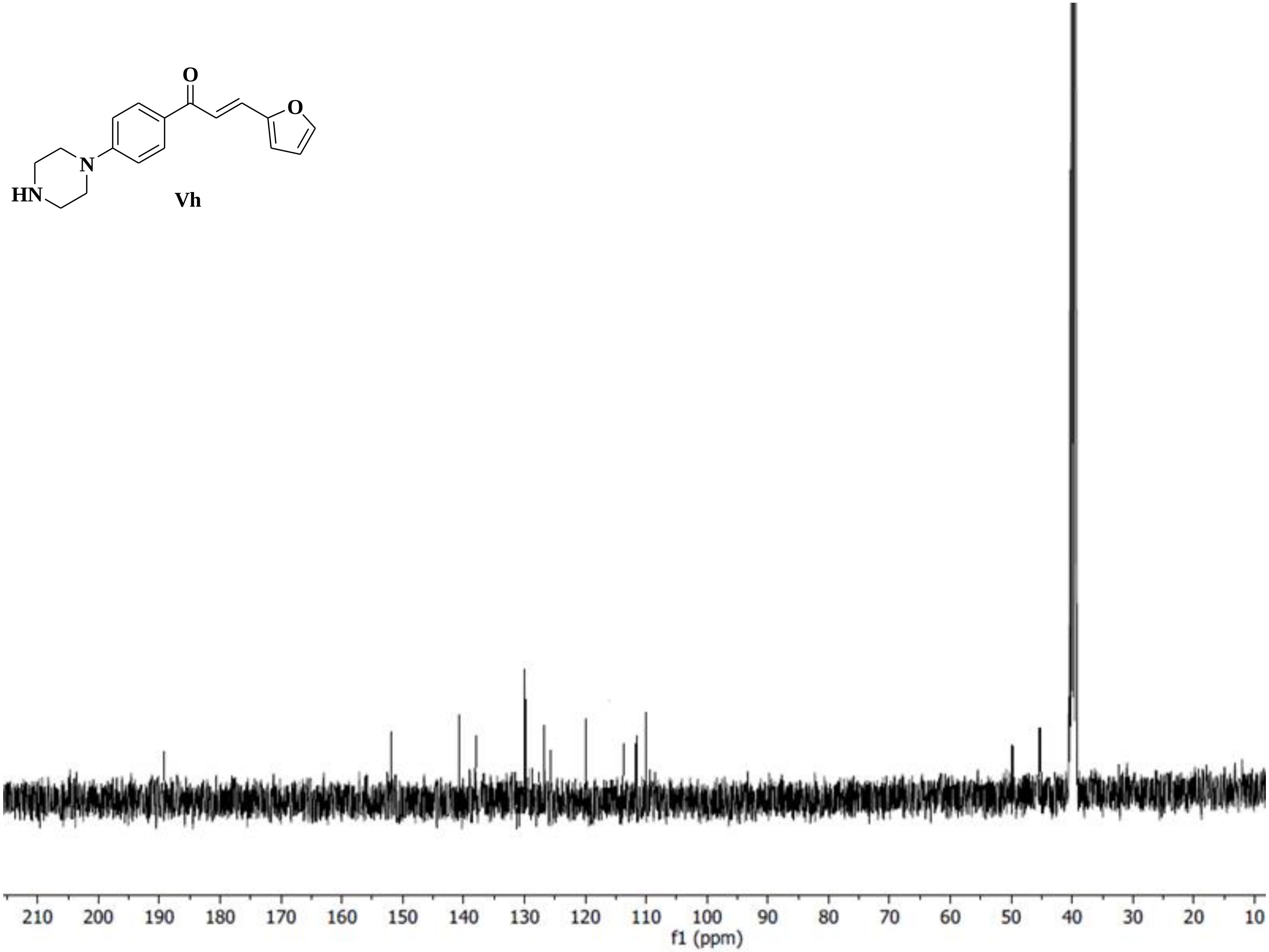

Vh

210 200 190 180 170 160 150 140 130 120 110 100 90 80 70 60 50 40 30 20 10
f1 (ppm)

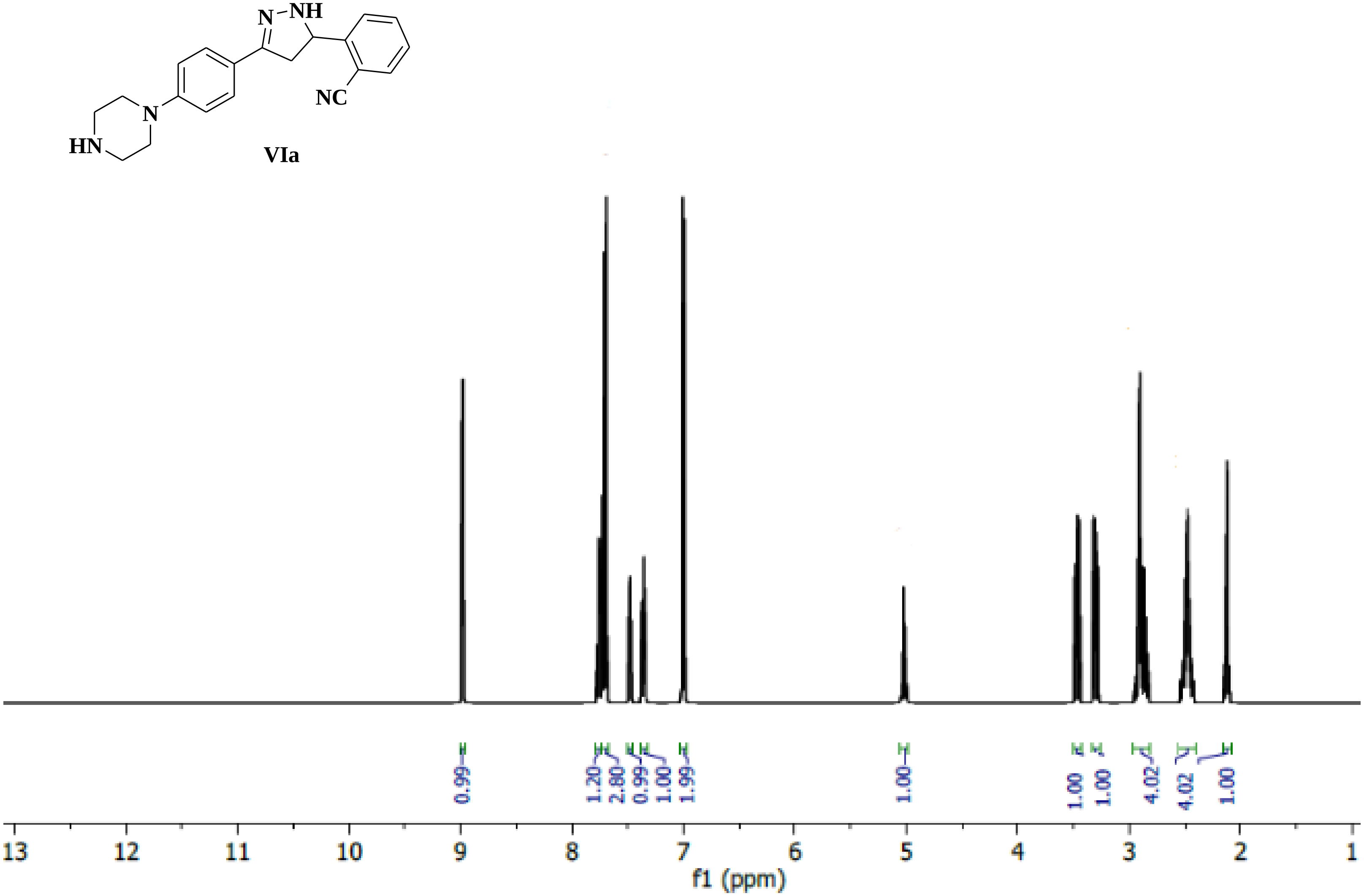
N
NH
NC
N
HN
VIa
0.99
1.20
2.80
0.99
1.00
1.99
1.00
1.00
1.00
4.02
4.02
1.00
13
12
11
10
9
8
7
6
5
4
3
2
1
f1 (ppm)

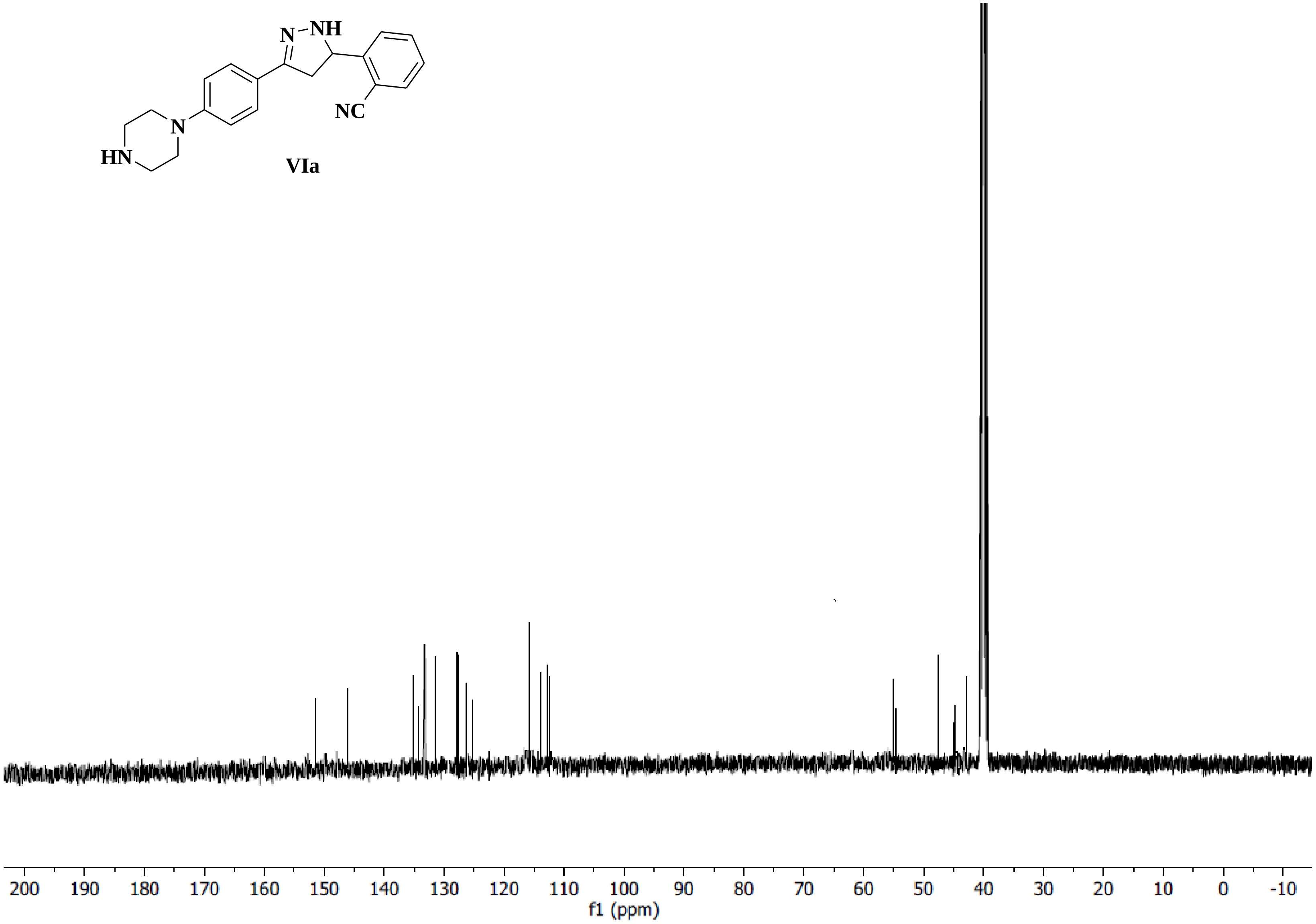
N
NH
HN
N
NC
VIa
200 190 180 170 160 150 140 130 120 110 100 90 80 70 60 50 40 30 20 10 0 -10
f1 (ppm)

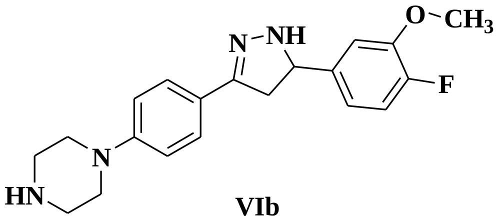
O
CH3
N
NH
F
N
HN

VIb

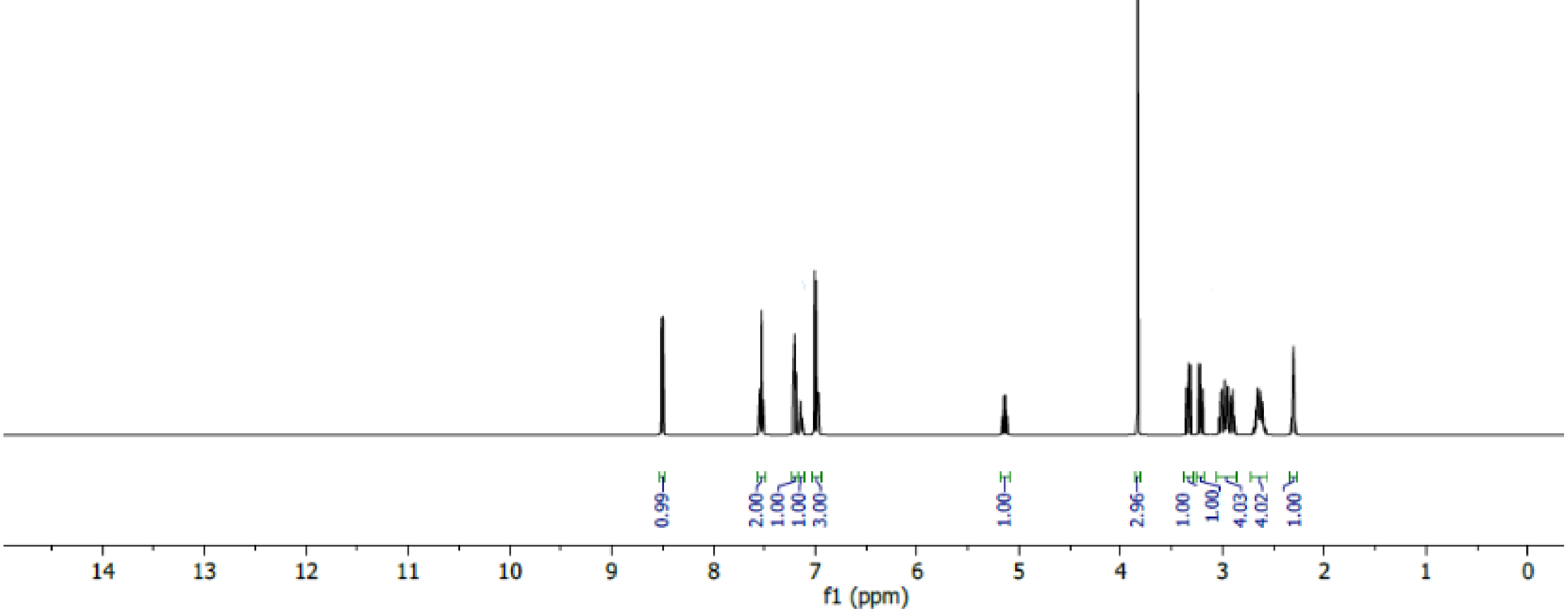
0.99
2.00
1.00
1.00
3.00
1.00
2.96
1.00
1.00
4.03
4.02
1.00
14
13
12
11
10
9
8
7
6
5
4
3
2
1
0
f1 (ppm)

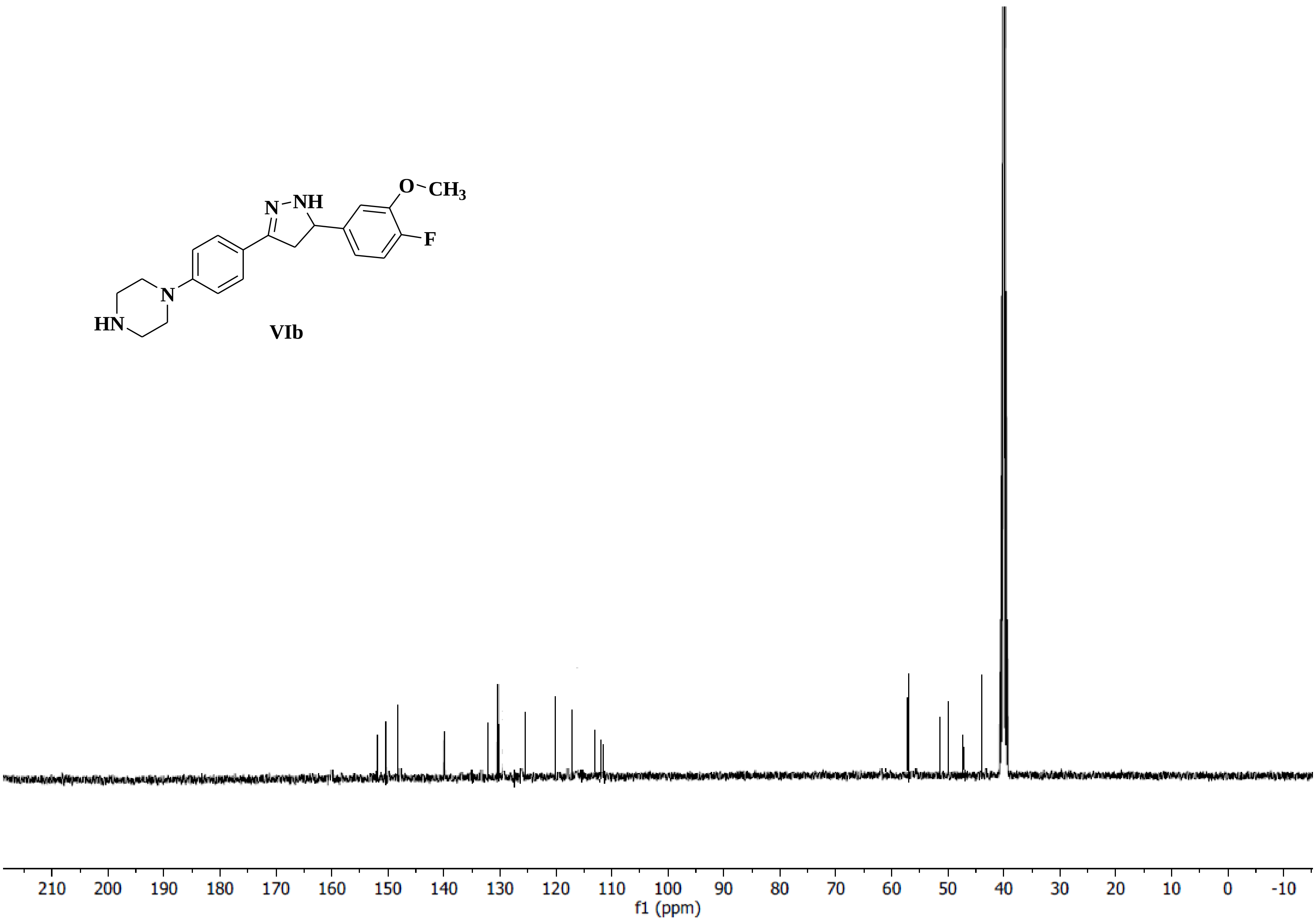
N-NH
O-CH3
F
N
HN
VIb
210 200 190 180 170 160 150 140 130 120 110 100 90 80 70 60 50 40 30 20 10 0 -10
f1 (ppm)

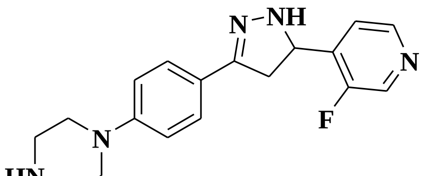
N
NH
N
F
N
HN

VIc

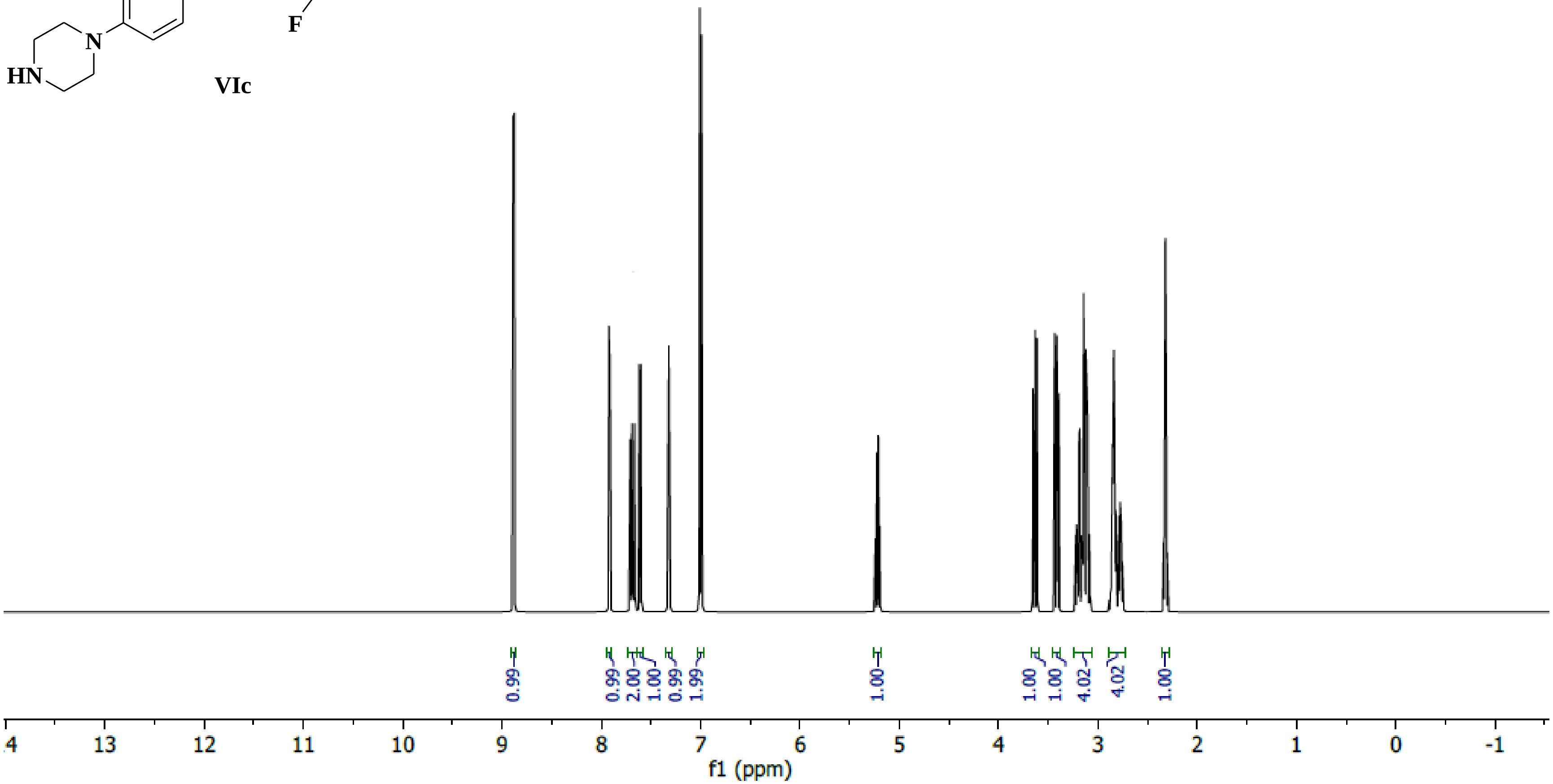
0.99
0.99
2.00
1.00
0.99
1.99
1.00
1.00
1.00
4.02
4.02
1.00
14
13
12
11
10
9
8
7
6
5
4
3
2
1
0
-1
f1 (ppm)

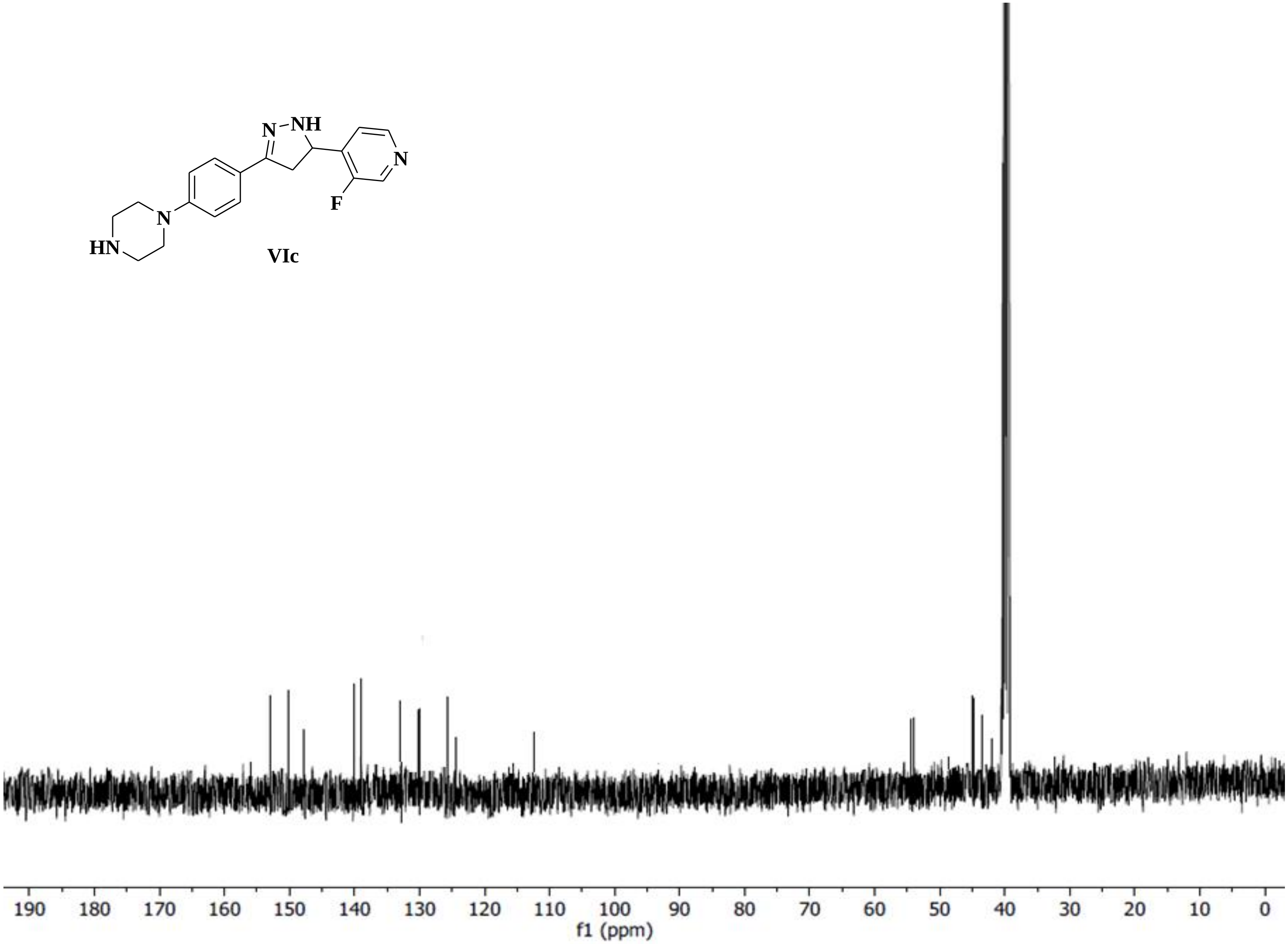
N
NH
N
F
HN
N
190 180 170 160 150 140 130 120 110 100 90 80 70 60 50 40 30 20 10 0
f1 (ppm)
VIc
